# Estimated Effects of Different Alcohol Taxation and Price Policies on Health Inequalities: A Mathematical Modelling Study

**DOI:** 10.1371/journal.pmed.1001963

**Published:** 2016-02-23

**Authors:** Petra S. Meier, John Holmes, Colin Angus, Abdallah K. Ally, Yang Meng, Alan Brennan

**Affiliations:** 1 Sheffield Alcohol Research Group, School of Health and Related Research, University of Sheffield, Sheffield, United Kingdom; 2 Bresmed Health Solutions, Sheffield, United Kingdom; Stanford University, UNITED STATES

## Abstract

**Introduction:**

While evidence that alcohol pricing policies reduce alcohol-related health harm is robust, and alcohol taxation increases are a WHO “best buy” intervention, there is a lack of research comparing the scale and distribution across society of health impacts arising from alternative tax and price policy options. The aim of this study is to test whether four common alcohol taxation and pricing strategies differ in their impact on health inequalities.

**Methods and Findings:**

An econometric epidemiological model was built with England 2014/2015 as the setting. Four pricing strategies implemented on top of the current tax were equalised to give the same 4.3% population-wide reduction in total alcohol-related mortality: current tax increase, a 13.4% all-product duty increase under the current UK system; a value-based tax, a 4.0% ad valorem tax based on product price; a strength-based tax, a volumetric tax of £0.22 per UK alcohol unit (= 8 g of ethanol); and minimum unit pricing, a minimum price threshold of £0.50 per unit, below which alcohol cannot be sold. Model inputs were calculated by combining data from representative household surveys on alcohol purchasing and consumption, administrative and healthcare data on 43 alcohol-attributable diseases, and published price elasticities and relative risk functions. Outcomes were annual per capita consumption, consumer spending, and alcohol-related deaths. Uncertainty was assessed via partial probabilistic sensitivity analysis (PSA) and scenario analysis.

The pricing strategies differ as to how effects are distributed across the population, and, from a public health perspective, heavy drinkers in routine/manual occupations are a key group as they are at greatest risk of health harm from their drinking. Strength-based taxation and minimum unit pricing would have greater effects on mortality among drinkers in routine/manual occupations (particularly for heavy drinkers, where the estimated policy effects on mortality rates are as follows: current tax increase, −3.2%; value-based tax, −2.9%; strength-based tax, −6.1%; minimum unit pricing, −7.8%) and lesser impacts among drinkers in professional/managerial occupations (for heavy drinkers: current tax increase, −1.3%; value-based tax, −1.4%; strength-based tax, +0.2%; minimum unit pricing, +0.8%). Results from the PSA give slightly greater mean effects for both the routine/manual (current tax increase, −3.6% [95% uncertainty interval (UI) −6.1%, −0.6%]; value-based tax, −3.3% [UI −5.1%, −1.7%]; strength-based tax, −7.5% [UI −13.7%, −3.9%]; minimum unit pricing, −10.3% [UI −10.3%, −7.0%]) and professional/managerial occupation groups (current tax increase, −1.8% [UI −4.7%, +1.6%]; value-based tax, −1.9% [UI −3.6%, +0.4%]; strength-based tax, −0.8% [UI −6.9%, +4.0%]; minimum unit pricing, −0.7% [UI −5.6%, +3.6%]). Impacts of price changes on moderate drinkers were small regardless of income or socioeconomic group. Analysis of uncertainty shows that the relative effectiveness of the four policies is fairly stable, although uncertainty in the absolute scale of effects exists. Volumetric taxation and minimum unit pricing consistently outperform increasing the current tax or adding an ad valorem tax in terms of reducing mortality among the heaviest drinkers and reducing alcohol-related health inequalities (e.g., in the routine/manual occupation group, volumetric taxation reduces deaths more than increasing the current tax in 26 out of 30 probabilistic runs, minimum unit pricing reduces deaths more than volumetric tax in 21 out of 30 runs, and minimum unit pricing reduces deaths more than increasing the current tax in 30 out of 30 runs). Study limitations include reducing model complexity by not considering a largely ineffective ban on below-tax alcohol sales, special duty rates covering only small shares of the market, and the impact of tax fraud or retailer non-compliance with minimum unit prices.

**Conclusions:**

Our model estimates that, compared to tax increases under the current system or introducing taxation based on product value, alcohol-content-based taxation or minimum unit pricing would lead to larger reductions in health inequalities across income groups. We also estimate that alcohol-content-based taxation and minimum unit pricing would have the largest impact on harmful drinking, with minimal effects on those drinking in moderation.

## Abstract 2

In this mathematical modelling study, Petra Meier and colleagues estimate the impact of different alcohol taxation and pricing structures on consumption, spending, and health inequalities.

## Editors' Summary

**Background:**

People have drunk alcoholic beverages throughout history. However, harmful alcohol consumption is currently responsible for around 2.7 million deaths every year and is a leading risk factor worldwide for heart disease, liver disease, and many other health problems. It also affects the well-being and health of people around those who drink, both within the household and through alcohol-related crime and road traffic crashes. As with most products, the price of alcohol influences how much people buy and consume. Alcohol affordability is an important driver of alcohol consumption, and in many countries, including the UK and US, alcohol prices have not kept pace with inflation and rising incomes. Alcohol taxes have the dual function of raising revenues and regulating alcohol prices. Different countries employ different alcohol-specific taxation structures including taxation by beverage volume, by value, or by alcohol content. Some countries also have additional price control measures that prevent the sale of very cheap alcohol.

**Why Was This Study Done?:**

Although research shows that increases in the price of alcohol brought about by taxation or other pricing policies reduce alcohol consumption and alcohol-related harm, little is known about how alternative tax and price policy options affect the scale and distribution of alcohol-related health impacts across society. This is important because people with lower social status and/or income are disproportionately affected by alcohol-related disease. An effective public health policy to reduce alcohol harm might, therefore, help to reduce social disparities in health (health inequality), a major goal of population health policies in affluent countries.

**What Did the Researchers Do and Find?:**

Here, the researchers use the Sheffield Alcohol Policy Model (SAPM) to investigate the effects of four common alcohol taxation and price policies on health inequalities in England during 2014/2015. SAPM is a deterministic mathematical simulation model that estimates how price changes affect individual-level alcohol consumption and how consumption changes affect the illnesses (morbidity), deaths (mortality), and economic costs associated with 43 alcohol-attributable conditions. The researchers used SAPM to simulate the effect of increasing the existing UK duty on all alcohol products by 13.4% (current tax increase), introducing a 4% tax based on product price (value-based, or ad valorem, taxation), introducing a tax of £0.22 per alcohol unit (strength-based, or volumetric, taxation), or setting a minimum price threshold of £0.50 per alcohol unit (minimum unit pricing). The magnitudes of the different policy-induced price increases modeled were chosen to result in the same overall population-wide 4.3% decrease in alcohol-related mortality. Notably, the impacts of policy changes on moderate drinkers were small, regardless of income/socioeconomic group. However, among heavy drinkers, the effects of the four policies were differentially distributed across the population. Among heavy drinkers in the lowest socioeconomic group (the population group at greatest risk of harm from alcohol use), the estimated effects on mortality rates were −3.2% for the current tax increase (that is, it reduced alcohol-related deaths by 3.2%), −2.9% for value-based taxation, −6.1% for strength-based taxation, and −7.8% for minimum unit pricing. Among heavy drinkers in the highest socioeconomic group, the corresponding effects on mortality rates were −1.3%, −1.4%, +0.2%, and +0.8%.

**What Do These Findings Mean?:**

As with any policy modelling, the accuracy of these findings depends on the evidence base, the quality of the data incorporated into the model, and the assumptions used. Limitations to the analysis here include that, due to an absence of evidence, the researchers have not examined the impact of any tax avoidance, which could potentially vary between the policies. The study findings suggest that in England (and probably in other countries) the introduction of strength-based taxation or minimum unit pricing would lead to larger reductions in health inequalities among heavy drinkers than an increase in the current tax rate or the introduction of value-based taxation. That is, the two policy options that target cheap, high-strength alcohol are likely to outperform value-based taxation and increasing the current UK tax in terms of reducing health inequalities. Thus, although these policies might be considered “regressive” (i.e., affecting the poor more than the rich) in terms of consumption and spending, they are at the same time “progressive” in that they reduce health inequalities. Finally, these findings suggest that minimum unit pricing and strength-based taxation, unlike the other two options tested, would target harmful drinking without unnecessarily penalizing people with low incomes who drink moderate amounts of alcohol.

**Additional Information:**

This list of resources contains links that can be accessed when viewing the PDF on a device or via the online version of the article at http://dx.doi.org/10.1371/journal.pmed.1001963.

- The World Health Organization provides detailed information about alcohol, including a fact sheet on the harmful use of alcohol; its *Global Status Report on Alcohol and Health 2014* provides country information on the impact of alcohol use on health and policy responses; its *Global Strategy to Reduce Harmful Use of Alcohol* includes information on pricing policies; the Global Information System on Alcohol and Health provides further information about alcohol control policies
- The US National Institute on Alcohol Abuse and Alcoholism has information about alcohol and its effects on health; it provides interactive worksheets to help people evaluate their drinking and decide whether and how to make a change
- The US Centers for Disease Control and Prevention provides information on alcohol and public health and a fact sheet on preventing excessive alcohol use
- The UK National Health Service Choices website provides detailed information about drinking and alcohol, including information on the risks of drinking too much, tools for calculating alcohol consumption, and personal stories
- EuroCare is an alliance of non-governmental public health and social organizations working on the prevention and reduction of alcohol-related harm in Europe; it provides information about alcohol taxation in the European Union
- The UK Institute of Alcohol Studies advocates for the use of scientific evidence in policymaking to reduce alcohol-related harm and produces easily accessible briefings on alcohol policy issues
- MedlinePlus provides links to many other resources on alcohol
- Information about the Sheffield Alcohol Policy Model is available
- The UK Chief Medical Officers’ proposed new alcohol guidelines are available

## Introduction

Harmful alcohol consumption is a major public health issue accounting for an estimated 2.7 million deaths globally, and, together with high blood pressure, smoking, air pollution, and unhealthy diet, it is ranked among the five leading contributors to the global burden of disease [1]. Estimates of the economic costs attributable to alcohol amount to more than 1% of gross domestic product in high-income and middle-income countries [2]. There is also a substantial social gradient in the burden of disease attributable to alcohol, such that it is disproportionately concentrated in disadvantaged groups. Thus, the most effective public policies to reduce alcohol harm are likely to reduce persistent and growing disparities in health, a major goal of population health policies in developed countries [3,4].

Links between alcohol price increases and reductions in consumption [5–7], alcohol-related mortality [8], and healthcare costs [9,10] are well established. Consequently, the World Health Organization (WHO) recommends alcohol taxation as amongst the most cost-effective options for tackling alcohol-related ill health [10]. Of 167 countries responding to a WHO survey in 2010, 154 collected some form of alcohol taxes [11], although the way these taxes were calculated differs markedly between countries and states. Three dominant tax structures are used internationally, either singly or in combination: (1) ad valorem tax (proportionate to product value), a component of alcohol taxes in 96 countries; (2) volumetric tax (based on product strength/ethanol content), a tax component in 50 countries and (3) unitary tax (based on product volume), a tax component in 41 countries [11]. Examples of tax structures are given in Box 1.

**Box 1. Examples of Different Alcohol-Specific Taxation Structures**

**Australia:** Beer and spirits are taxed by ethanol content (with strength-based thresholds for beer and a lower rate for brandy than for other spirits); wine and cider are taxed by value.

**Germany:** Beer and spirits are taxed by ethanol content; sparkling wine is taxed by volume; no duty is levied on still wine.

**South Korea:** Spirits are taxed by volume; all other alcohol is taxed by value.

**Mexico:** All beverage types are taxed by value (with strength-based tax bands).

**UK:** Spirits and beer are taxed by ethanol content (with strength-based tax bands for beer); wine and cider are taxed by volume.

Several countries employ or are considering additional price-based alcohol control measures alongside taxation. In particular, an alcohol-content-based minimum price (or floor price) below which products cannot be sold is in force in parts of Canada and the Russian Federation and has been proposed in Ireland, the UK, Switzerland, New Zealand, and Australia.

Differential effects of alcohol pricing policies across socioeconomic groups and the resulting impacts on health inequalities are of great importance to both the political and legal debate. Politically, the alcohol industry and policy opponents argue that alcohol taxation and minimum pricing “penalise” low-income moderate drinkers and are thus regressive and poorly targeted [12]. From a legal perspective, the comparative effectiveness of different price-based public health interventions across outcomes including health inequalities is a key issue in European law. The European Union Treaty of Rome permits distortions of free trade resulting from new price-based public health interventions where, in achieving their aims, these interventions lead to additional advantages or fewer disadvantages to either public health or free trade than would result from using pre-existing interventions such as taxation [13]. Arguments on this point lie at the centre of the ongoing legal dispute between the Scottish Government and the Scotch Whisky Association over minimum unit pricing (setting a minimum price per UK alcohol unit [8 g of pure ethanol]) and have relevance in Ireland, where minimum unit pricing is part of the Public Health (Alcohol) Bill 2015, and England, where the policy remains “under consideration”. Empirical evidence comparing the effects of different alcohol pricing policies is currently lacking. While there is evidence about the equity implications of minimum unit pricing policies [14], and recent evidence from a US state tax evaluation points to comparatively larger effects on the health of disadvantaged groups [15], tax policy appraisals have to date focussed on government revenue, consumption changes, and consumer spending, without considering impacts on public health and health inequalities [16–21].

Using England 2014/2015 as a case study, this paper sets out to appraise the relative public health merits of minimum unit pricing and three of the most common tax structures globally. It will provide estimated effects on alcohol consumption, consumer spending, and alcohol-attributable mortality for the whole adult population and for subgroups defined by consumption level and socioeconomic position.

## Methods

### Overview

The Sheffield Alcohol Policy Model (SAPM), version 3, is a deterministic mathematical simulation model providing a comprehensive framework for appraising UK and international policy options [22]. It comprises an individual-based econometric component that estimates how price changes affect individual-level alcohol consumption, and a cohort-based dynamic epidemiological component that estimates how consumption changes affect the morbidity, mortality, and economic costs associated with 43 alcohol-attributable conditions. A full description of the methodology and the data sources used is provided in S1 Appendix and S1 Data.

### Current Tax System and Modelled Policies

We model four common alcohol taxation and price policy options (see Box 2), each implemented on top of the current system of excise duty and value added tax (VAT) in England [23]. In England, duty rates for wine and cider are charged per hectolitre of product (not linked to alcoholic strength); beer, spirits, and spirits-based alcopops are taxed by ethanol volume. Final prices paid by consumers at the till include a 20% VAT, which is levied on all non-exempt products and services and therefore not specific to alcohol. Special rates for very low strength (≤2.8% alcohol by volume [ABV]) and high strength (>7.5% ABV) beers and ciders were not modelled, as their market share is very small [24].

**Box 2. Specification of the Modelled Policies**

**Current tax increase:** Raise current alcohol duty for all beverage categories by 13.4%.

**Ad valorem tax:** Introduce an additional 4.0% alcohol-specific sales tax on product value after duty at the time of purchase (at which point the standard 20% VAT is also charged on product (product value + ad valorem tax).

**Volumetric tax:** Replace current excise duty with a duty of £0.22 per unit for all beverage types (UK unit = 8 g ethanol).

**Minimum unit pricing:** Introduce a floor price of £0.50 per alcohol unit within the current tax system.

**As implemented in the model**

**Basecase (current taxation):** Price_0_ = (net price for retailer + existing beverage-specific duty) × (100% + 20% VAT).

**Current tax increase:** Price_1_ = (net price for retailer + existing beverage-specific duty × [100% + 13.4%]) × (100% + 20% VAT).

**Ad valorem tax:** Price_2_ = (net price for retailer + existing beverage-specific duty) × (100% + 4.0%) × (100% + 20% VAT).

**Volumetric tax:** Price_3_ = (net price for retailer + £0.22 × number of alcohol units in product) × (100% + 20% VAT).

**Minimum unit pricing:** Price_4_ = maximum of Price_0_ or (£0.50 × number of alcohol units in product).

There is no change to any taxes in the rest of the supply chain, either at the production or wholesale stages.

The focus of this paper is the differential effects of the four policies on population subgroups defined by drinking level and income (for purchasing and consumption analyses) or socioeconomic group (for health and health inequalities analyses; see below for more detail). As each policy can be applied at different levels (e.g., higher or lower taxation/minimum unit price), a uniform level needs to be identified to allow a valid comparison across policies. For political relevance, we chose the level of each policy that would achieve a 4.3% reduction in population-level mortality as this is the estimated impact of a £0.50 minimum unit price. This level of minimum unit pricing is a policy that is being considered or in the process of implementation by all four UK governments, and a key political and legal question is whether existing tax powers can achieve comparable effects.

### Policy to Price

For details of sources of all model inputs, see S1 Data. The 2001–2009 Living Costs and Food Surveys (LCF) [25] provide diary data for 227,933 on- and off-trade alcohol purchases by 46,163 individuals in England and also provide information on respondents’ equivalised household income (i.e., income adjusted to account for household size and structure). Baseline price paid distributions (volumes purchased across price points) are calculated for ten modelled beverage categories, which include beverage type and the location of purchase—beer, cider, wine, spirits, and ready-to-drink beverages purchased either in the on-trade (i.e., in pubs, bars, clubs, and restaurants) or off-trade (i.e., in shops including off-licenses and supermarkets) sector—across population subgroups defined by age, gender, drinking level, and income. Market research data from Nielsen and CGA Strategy were used to align LCF data with more robust population-level price distributions (see S1 Appendix, Section 4.4). Office for National Statistics alcohol-specific price indices were used to inflate to 2014 prices. For reporting, ready-to-drink beverages are included with spirits as the vast majority of ready-to-drink beverages sold in the UK fall within that category for taxation purposes.

To estimate the impact of tax changes on the price distribution of alcohol sold in England, three steps are taken. First, we calculate the change in price for every individual LCF purchase implied by the pricing policy. Second, we account for evidence from our previous analyses that alcohol tax rises are not always passed on fully to consumers, particularly on cheaper products, where part of the tax rise appears to be absorbed by retailers or their suppliers, a practice known as undershifting [26]. Finally, we use the revised prices to generate new price distributions for each beverage category and population subgroup.

### Price to Consumption

#### Baseline consumption data

The 2012 Health Survey for England (HSE) [27] provides data on beverage-specific mean weekly and maximum daily alcohol consumption (consumption on heaviest drinking day in the survey week) for a nationally representative sample of 6,394 individuals. The data were used to represent baseline consumption preferences for 120 population subgroups defined by gender, age (16–24, 25–34, 35–54 and 55+ y), consumption level based on the English drinking guidelines prior to their revision in January 2016 (see Tables 1 and 2), and income (quintiles based on household income equivalised to account for household size and structure). The HSE does not separate consumption into off- and on-trade, and combines beer and cider, so each subgroup’s distribution of purchasing across these categories in the LCF was used to apportion the corresponding subgroup’s consumption in the HSE. Sensitivity analyses relating to this apportioning are reported elsewhere [14].

**Table 1:** Weekly consumption thresholds for drinking level groups.

Drinking Level Group	UK Alcohol Units per Week		Equivalent Grams of Ethanol per Week	
	Men	Women	Men	Women
Moderate	≤21	≤14	≤166 g	≤111 g
Increasing risk	>21 and ≤50	>14 and ≤35	>166 and ≤395 g	>111 and ≤277 g
Heavy	>50	>35	>395 g	>277 g

**Table 2:** UK alcohol unit content of popular drinks (1 unit = 8 g [10 ml] of pure alcohol).

Drink	UK Alcohol Unit Content
1 imperial pint (568 ml) of standard beer (4%)	2.3 units
1 small bottle (330 ml) of standard premium beer (5%)	1.7 units
1 small glass (125 ml) of wine (12%)	1.5 units
1 standard glass (175 ml) of wine (13%)	2.3 units
1 large glass (250 ml) of wine (14%)	3.5 units
1 single shot (25 ml) of spirits (40% ABV)	1 unit

#### Price elasticities

Price elasticities estimate how price changes in a product relate to purchasing changes of the same and of related products (e.g., after a beer price increase, beer consumption may decrease but spirits consumption may increase). Recently published UK population-level own- and cross-price elasticities for alcohol demand in the ten beverage categories were used to model the relationship between price and consumption changes [28]. Each subgroup in the population is therefore modelled as having a different overall alcohol price elasticity due to (1) each beverage category having different own-price and cross-price elasticities and (2) each subgroup having different proportions of their consumption within each beverage category. It is also worth noting that different subgroups also face different price changes due to variation in their purchasing preferences. Whilst the use of income- or consumption-group-specific elasticities may seem appealing, the inclusion of such factors violates the assumptions underlying the pseudo-panel methodology used to estimate the UK elasticities, and we have therefore moved away from producing and using such estimates. However, as reported in sensitivity analyses in the technical appendices of previous papers that used an alternative methodology for estimating elasticities [14,29], the use of such disaggregated elasticities did not change the direction of differences or overall conclusions.

To model policy effects on consumption, we use the revised price distributions to compute the average percentage change in price within each beverage category and population subgroup. We then apply the price elasticities to derive a percentage change in consumption for each beverage category and subgroup. Finally, we apply this percentage change in consumption to each individual within each subgroup, where subgroups are defined by age, gender, drinking level, and income quintile. We do not model transitions between drinking groups, so where we report on moderate drinkers, this refers to those meeting moderate drinking thresholds at baseline rather than those who may have decreased their drinking to moderate levels in response to policy. This does not, however, mean that their consumption is restricted to remain within the bounds of the definition of moderate drinking. Note that as we deal with relative changes, we also do not model any transition between drinkers and abstainers. This is likely to mean that our model underestimates the true effects of all four modelled policies as some consumers may give up alcohol altogether in response to price increases.

### Consumption to Harm

Having calculated changes in individual-level alcohol consumption over time, SAPM then aggregates the modelled population into cohorts defined by age, gender, consumption level, and socioeconomic group (three occupational groups: professional/managerial, intermediate, and routine/manual/unemployed). The model then uses separate risk functions for 43 alcohol-attributable diseases and injuries to model the impacts of consumption changes on alcohol harm within each cohort, including diseases (diabetes, ischaemic heart disease, and ischaemic stroke) where alcohol may have beneficial effects for some groups (see S1 Appendix, Section 8.1).

For 14 chronic conditions partly attributable to alcohol (e.g., alcohol-related cancers), we used risk functions from recent high-quality meta-analyses (see S1 Appendix, Section 8.1). For nine partly attributable acute conditions (e.g., fall injuries), functions were estimated by linking measures of consumption frequency, average occasion-specific consumption, and consumption variability across occasions with injury risk [30,31]. For ten chronic and seven acute conditions wholly attributable to alcohol (e.g., alcoholic liver disease, alcohol poisoning), we fitted linear functions relating average or maximum daily consumption to absolute numbers of cases reported (see S1 Appendix, Section 9.3.3).

The model operates in 1-y cycles: within each year the 43 risk functions are used to calculate the condition-specific alcohol-attributable mortality risk for individuals within each cohort given their consumption level. This risk is aggregated across all individuals in the cohort and compared to the baseline risk under pre-policy consumption levels, and the ratio between the two is used to adjust the baseline mortality rate and estimate the corresponding number of deaths for that cohort and condition in that year. Baseline population demographics and all-cause mortality rates (used to estimate background mortality from other, non-alcohol-related causes) are taken from figures published by the Office for National Statistics (see S1 Data). Baseline annual gender- and age-specific mortality data for each condition are taken from recent English analyses [32] and apportioned between socioeconomic groups using published evidence on socioeconomic gradients in mortality [33]. All consumption changes are assumed to occur in the first year after intervention, but delays (time lags) between alcohol consumption changes and effects on population-level harm are commonly observed and vary by disease. Lag parameters were taken from a recent systematic review [34] (see table in S1 Appendix, Section 9.1). Maximum intervention effects in the model are reached after 20 y (the longest identified lag time), and all harm results are reported for the 20th year following policy implementation. Mortality results for years 1–19 are reported in S1 Appendix, Section 10.1.

### Socioeconomic Position

There are two separate steps in the analysis which use different measures of socioeconomic position. For the model relating price changes to consumption changes, we split the population into income quintiles based on respondents’ equivalised household income. This approach reflects that disposable income (rather than socioeconomic group) is considered a key driver of alcohol purchasing. There is, however, reason to believe that differences in socioeconomic group may drive health inequalities more than income. Also, unlike for income, condition-specific mortality rates stratified by the National Statistics Socio-economic Classification (NS-SEC) are available (see S1 Data), and so we model the effect of alcohol consumption changes on deaths using socioeconomic group as the stratification variable. The HSE contains both income and NS-SEC information for each individual, meaning no mapping between data sources is required and no assumption is made regarding the relationship between these two measures of socioeconomic position.

### Sensitivity Analyses

SAPM version 3 is a fully deterministic model that does not produce confidence intervals around its results. This is in large part due to the vast majority of the data sources used in the model not providing estimates of uncertainty. However, we acknowledge the importance of exploring the potential impact of such parameter uncertainty on model results and have previously undertaken extensive scenario analyses to this end. These analyses suggest that our model estimates tend to be conservative and that using alternative assumptions would not change the major conclusions of the model (e.g., [14,35]). For the present study we have taken a new approach to characterising uncertainty in our results. Whilst we do not believe a full probabilistic sensitivity analysis (PSA) is feasible, given the lack of parameterisation of uncertainty available in much of the model input data, we have carried out partial PSAs around a number of the key model inputs. For each of 30 PSA model runs, we drew a random nonparametric bootstrap sample from the baseline consumption and purchasing survey datasets. We also sampled the elasticities and tax pass-through parameters from the relevant distributions given in their derivation. Finally, we used the limited available data on uncertainty around published risk functions to estimate a parameterisation of uncertainty around the risk functions used in the model. For each sampled set of model inputs, we ran all four modelled policies. As a further exploration of uncertainty, we used recently published elasticity estimates from Her Majesty’s Revenue and Customs in the UK [36]. These estimates were derived using a very different methodology to that of Meng et al. [28] but use the same ten beverage categories. Full details of these sensitivity analyses can be found in S1 Appendix, Section 11.

## Results

### Baseline Consumption and Spending Patterns

As the four modelled policies target different beverages and different parts of the beverage price distributions, differential impacts of the modelled policies are partly explained by substantial subgroup variation in baseline alcohol consumption, baseline spending, and beverage category preferences (see Table 3). For example, minimum unit pricing impacts cheap high-strength alcohol found mainly in the off-trade sector, whereas, in absolute terms, ad valorem taxes affect expensive and on-trade products more.

**Table 3:** Baseline alcohol consumption and expenditure patterns by income quintile and drinking level, England 2014.

Population Distribution	All Drinkers	Moderate Drinkers					Increasing Risk Drinkers					Heavy Drinkers				
		Q1	Q2	Q3	Q4	Q5	Q1	Q2	Q3	Q4	Q5	Q1	Q2	Q3	Q4	Q5
**Number of drinkers (millions)**	36.3	4.6	5.5	5.8	5.9	5.4	0.9	1.0	1.4	1.6	2.1	0.4	0.3	0.4	0.4	0.5
**Percent of drinkers**	100.0%	12.8%	15.1%	16.1%	16.2%	14.9%	2.6%	2.7%	3.9%	4.5%	5.7%	1.2%	0.9%	1.1%	1.0%	1.4%
**Baseline consumption (UK alcohol units** ^a^ **)**																
Annual total	710	233	236	285	321	359	1,426	1,414	1,365	1,393	1,400	4,871	4,019	3,812	3,708	3,622
All off-trade^b^	458 (64.6%)	134 (57.5%)	128 (54.2%)	151 (53%)	161 (50.3%)	167 (46.6%)	884 (62%)	930 (65.7%)	899 (65.9%)	954 (68.5%)	947 (67.6%)	3,600 (73.9%)	2,953 (73.5%)	2,718 (71.3%)	2,784 (75.1%)	2,887 (79.7%)
All on-trade^c^	252 (35.4%)	99 (42.5%)	108 (45.8%)	134 (47%)	159 (49.7%)	192 (53.4%)	542 (38%)	485 (34.3%)	466 (34.1%)	439 (31.5%)	453 (32.4%)	1,271 (26.1%)	1,065 (26.5%)	1,094 (28.7%)	924 (24.9%)	735 (20.3%)
Off-trade beer	85 (11.9%)	33 (14.3%)	27 (11.5%)	28 (9.9%)	28 (8.7%)	25 (7%)	254 (17.8%)	212 (15%)	151 (11.1%)	147 (10.6%)	112 (8%)	902 (18.5%)	615 (15.3%)	628 (16.5%)	449 (12.1%)	366 (10.1%)
Off-trade cider	16 (2.2%)	6 (2.7%)	5 (2.1%)	4 (1.3%)	4 (1.3%)	3 (0.7%)	54 (3.8%)	33 (2.3%)	18 (1.3%)	15 (1.1%)	12 (0.9%)	332 (6.8%)	144 (3.6%)	122 (3.2%)	96 (2.6%)	53 (1.5%)
Off-trade wine	290 (40.8%)	63 (27.2%)	68 (28.8%)	93 (32.7%)	106 (32.9%)	116 (32.4%)	383 (26.8%)	523 (37%)	579 (42.4%)	679 (48.8%)	686 (49%)	1,836 (37.7%)	1,824 (45.4%)	1,539 (40.4%)	1,926 (52%)	2,269 (62.6%)
Off-trade spirits	68 (9.5%)	31 (13.3%)	28 (11.8%)	26 (9.2%)	24 (7.4%)	23 (6.5%)	193 (13.5%)	162 (11.4%)	151 (11.1%)	112 (8%)	136 (9.7%)	529 (10.9%)	370 (9.2%)	429 (11.3%)	313 (8.4%)	200 (5.5%)
On-trade beer	175 (24.6%)	65 (27.9%)	72 (30.7%)	89 (31.1%)	99 (30.9%)	100 (27.8%)	433 (30.3%)	385 (27.2%)	344 (25.2%)	314 (22.6%)	288 (20.6%)	995 (20.4%)	871 (21.7%)	956 (25.1%)	718 (19.4%)	547 (15.1%)
On-trade cider	7 (0.9%)	3 (1.1%)	3 (1.3%)	3 (1.1%)	5 (1.5%)	3 (0.9%)	20 (1.4%)	14 (1%)	12 (0.9%)	12 (0.8%)	7 (0.5%)	35 (0.7%)	41 (1%)	33 (0.9%)	46 (1.3%)	15 (0.4%)
On-trade wine	43 (6.1%)	11 (4.5%)	14 (6%)	25 (8.8%)	34 (10.6%)	63 (17.5%)	25 (1.8%)	51 (3.6%)	57 (4.2%)	82 (5.9%)	115 (8.2%)	153 (3.1%)	83 (2.1%)	61 (1.6%)	98 (2.6%)	147 (4.1%)
On-trade spirits	27 (3.8%)	21 (8.9%)	18 (7.8%)	17 (6%)	21 (6.7%)	26 (7.2%)	64 (4.5%)	34 (2.4%)	53 (3.9%)	31 (2.2%)	43 (3.1%)	89 (1.8%)	71 (1.8%)	44 (1.2%)	62 (1.7%)	26 (0.7%)
**Baseline spending** ^d^																
Annual spending	£644	£228	£239	£300	£364	£465	£1,169	£1,152	£1,176	£1,204	£1,356	£3,060	£2,881	£2,851	£2,908	£2,942
All off-trade	£261 (40.6%)	£73 (31.9%)	£72 (30.2%)	£89 (29.8%)	£102 (27.9%)	£115 (24.7%)	£443 (37.9%)	£486 (42.2%)	£494 (42%)	£554 (46%)	£621 (45.8%)	£1,589 (51.9%)	£1,466 (50.9%)	£1,409 (49.4%)	£1,562 (53.7%)	£1,862 (63.3%)
All on-trade	£382 (59.4%)	£155 (68.1%)	£167 (69.8%)	£210 (70.2%)	£263 (72.1%)	£350 (75.3%)	£726 (62.1%)	£665 (57.8%)	£682 (58%)	£650 (54%)	£736 (54.2%)	£1,471 (48.1%)	£1,415 (49.1%)	£1,442 (50.6%)	£1,345 (46.3%)	£1,080 (36.7%)
Off-trade beer	£41 (6.3%)	£17 (7.4%)	£14 (5.7%)	£15 (4.9%)	£15 (4.2%)	£14 (3.1%)	£116 (9.9%)	£99 (8.6%)	£71 (6.1%)	£74 (6.1%)	£58 (4.2%)	£392 (12.8%)	£262 (9.1%)	£269 (9.4%)	£206 (7.1%)	£181 (6.1%)
Off-trade cider	£6 (1%)	£3 (1.2%)	£2 (1%)	£2 (0.6%)	£2 (0.6%)	£1 (0.3%)	£20 (1.7%)	£14 (1.2%)	£8 (0.7%)	£7 (0.6%)	£7 (0.5%)	£111 (3.6%)	£50 (1.7%)	£49 (1.7%)	£40 (1.4%)	£21 (0.7%)
Off-trade wine	£175 (27.3%)	£34 (15.1%)	£39 (16.4%)	£56 (18.7%)	£70 (19.2%)	£85 (18.3%)	£201 (17.2%)	£288 (25%)	£331 (28.1%)	£410 (34%)	£472 (34.8%)	£794 (25.9%)	£968 (33.6%)	£861 (30.2%)	£1149 (39.5%)	£1,555 (52.8%)
Off-trade spirits	£39 (6%)	£19 (8.2%)	£17 (7.1%)	£17 (5.6%)	£15 (4%)	£14 (3.1%)	£105 (9%)	£85 (7.4%)	£84 (7.1%)	£64 (5.3%)	£85 (6.3%)	£292 (9.5%)	£186 (6.4%)	£230 (8.1%)	£168 (5.8%)	£105 (3.6%)
On-trade beer	£236 (36.7%)	£86 (37.8%)	£97 (40.7%)	£123 (41%)	£145 (39.7%)	£154 (33.2%)	£533 (45.6%)	£493 (42.8%)	£451 (38.4%)	£428 (35.6%)	£414 (30.5%)	£1,178 (38.5%)	£1,097 (38.1%)	£1,212 (42.5%)	£965 (33.2%)	£740 (25.2%)
On-trade cider	£9 (1.4%)	£3 (1.5%)	£4 (1.7%)	£4 (1.4%)	£7 (1.8%)	£5 (1.1%)	£25 (2.1%)	£17 (1.5%)	£17 (1.4%)	£16 (1.3%)	£10 (0.7%)	£41 (1.3%)	£52 (1.8%)	£39 (1.4%)	£63 (2.2%)	£20 (0.7%)
On-trade wine	£65 (10.2%)	£14 (6.2%)	£20 (8.3%)	£36 (12.1%)	£52 (14.2%)	£110 (23.6%)	£33 (2.9%)	£70 (6.1%)	£81 (6.9%)	£124 (10.3%)	£189 (13.9%)	£70 (2.3%)	£114 (4%)	£84 (2.9%)	£153 (5.3%)	£247 (8.4%)
On-trade spirits	£72 (11.1%)	£51 (22.6%)	£46 (19.2%)	£47 (15.7%)	£60 (16.4%)	£81 (17.3%)	£134 (11.5%)	£85 (7.4%)	£133 (11.3%)	£82 (6.8%)	£123 (9.1%)	£181 (5.9%)	£151 (5.2%)	£107 (3.8%)	£164 (5.6%)	£73 (2.5%)

Income quintiles (from low, Q1, to high, Q5) are calculated based on the total population including abstainers. A higher proportion of those in the lowest income quintile are abstainers than those in the higher income quintiles.

^a^1 UK alcohol unit = 8 g of pure ethanol.

^b^Off-trade alcohol refers to alcohol bought in shops such as off-licenses and supermarkets.

^c^On-trade alcohol refers to alcohol bought for consumption in drinking venues such as pubs, bars, clubs, and restaurants.

^d^Prices are 2014 prices.

The relationship between income and mean consumption differs by drinking level. Whilst the proportion of heavy drinkers in the highest income group (6%) is larger than in the lowest income group (4.9%), heavy drinkers in the lowest income quintile have a higher mean consumption than heavy drinkers in the highest income quintile (4,871 versus 3,622 units per year). In contrast, among moderate drinkers, there is a positive relationship between drinking level and income, whereby higher incomes are associated with higher mean consumption.

In terms of spending, the lowest income moderate drinker group spends the least (£228 per year; less than 1% of gross equivalised household income), while heavy drinkers spend the most irrespective of income (approximately £3,000 per year, which is almost 10% of mean gross equivalised income in the lowest income quintile). Low-income heavy drinkers spend a similar amount to high-income heavy drinkers, but consume a third more because they pay substantially less per unit of alcohol (£0.63/unit on average versus £0.81/unit).

**Fig 1:**
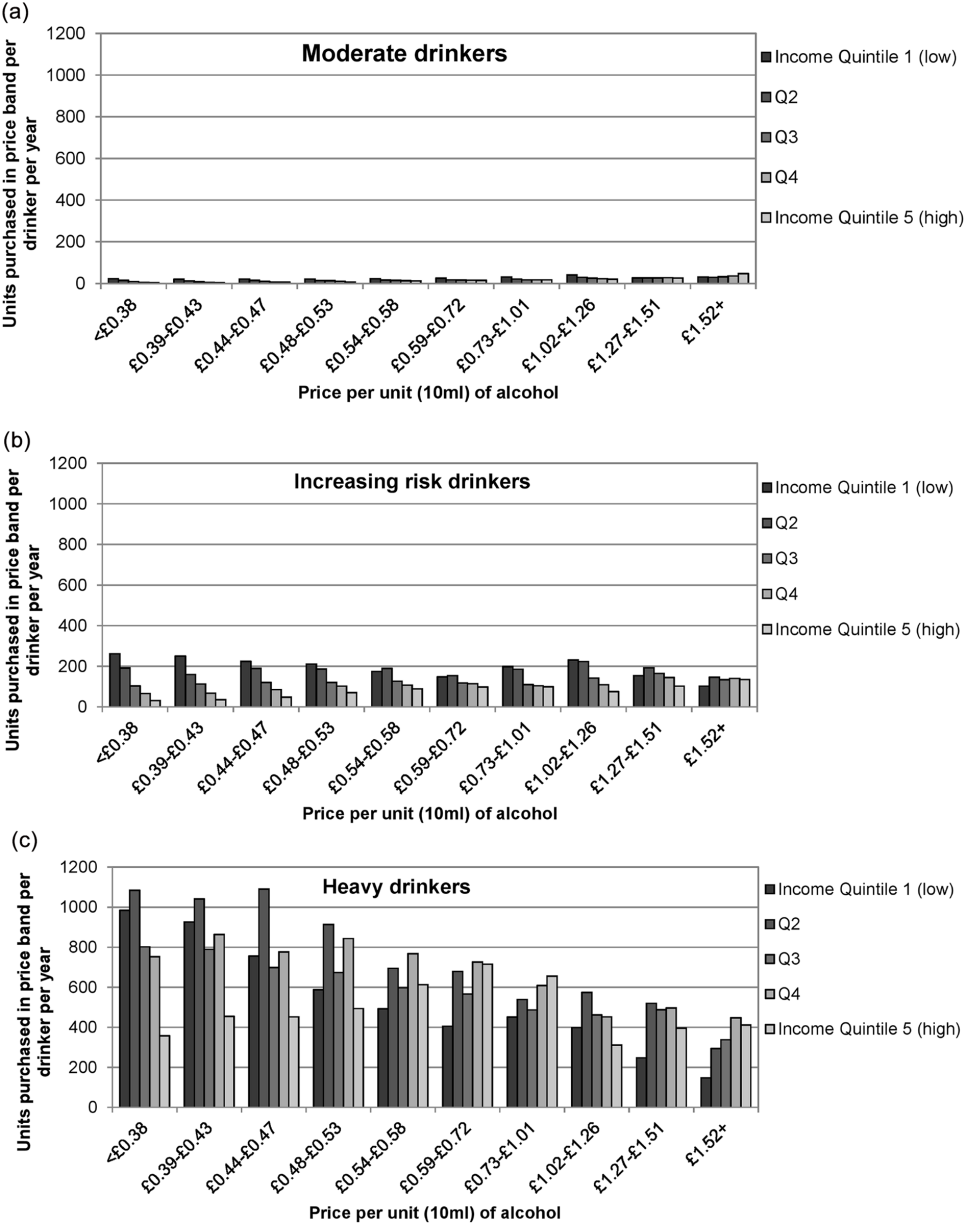
Distribution of alcohol purchasing by income. This figure shows the average number of units purchased per moderate drinker per year for alcohol across price bands, stratified by income. Moderate drinkers (a) purchase little alcohol overall and show a preference for middle and higher price bands, even when having lower incomes. Increasing risk drinkers (b) purchase alcohol across all price bands, but there is a clear gradient whereby poorer drinkers purchase more of the cheapest alcohol. Heavy drinkers (c)—across income quintiles, but especially poorer heavy drinkers—show a very clear preference for cheaper alcohol. Price bands are deciles of the price distribution of all alcohol sold in England. Income groups are defined by equivalised income quintiles.

Fig 1 shows ten price bands (deciles of the price distribution of all alcohol) and the number of units purchased in each band by each population subgroup. Moderate drinkers, even in the lowest income group, purchase very little of the cheapest alcohol. In contrast, both increasing risk and heavy drinkers buy substantially more cheap alcohol than expensive alcohol, and this is partly due to their strong preference for off-trade alcohol (cf. Table 4). Only the highest income group of heavy drinkers spreads their spending across cheap, mid-price, and expensive alcohol.

**Table 4:** Policy effects on alcohol consumption and consumer spending, by drinking level and income group.

Drinker Group	Income Quintile^a^	Annual Alcohol Consumption					Annual Consumer Spending				
		Baseline Alcohol Units per Year	Change in Units (Percent)				Baseline GBP per Year	Change in GBP (Percent)			
			Current Tax Increase	Ad Valorem Tax	Volumetric Tax	Minimum Unit Pricing		Current Tax Increase	Ad Valorem Tax	Volumetric Tax	Minimum Unit Pricing
**Consumption level**											
All drinkers		710	−12 (−1.7%)	−13 (−1.8%)	−13 (−1.9%)	−13 (−1.8%)	644	20 (3.1%)	14 (2.2%)	−5 (−0.8%)	11 (1.7%)
Moderate drinkers		289	−4 (−1.5%)	−5 (−1.7%)	−4 (−1.4%)	−3 (−0.9%)	322	8 (2.6%)	7 (2.3%)	−1 (−0.3%)	2 (0.7%)
Increasing risk drinkers		1,397	−24 (−1.7%)	−25 (−1.8%)	−21 (−1.5%)	−16 (−1.2%)	1,231	41 (3.3%)	27 (2.2%)	−11 (−0.9%)	23 (1.9%)
Heavy drinkers		3,998	−78 (−1.9%)	−75 (−1.9%)	−110 (−2.8%)	−134 (−3.3%)	2,933	106 (3.6%)	170 (2.1%)	−99 (−1.4%)	81 (2.8%)
**Consumption level × income quintile**											
All drinkers	Q1 (low)	748	−17 (−2.3%)	−17 (−2.2%)	−36 (−4.9%)	−43 (−5.8%)	575	16 (2.8%)	11 (1.9%)	−18 (−3.1%)	3 (0.6%)
	Q2	582	−12 (−2.1%)	−13 (−2.2%)	−16 (−2.7%)	−17 (−2.8%)	494	14 (2.9%)	10 (2.0%)	−9 (−1.7%)	8 (1.7%)
	Q3	665	−13 (−2.0%)	−14 (−2.0%)	−13 (−2.0%)	−12 (−1.9%)	592	17 (2.9%)	12 (2.0%)	−8 (−1.3%)	10 (1.7%)
	Q4	701	−11 (−1.6%)	−12 (−1.7%)	−7 (−1.0%)	−2 (−0.3%)	657	21 (3.1%)	15 (2.2%)	−2 (−0.3%)	13 (2.0%)
	Q5 (high)	841	−9 (−1.1%)	−10 (−1.2%)	1 (0.1%)	3 (0.4%)	857	29 (3.4%)	22 (2.5%)	6 (0.7%)	17 (2.0%)
Moderate drinkers	Q1 (low)	233	−5 (−2.2%)	−5 (−2.1%)	−7 (−3.1%)	−7 (−2.9%)	228	5 (2.4%)	5 (2.1%)	−5 (−2.0%)	0 (0.0%)
	Q2	236	−5 (−1.9%)	−5 (−2.0%)	−6 (−2.5%)	−4 (−1.8%)	239	6 (2.5%)	5 (2.1%)	−4 (−1.6%)	2 (0.6%)
	Q3	285	−5 (−1.8%)	−5 (−1.9%)	−4 (−1.4%)	−2 (−0.8%)	300	7 (2.5%)	6 (2.1%)	−2 (−0.6%)	3 (0.9%)
	Q4	321	−4 (−1.4%)	−5 (−1.6%)	−4 (−1.1%)	−1 (−0.2%)	364	9 (2.6%)	8 (2.2%)	0 (−0.1%)	4 (1.0%)
	Q5 (high)	359	−3 (−0.9%)	−4 (−1.1%)	0 (0.0%)	0 (0.1%)	465	13 (2.7%)	12 (2.6%)	5 (1.0%)	4 (0.8%)
Increasing risk drinkers	Q1 (low)	1,426	−36 (−2.5%)	−36 (−2.6%)	−69 (−4.9%)	−73 (−5.1%)	1,169	32 (2.7%)	21 (1.8%)	−42 (−3.6%)	8 (0.7%)
	Q2	1,414	−32 (−2.3%)	−32 (−2.3%)	−38 (−2.7%)	−37 (−2.6%)	1,152	33 (2.9%)	20 (1.7%)	−21 (−1.8%)	18 (1.6%)
	Q3	1,365	−25 (−1.9%)	−26 (−1.9%)	−21 (−1.5%)	−15 (−1.1%)	1,176	39 (3.3%)	25 (2.2%)	−16 (−1.4%)	25 (2.1%)
	Q4	1,393	−22 (−1.6%)	−23 (−1.7%)	−9 (−0.6%)	−1 (−0.1%)	1,204	42 (3.5%)	27 (2.3%)	−2 (−0.2%)	28 (2.3%)
	Q5 (high)	1,400	−16 (−1.2%)	−18 (−1.3%)	0 (0.0%)	7 (0.5%)	1,356	49 (3.6%)	34 (2.5%)	4 (0.3%)	28 (2.0%)
Heavy drinkers	Q1 (low)	4,871	−107 (−2.2%)	−101 (−2.1%)	−282 (−5.8%)	−372 (−7.6%)	3,060	99 (3.2%)	57 (1.9%)	−113 (−3.7%)	28 (0.9%)
	Q2	4,019	−89 (−2.2%)	−86 (−2.1%)	−122 (−3.0%)	−165 (−4.1%)	2,881	105 (3.6%)	60 (2.1%)	−53 (−1.9%)	94 (3.3%)
	Q3	3,812	−93 (−2.4%)	−89 (−2.3%)	−125 (−3.3%)	−151 (−4.0%)	2,851	89 (3.1%)	46 (1.6%)	−67 (−2.4%)	62 (2.2%)
	Q4	3,708	−64 (−1.7%)	−65 (−1.8%)	−53 (−1.4%)	−24 (−0.7%)	2,908	109 (3.7%)	62 (2.1%)	−19 (−0.7%)	100 (3.5%)
	Q5 (high)	3,622	−46 (−1.3%)	−45 (−1.2%)	7 (0.2%)	15 (0.4%)	2,942	123 (4.2%)	74 (2.5%)	29 (1.0%)	117 (4.0%)

^a^Income quintiles (Q1 to Q5) are calculated based on the total population including abstainers. A higher proportion of those in the lowest income quintile are abstainers than those in the higher income quintiles.

GBP, British pound.

### Policy Effects on Alcohol Consumption and Consumer Spending

Table 4 shows the policies’ estimated effects on average alcohol consumption and spending by drinking level for the whole population and by income quintile. This table contains the deterministic results, which represent our best estimate of policy effects. Equivalent results from the partial PSA, including 95% uncertainty intervals (UIs), can be found in the tables in S1 Appendix, Sections 12.8–12.11.

Population-level consumption reductions are similar for all four modelled tax policies (ranging from −1.7% to −1.9%). When examined by drinking level, volumetric tax and minimum unit pricing lead to the largest estimated consumption reductions among heavy drinkers (current tax increase: −1.9% [mean PSA result (PSA) −2.3% (95% UI −4.5%, 0.0%)]; ad valorem tax: −1.9% [PSA −2.2% (UI −3.4%, −0.8%)]; volumetric tax: −2.8% [PSA −3.7% (UI −7.7%, −1.1%)]; minimum unit pricing: −3.3% [PSA −5.0% (UI −8.9%, −2.4%)]) and the smallest reductions among moderate drinkers (current tax increase: −1.5% [PSA −1.9% (UI −3.4%, −0.2%)]; ad valorem tax: −1.7% [PSA −1.9% (UI −3.0%, −0.4%)]; volumetric tax: −1.4% [PSA −2.1% (UI −5.3%, +0.1%)]; minimum unit pricing: −0.9% [PSA −1.5% (UI −2.9%, −0.3%)]). In absolute terms, a 1.5% consumption reduction is just four units per year for the average moderate drinker, but a 1.9% reduction is 76 units for the average heavy drinker.

Responses to the different tax policies also vary by income group. Volumetric tax and minimum unit pricing have the steepest income gradients for consumption effects, i.e., larger reductions in low-income groups and smaller reductions in high-income groups. This income gradient for minimum unit pricing and volumetric taxation is steeper for heavy drinkers (in both relative and absolute terms) than for moderate drinkers. A shallower income gradient is seen for an increase of the current tax and ad valorem tax, with little variation in the gradient by consumption group.

In terms of the consumer spending impact across income groups, we estimated that all four policies would produce greater increases in spending on alcohol for higher earners. An increase of the current tax would lead to the highest expenditure increases overall (+3.1% [PSA 2.8% (UI 1.3%, 4.6%)]), with these being largest among heavy drinkers (+3.6% [PSA 3.3% (UI 1.4%, 5.2%)], or £106 per year in absolute terms) and smaller for moderate drinkers (+2.6% [PSA 2.3% (UI 0.8%, 4.2%)], or £8 per year). The ad valorem tax would prompt smaller changes in expenditure (+2.2% overall [PSA 2.0% (UI 1.0%, 3.6%)]), with little subgroup variation. A volumetric tax was estimated to slightly reduce spending overall (−0.8% [PSA −1.5% (UI −4.7%, +1.0%)]), with declines in all but the highest income group. Further analyses show that although the tax on beer and cider would increase under a volumetric tax of £0.22 per unit, the tax on spirits would decrease, and, together with switching behaviour, this leads to an overall reduction in spending. Minimum unit pricing would have a smaller impact on spending than increasing the current tax or adding an ad valorem tax (+1.7% overall [PSA 1.0% (UI −0.9%, +3.0%)]).

Considering consumption and spending together allows examination of how different groups would balance decreasing their consumption versus increasing their spending. For example, under minimum unit pricing, heavy drinkers in the poorest income quintile would reduce their consumption (−7.6% [PSA −9.8% (UI −14.8%, −5.6%)]) while maintaining their expenditure on alcohol (+0.9% [PSA 0.0% (UI −4.0%, +4.2%)]), whereas those in the richest income quintile would maintain their drinking (+0.4% [PSA −0.5% (UI −4.3%, +3.1%)]) but increase their expenditure (+4.0% [PSA 3.3% (UI 0.2%, 6.4%)]).

### Policy Effects on Alcohol-Attributable Mortality

Table 5 shows each policy’s estimated impact on annual alcohol-related mortality by socioeconomic (occupation) group. As policy “strength” (e.g., the size of the tax increase) is calibrated to result in a modelled 4.3% total alcohol-related mortality reduction, our analysis allows for straightforward examination of differential effects across population groups.

**Table 5:** Annual full effects of policies on alcohol-related deaths, by drinking level and socioeconomic group.

Drinking Level Group	Occupation Group	Percent of the Total Drinker Population	Deaths per Year at Baseline^a^	Percent Change in Number of Deaths per Year^b^				Absolute Change in Number of Deaths per Year			
				Current Tax Increase	Ad Valorem Tax	Volumetric Tax	Minimum Unit Pricing	Current Tax Increase	Ad Valorem Tax	Volumetric Tax	Minimum Unit Pricing
**Total drinker population**		100.0%	12,190	−4.3%	−4.3%	−4.3%	−4.3%	−529	−530	−530	−530
**All drinkers**	Routine/manual	36.0%	6,223	−4.9%	−4.8%	−8.1%	−8.9%	−306	−296	−502	−551
	Intermediate	24.5%	2,684	−4.3%	−4.3%	−2.5%	−2.2%	−115	−116	−66	−58
	Professional/managerial	38.8%	3,283	−3.3%	−3.6%	1.2%	2.4%	−108	−117	38	79
**Moderate**	Routine/manual	28.0%	−691	*3*.*4%*	*3*.*6%*	*4*.*5%*	*2*.*5%*	−24	−25	−31	−17
	Intermediate	17.9%	−855	*0*.*9%*	*1*.*0%*	*0*.*6%*	*0*.*3%*	−8	−9	−5	−3
	Professional/managerial	28.6%	−1,331	*0*.*6%*	*0*.*8%*	*0*.*2%*	*0*.*0%*	−8	−10	−3	0
**Increasing risk**	Routine/manual	5.9%	2,260	−5.9%	−6.0%	−8.3%	−7.6%	−134	−135	−188	−172
	Intermediate	5.1%	1,175	−5.0%	−5.2%	−2.1%	−1.1%	−59	−61	−24	−13
	Professional/managerial	8.3%	1,556	−3.8%	−4.1%	2.3%	3.5%	−59	−64	36	54
**Heavy**	Routine/manual	2.1%	4,654	−3.2%	−2.9%	−6.1%	−7.8%	−148	−137	−283	−362
	Intermediate	1.5%	2,363	−2.1%	−2.0%	−1.6%	−1.8%	−49	−47	−37	−42
	Professional/managerial	1.9%	3,057	−1.3%	−1.4%	0.2%	0.8%	−41	−43	5	26

“Full effects” refers to annual effects in the 20th year following policy implementation.

^a^Net alcohol-attributable deaths are the sum of excess deaths and prevented deaths attributable to alcohol. In this column, negative numbers indicate that in the absence of alcohol there would be more deaths; positive numbers indicate that without alcohol there would be fewer deaths.

^b^Percentage changes in italics represent overall decreases in deaths (they are a reduction from a negative deaths baseline).

The policy impacts on moderate drinkers’ deaths per year are small across policies and socioeconomic groups. In contrast, for heavy drinkers, there are large differences in how mortality gains are distributed across socioeconomic groups with different policies. Volumetric tax and minimum unit pricing in particular are estimated to substantially reduce mortality among heavy drinkers in the routine/manual occupation group (current tax increase: −3.2% [PSA −3.6% (UI −6.1%, −0.6%)]; ad valorem tax: −2.9% [PSA −3.3% (UI −5.1%, −1.7%)]; volumetric tax: −6.1% [PSA −7.5% (UI −13.7%, −3.9%)]; minimum unit pricing: −7.8% [PSA −10.3% (UI −16.7%, −7.0%)]). There is also substantial variation in impact in the professional/managerial occupation group (current tax increase: −1.3% [PSA −1.8% (UI −4.7%, +1.6%)]; ad valorem tax: −1.4% [PSA −1.9% (UI −3.6%, +0.4%)]; volumetric tax: +0.2% [PSA −0.8% (UI −6.9%, +4.0%)]; minimum unit pricing: +0.8% [PSA −0.7% (UI −5.6%, +3.6%)]).

**Fig 2:**
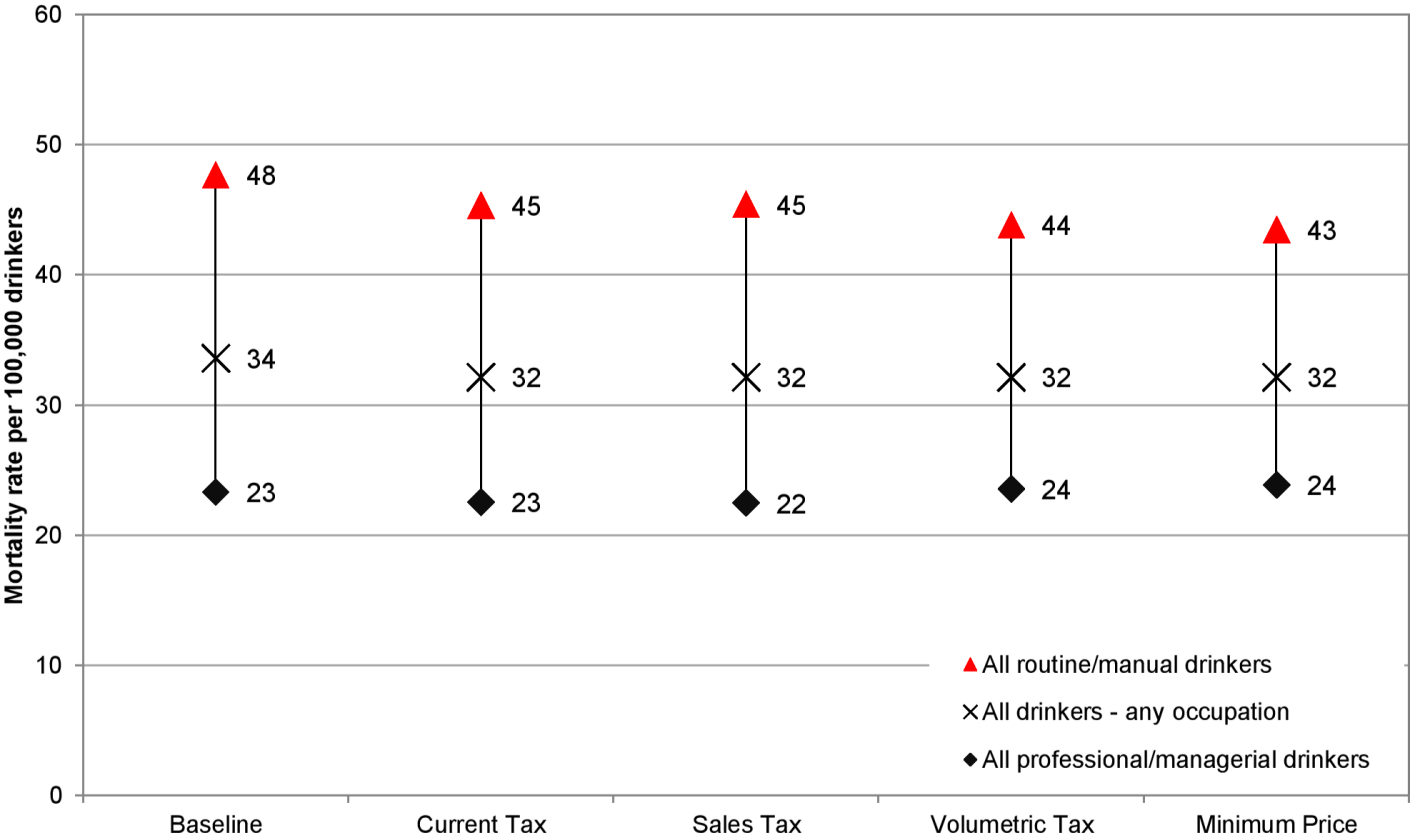
Equity gap in the alcohol-related death rates between those in routine/manual and professional/managerial occupations: total drinker population. This figure shows the effect of alcohol pricing policies on the gap between alcohol-related death rates for drinkers in the highest and lowest socioeconomic occupation groups. All policies reduce this gap, but volumetric tax and minimum unit pricing (“Minimum Price”) have larger effects on the gap than ad valorem tax (“Sales Tax”) or increasing the UK’s current tax (“Current Tax”).

**Fig 3:**
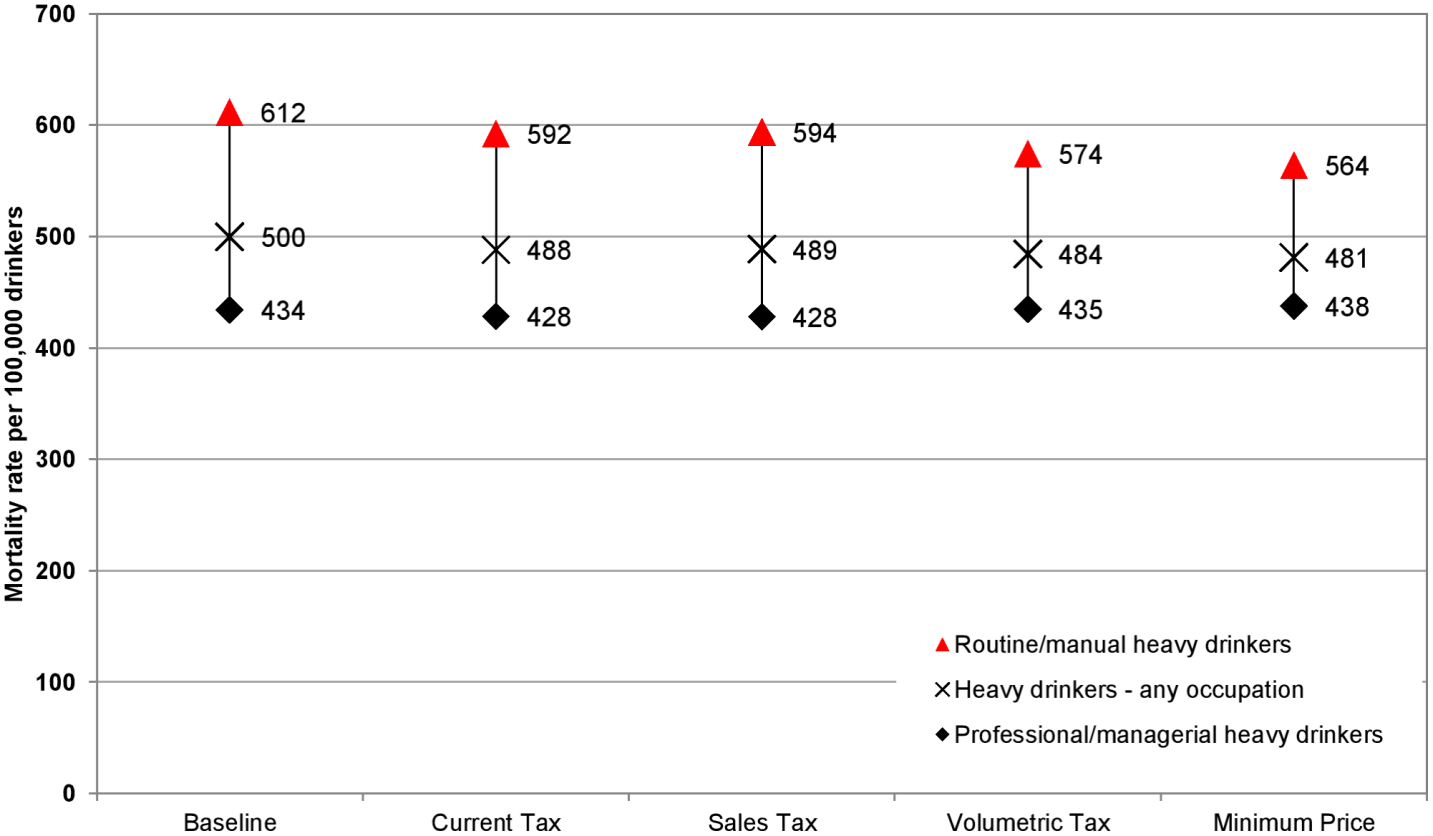
Equity gap in the alcohol-related death rates between those in routine/manual and professional/managerial occupations: heavy drinkers. This figure shows the effect of alcohol pricing policies on the gap between alcohol-related death rates for heavy drinkers in the highest and lowest socioeconomic occupation groups. All policies reduce this gap, but volumetric tax and minimum unit pricing (“Minimum Price”) have larger effects on the gap than ad valorem tax (“Sales Tax”) or increasing the UK’s current tax (“Current Tax”).

These results translate to differing impacts on health inequalities. Baseline alcohol-related mortality rates are almost twice as high in the routine/manual occupation group compared to the professional/managerial occupation group. All policies narrow health inequalities, but to varying degrees. Figs 2 and 3 illustrate the estimated policy effects on health inequalities for all drinkers and for heavy drinkers alone. Minimum unit pricing narrows the gap between socioeconomic groups the most, by considerably reducing the mortality rate for heavy drinkers in the routine/manual occupation group and by slightly increasing the mortality rate for individuals in the professional/managerial occupation group. Volumetric taxation similarly achieves large health inequality reductions, whereas increasing the current tax or adding an ad valorem tax has more modest effects on health inequalities.

### Sensitivity Analysis

**Fig 4:**
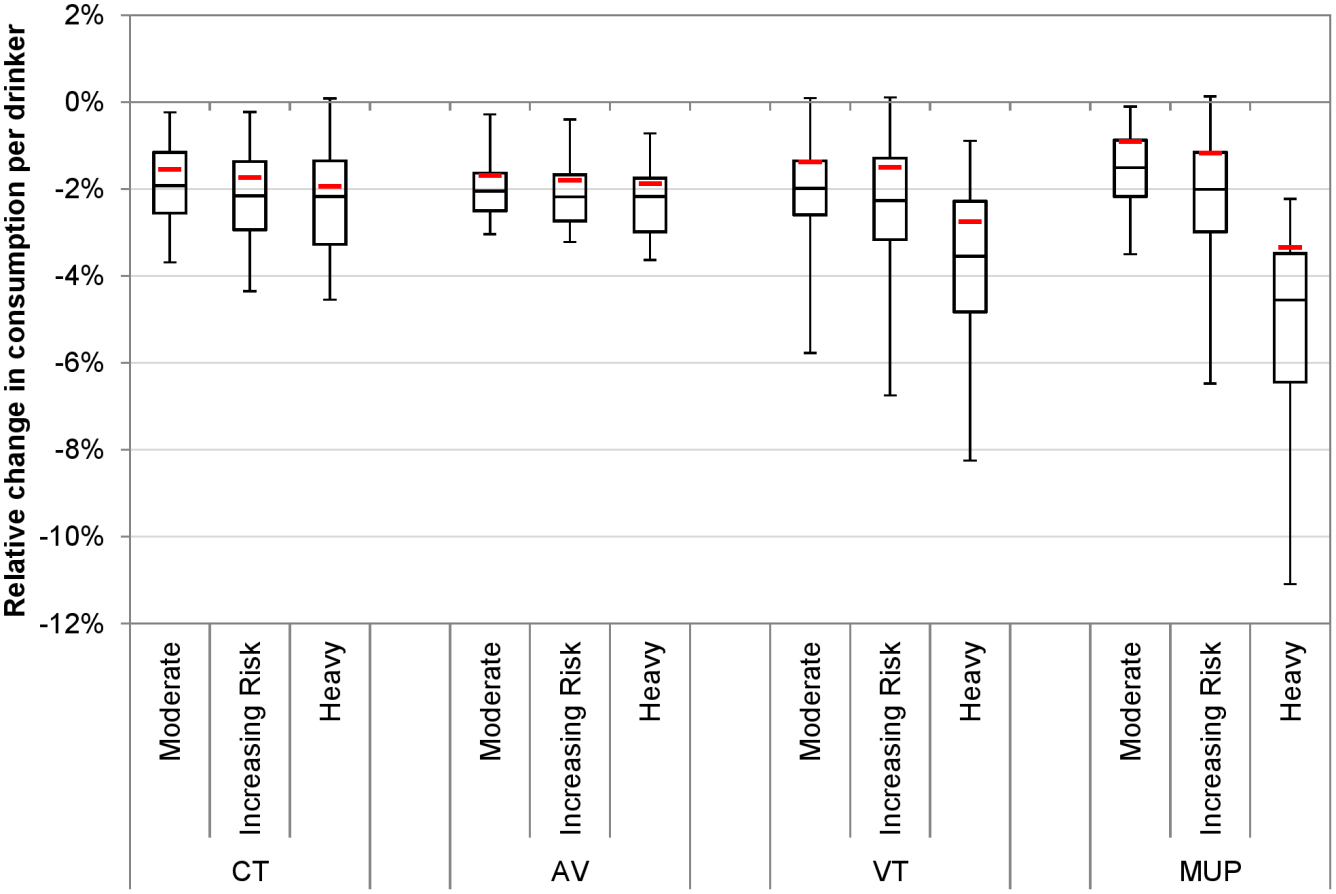
Probabilistic sensitivity analysis results for policy impacts on consumption by drinking level group. This figure shows the uncertainty in estimated policy impacts on alcohol consumption by drinking level group generated from 30 PSA runs. Whiskers represent maximum and minimum values. Boxes represent interquartile ranges. Mid-box lines represent medians. Red lines represent the baseline (deterministic) results. AV, ad valorem tax; CT, current tax increase; MUP, minimum unit pricing; VT, volumetric tax.

PSA results for policy impacts on alcohol consumption by drinking level group are presented visually in Fig 4. These results display two interesting features. The first is that the central, deterministic estimate of effect is always smaller than the median PSA estimate, particularly for the volumetric taxation and minimum unit pricing policies. This suggests that the central estimates are likely to be conservative and also that values on the more pessimistic (in terms of the scale of achievable effects of tax policies) end of the uncertainty spectrum affect the results by only a small amount, but those on the more optimistic end of the spectrum lead to substantially larger estimates of effect. The second feature is that, across all four modelled policies, the magnitude of the uncertainty in effect differs for heavy drinkers versus moderate and increasing risk drinkers. The magnitude of the uncertainty for moderate and increasing risk drinkers is substantial in comparison to the estimated size of the effect. For heavy drinkers, however, the results show that it is highly likely that minimum unit pricing and volumetric tax would reduce health inequalities more than a rise in current taxation or introduction of an ad valorem tax. The patterns of effects seen in the deterministic results are reflected in the PSA results. In particular, the minimum unit pricing and volumetric tax policies show greater differences between the impacts on moderate drinkers and heavy drinkers than are seen for the current tax increase and ad valorem tax policies.

**Fig 5:**
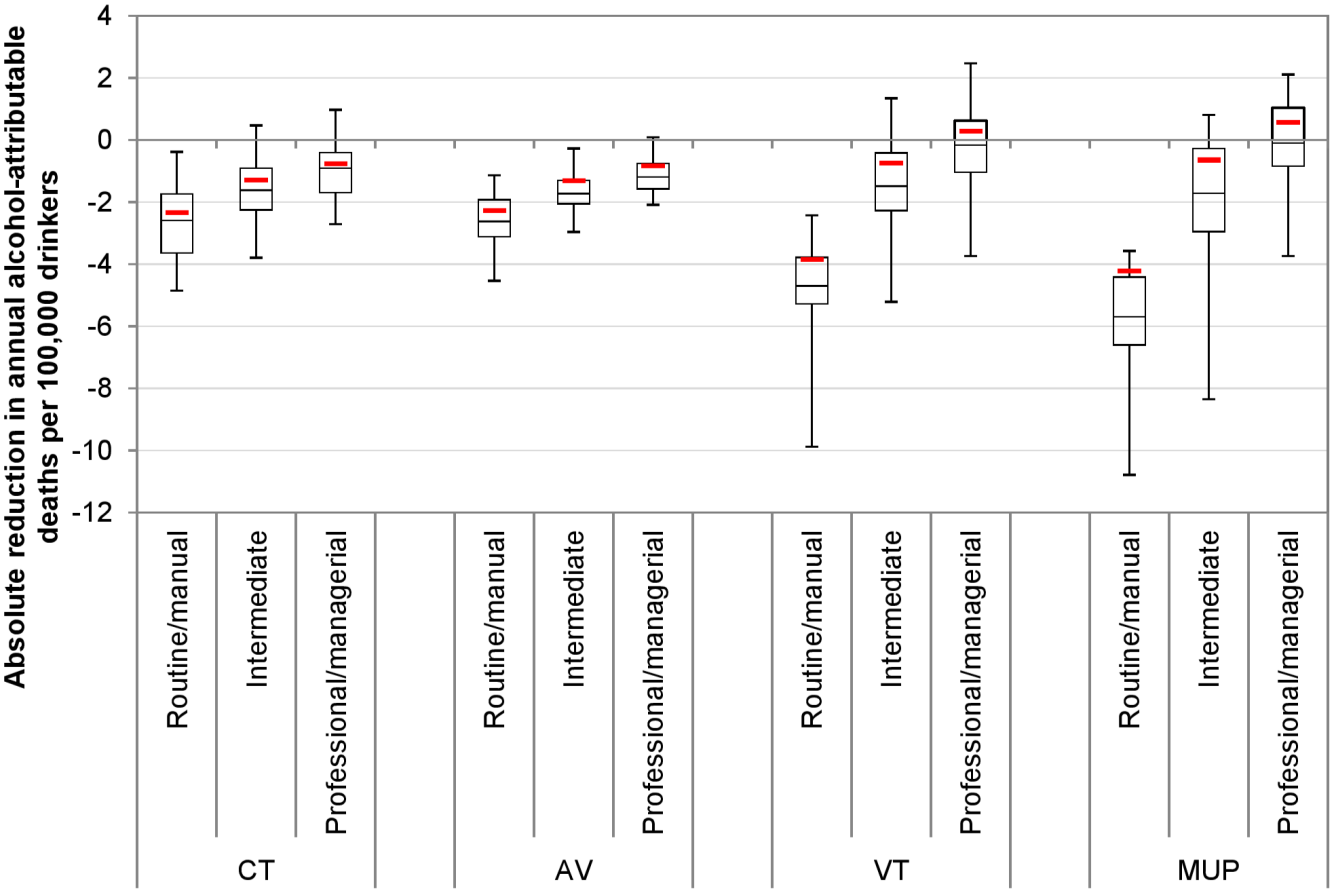
Probabilistic sensitivity analysis results for policy impacts on deaths by socioeconomic group. This figure shows the uncertainty in estimated policy impacts on death rates by socioeconomic group generated from 30 PSA runs. Whiskers represent maximum and minimum values. Boxes represent interquartile ranges. Mid-box lines represent medians. Red lines represent the baseline (deterministic) results. AV, ad valorem tax; CT, current tax increase; MUP, minimum unit pricing; VT, volumetric tax.

Fig 5 illustrates the PSA results for policy effects on death rates, by socioeconomic group. These results again suggest that the deterministic model estimates above are conservative, particularly for the volumetric taxation and minimum unit pricing policies. Whilst the deterministic model suggests that a volumetric tax or minimum unit pricing would increase deaths in the highest socioeconomic group, the median PSA estimate suggests that deaths would decrease marginally. Also of note is the fact that, while the uncertainty is greatest around these two policies, their impact on deaths in lower socioeconomic groups is clearly greater than that of the current tax increase and ad valorem tax policies.

**Fig 6:**
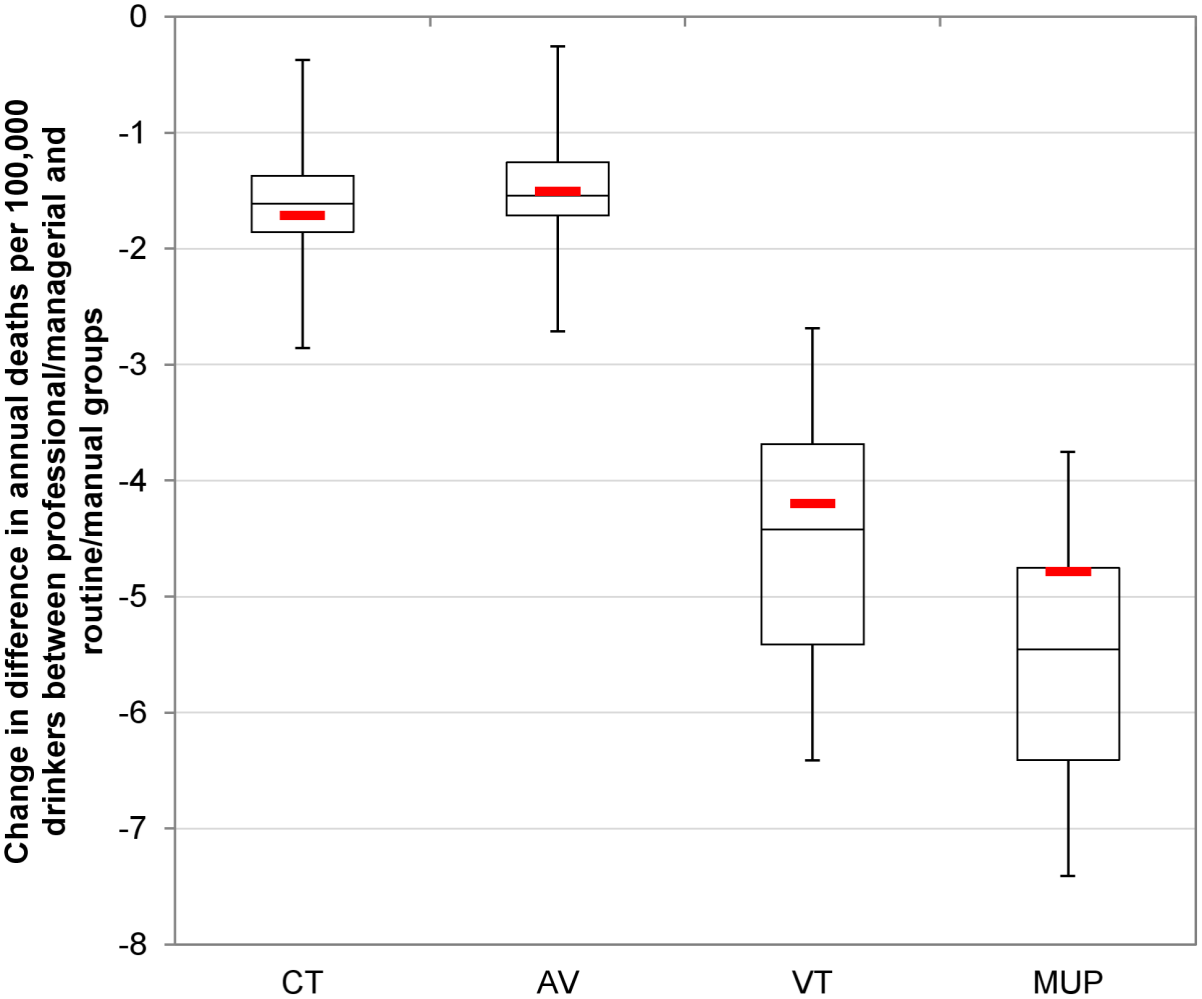
Probabilistic sensitivity analysis results for policy impacts on health inequalities. This figure shows PSA results for policy impacts on the equity gap in alcohol-related death rates between the routine/manual and professional/managerial occupation groups. The overall plot represents uncertainty generated from 30 PSA runs. Whiskers represent maximum and minimum values. Boxes represent interquartile ranges. Mid-box lines represent medians. Red lines represent the baseline (deterministic) results. AV, ad valorem tax; CT, current tax increase; MUP, minimum unit pricing; VT, volumetric tax.

Fig 6—like Figs 2 and 3 for the deterministic results—shows the PSA results for the reduction in the equity gap between the routine/manual and professional/managerial occupation groups. In particular, the marked difference in impact by socioeconomic group for volumetric taxation and, to a greater extent, minimum unit pricing means that the uncertainty around the greater inequality-reducing effect of these policies relative to other options is minimal.

Overall, the PSA results suggest that the impact of uncertainty on model results is substantial, particularly around the point estimates of policy effectiveness, but this uncertainty has few implications for the main conclusions of the analysis. This is because there is considerably less uncertainty around the relative effectiveness of the four policies, with volumetric taxation and minimum unit pricing consistently outperforming an increase of the current tax and ad valorem taxation, both in terms of specifically targeting the heaviest drinkers and in terms of reducing alcohol-related health inequalities. For example, while the most optimistic estimate of the impact of a rise in current tax exceeds the most pessimistic estimate of the impact of minimum unit pricing, the relative ranking of the policies is largely maintained across the 30 PSA samples. In 26 out of 30 PSA runs, volumetric taxation leads to a greater reduction in deaths in the routine/manual occupation group than an increase in the current tax, while minimum unit pricing exceeds volumetric tax on this outcome in 21 out of 30 model runs and exceeds an increase in the current tax in 30 out of 30 runs. Full PSA results are presented in S1 Appendix, Section 12.

Results for the additional deterministic sensitivity analysis using alternative elasticities derived by Her Majesty’s Revenue and Customs are presented in the table in S1 Appendix, Section 11.3, and show that using this alternative input makes little difference to the overall conclusions. Overall reductions in consumption are, however, estimated to be somewhat greater for all policies, particularly for minimum unit pricing.

## Discussion

To our knowledge, this study is the first to estimate how four key alcohol taxation and price control systems compare in their targeting of key population subgroups and reducing health disparities between socioeconomic groups. While all policies were estimated to reduce health inequalities because drinking is associated with substantially higher absolute health risks in lower socioeconomic groups than in higher socioeconomic groups [37], the scale of the inequality reduction varied across the policies. A £0.50 minimum unit price and a £0.22 per unit volumetric tax were estimated to reduce inequalities the most because heavy drinkers in lower socioeconomic groups buy proportionately more of the cheap alcohol most affected by these policies. Estimated impacts on health inequalities were smaller for a 4.0% alcohol ad valorem tax and a 13.4% current duty increase as price increases were more evenly distributed across the alcohol consumed by different socioeconomic groups.

Our study incorporates evidence of differential pass through of alcohol taxes across products and prices [26] and builds on our previous modelling of the equity implications of minimum unit pricing [14] by incorporating an improved model of the relationship between mean weekly alcohol consumption and occasion-specific drinking [6]. Study limitations include reducing model complexity by not considering a newly introduced but largely ineffective ban on below-tax alcohol sales [29], low- and high-strength duty rates covering small shares of the market [24], and the impact of tax fraud or retailer non-compliance with minimum unit prices. Whilst the operation of many tax avoidance schemes might be similar for the three tax policies (illicit production and trade, counterfeit labels, exploitation of EU tax rules, and use of false importation documents), it is possible that the scale of tax avoidance might vary between policies given the different revenue implications [38,39]. For minimum unit pricing, where the additional revenue from increased prices stays with the retailer, this policy non-compliance may be less of a problem but will require monitoring. No data currently exist that would allow us to derive assumptions on likely effects. Further, for beverage categories not currently taxed by their ethanol content (wine and cider), we assumed the alcoholic strength estimated from market research. A further limitation of the cohort-based harm modelling approach used in SAPM is that of “mortality selection” [40], although this is likely to be only a minor source of error in the results presented (see S1 Appendix, Section 9.7, for more details). Moreover, whilst considering population heterogeneity is key to understanding policy effects [41], the resulting level of disaggregation in the model means that some subgroups are represented by small numbers of HSE respondents and thus are subject to higher levels of uncertainty in terms of model outcomes. The effects of this are examined through the use of nonparametric bootstrapping in the PSA. The bootstrap method is the appropriate approach to take here as its use means that those subgroups with smaller sample sizes will have greater variance across samples in consumption levels and consequently alcohol-related deaths than subgroups with larger numbers within the HSE. The partial PSA presented here represents the most comprehensive evaluation to date of uncertainty in the outputs of SAPM, although, being partial, it does not account for either the full uncertainty across all parameters or the full intercorrelation of these uncertainties. An important avenue of future research in the development of complex models should be the development of robust methods to quantify uncertainty in parameters for which no published uncertainty data exist and, crucially, to estimate the correlation structure between potentially thousands of input parameters (as is the case for SAPM). Additional limitations typical to alcohol research apply, including the uncertainty about the size and distribution of cardio-protective effects of alcohol. No adjustment was made for the under-coverage of self-reported alcohol consumption relative to sales data [42] because most of the risk functions used in the model are also derived from survey data likely to suffer from similar under-coverage. Further epidemiological work should be a key focus of future research in order to facilitate a greater understanding of the impact of undercoverage on policy analyses.

There are several implications for national and international policymakers to consider. The results demonstrate that minimum unit pricing and volumetric taxation can target consumption and harm reductions in economically disadvantaged heavy drinkers in a way that duty increases under the current UK tax system cannot. For harmful drinkers only, both policies could be considered “regressive” with regard to consumption and spending, with a potentially associated loss of consumer satisfaction. However, the trade-off is that both policies are progressive with regard to the resulting health gains, which are concentrated in disadvantaged groups of harmful drinkers. These disadvantaged drinkers are at greater risk of health harm from high alcohol consumption than more advantaged counterparts consuming similar amounts [37]. Thus, minimum unit pricing and volumetric taxation would reduce health inequalities, precisely target those at greatest risk of harm, and avoid unnecessarily penalising those with low incomes who drink in moderation.

Minimum unit pricing is under consideration in several EU countries. Its legality under European Union law depends in part on its ability to achieve additional advantages or fewer disadvantages for public health and free trade compared to existing policy options, particularly tax increases under the current system (volumetric taxation is not currently possible in the EU as taxation of wine and cider by ethanol content is prohibited). This study suggests that, compared to a commensurate increase in existing alcohol taxes, minimum unit pricing is more targeted to the population at greatest risk of harm (i.e., disadvantaged heavy drinkers) and is thus more effective in reducing health inequalities, a key public health goal. Applying a larger tax increase could achieve a similar mortality reduction among disadvantaged heavy drinkers without similar reductions in health inequalities; however, given that UK alcohol taxes are already higher than in many comparable European countries, it is unclear whether an increased tax would be viewed as being even more disadvantageous to free trade than minimum unit pricing. Although a European court case was brought against the attempts by Austria, France, and Ireland to introduce minimum retail prices for tobacco in 2010, at that time there was limited evidence regarding the likely effects of minimum pricing on tobacco consumption, harm, and the distribution of harm reductions, or regarding differential undershifting of tax, such that cheap products are less affected by tax rise. At the time, the court concluded that public health objectives could be achieved by duty rises in combination with bans of below-cost selling (cost price plus all taxes) [43]. The current judgment may set legal precedent not just for minimum pricing for alcohol, but also for potential future policies relating to other commodities such as sugar and fat.

Although our analyses use a UK baseline, the findings have international relevance beyond their implications for European Union health law. The modelled taxation policies represent the predominant alcohol taxation structures globally, and almost every developed country in the world implements one of, or a mixture of, these policies [11]. While baseline patterns of drinking, beverage preferences, and mortality data are UK-specific, there is comparable evidence internationally that disadvantaged drinkers incur more harm for the same pattern of consumption [37] and that heavy drinkers disproportionately purchase lower priced products [44]. The implied overall elasticities used in the model are in line with meta-analytic estimates [7]. As lower priced products are targeted by minimum pricing and volumetric taxation, the general conclusions are expected to transfer to other countries. However, exact reductions in mortality and inequalities would vary, and it would be desirable to replicate our analyses for other countries. Future work should also address the likely effect of price policy combinations (such as tax rises plus minimum unit pricing, or VAT plus volumetric taxation).

There have been a number of international efforts to produce alcohol policy analysis models similar to SAPM [39,45], although these have lacked either the structural detail (e.g., the subgroup analyses and broad range of outcomes presented here) or the robust underlying data sources available in the UK. In order to internationalise our analyses, we have previously adapted SAPM to each of the four UK countries, Ireland, and Canada and, as part of this process, replaced English data with the best available local data where feasible [46–50]. These models have proved useful in policy debate, but repetition of this process for each country interested in, and requiring evidence on, minimum unit pricing would be inefficient. A means of adjusting policy effectiveness estimates to account for national variations in key parameters is required. We have previously addressed this problem using a “meta-modelling” approach when appraising variation in the cost-effectiveness of identification and brief advice policies aiming to reduce alcohol consumption in four European countries [51]. This meta-modelling used a regression approach to describe key model outputs as a function of national-level variables (e.g., baseline alcohol consumption, baseline levels of alcohol-attributable harm). This regression can then be used to appraise policies across a wider set of countries. A similar meta-model could potentially be constructed for alcohol pricing policies, although a key prerequisite would be sufficient data availability on key parameters (e.g., baseline alcohol consumption, spending, and alcohol-related harms) to permit construction of a heterogeneous set of national models on which the regression model could be based. To this end, international standardisation of relevant data would facilitate cross-national health policy analysis.

In conclusion, policy-induced price increases would lead to decreases in consumption and alcohol-related harm. There are, however, important differences in how well different policy options would target those who are most at risk of harm from their drinking. If achieving reductions in health inequalities is a priority, then the two policy options that target cheap, high-strength alcohol—minimum unit pricing and volumetric taxation—outperform ad valorem taxation and increasing the current UK tax. Importantly, unlike other tax options, these two policies target harmful drinking without at the same time targeting those in poorer population groups who do not engage in harmful drinking behaviour.

## Supporting Information

S1 Appendix Further details about model structure, model inputs, and sensitivity analyses. (PDF)

S1 Data Full details of the source for all input parameters to the model. (DOCX)

## Abbreviations

ABV: alcohol by volume
HSE: Health Survey for England
LCF: Living Costs and Food Survey
PSA: probabilistic sensitivity analysis
SAPM: Sheffield Alcohol Policy Model
UI: uncertainty interval
VAT: value added tax

## References

[1] Lim S, Vos T, Flaxman A, Danaei G, Shibuya K, Adair-Rohani H, et al A comparative risk assessment of burden of disease and injury attributable to 67 risk factors and risk factor clusters in 21 regions, 1990–2010: a systematic analysis for the Global Burden of Disease Study 2010. Lancet. 2013;380:2224–2260. 10.1016/S0140-6736(12)61766-8 PMC4156511 23245609

[2] Rehm J, Mathers C, Popova S, Thavorncharoensap M, Teerawattananon Y, Patra J. Global burden of disease and injury and economic cost attributable to alcohol use and alcohol-use disorders. Lancet. 2009;373:2223–2233. 10.1016/S0140-6736(09)60746-7 19560604

[3] Marmot M. Fair society, healthy lives: strategic review of health inequalities in England post-2010. London: The Marmot Review; 2010.

[4] Institute of Medicine. How far have we come in reducing health disparities? Progress since 2000: workshop summary. Washington (District of Columbia): National Academies Press; 2012. 23193624

[5] Gallet CA. The demand for alcohol: a meta-analysis of elasticities. Aust J Agric Resour Econ. 2007;51:121–135.

[6] Fogarty J. The nature of demand for alcohol: understanding elasticity. Br Food J. 2006;108:316–332.

[7] Wagenaar AC, Salois MJ, Komro KA. Effects of beverage alcohol price and tax levels on drinking: a meta-analysis of 1003 estimates from 112 studies. Addiction. 2009;104:179–190. 10.1111/j.1360-0443.2008.02438.x 19149811

[8] Wagenaar AC, Tobler AL, Komro KA. Effects of alcohol tax and price policies on morbidity and mortality: a systematic review. Am J Public Health. 2010;100:2270–2278. 10.2105/AJPH.2009.186007 20864710 PMC2951962

[9] van den Berg M, van Baal PH, Tariq L, Schuit AJ, de Wit GA, Hoogenveen RT. The cost-effectiveness of increasing alcohol taxes: a modelling study. BMC Med. 2008;6:36 10.1186/1741-7015-6-36 19040717 PMC2637894

[10] Chisholm D, Rehm J, Van Ommeren M, Monteiro M. Reducing the global burden of hazardous alcohol use: a comparative cost-effectiveness analysis. J Stud Alcohol. 2004;65:782–793. 15700517 10.15288/jsa.2004.65.782

[11] Rehm J, Shield KS. Taxation and pricing policies among countries worldwide: results from the 2010 global survey on alcohol and health In: Sornpaisarn B, Shield KS, Österberg E, Rehm J, editors. A resource tool on alcohol taxation and pricing policies. Bangkok: ThaiHealth Promotion Foundation; 2014 pp. 55–61.

[12] Gornall J. Alcohol and public health. Under the influence. BMJ. 2014;348:f7646 10.1136/bmj.f7646 24401176

[13] The Scotch Whisky Association and Others v The Lord Advocate, The Advocate General for Scotland. Opinion of Advocate General Bot. Court of Justice of the European Union; 2015.

[14] Holmes J, Meng Y, Meier PS, Brennan A, Angus C, Campbell-Burton CA, et al Effects of minimum unit pricing for alcohol on different income and socioeconomic groups: a modelling study. Lancet. 2014;383:1655–1664. 10.1016/S0140-6736(13)62417-4 24522180 PMC4018486

[15] Staras SAS, Livingston MD, Christou AM, Jernigan DH, Wagenaar AC. Heterogeneous population effects of an alcohol excise tax increase on sexually transmitted infections morbidity. Addiction. 2014;109:904–912. 10.1111/add.12493 24450730 PMC4013225

[16] Byrnes J, Petrie DJ, Doran CM, Shakeshaft A. The efficiency of a volumetric alcohol tax in Australia. Appl Health Econ Health Policy. 2012;10:37–49. 10.2165/11594850-000000000-00000 22181353

[17] Doran CM, Byrnes JM, Cobiac LJ, Vandenberg B, Vos T. Estimated impacts of alternative Australian alcohol taxation strcutures on consumption, public health and government revenues. Med J Aust. 2013;199:619–622. 24182229 10.5694/mja13.10605

[18] Sharma A, Vandenberg B, Hollingsworth B. Minimum pricing of alcohol versus volumetric taxation: which policy will reduce heavy consumption without adversely affecting light and moderate consumers? PLoS ONE. 2014;9:e80936 10.1371/journal.pone.0080936 24465368 PMC3898955

[19] Sornpaisarn B, Shield KD, Rehm J. Alcohol taxation in Thailand: implications for other low- to middle-income countries. Addiction. 2014;107:1372–1384. 10.1111/j.1360-0443.2011.03681.x 22324742

[20] Fogarty JJ. Optimal alcohol taxes for Australia. Forum Health Econ Policy. 2012 10.1515/1558-9544.1276 31419856

[21] Sornpaisarn B, Kaewmungkun C, Rehm J. Assessing patterns of alcohol taxes produced by various types of excise tax methods: a simulation study. Alcohol Alcohol. 2015;50:639–646. 10.1093/alcalc/agv065 26094246

[22] Purshouse RC, Meier PS, Brennan A, Taylor KB, Rafia R. Estimated effect of alcohol pricing policies on health and health economic outcomes in England: an epidemiological model. Lancet. 2010;375:1355–1364. 10.1016/S0140-6736(10)60058-X 20338629

[23] Her Majesty’s Revenue and Customs. Rates and allowances: excise duty—alcohol duty. 2014 Mar 1 [cited 12 Dec 2014]. Available: http://www.hmrc.gov.uk/rates/alcohol-duty.htm.

[24] Leicester A. Alcohol pricing and taxation policies IFS Briefing Note BN124. London: Institute for Fiscal Studies; 2011.

[25] Office for National Statistics. Living Costs and Food Survey: user guide volume A—introduction. London: Her Majesty’s Stationery Office; 2010.

[26] Ally AK, Meng Y, Chakroborty R, Dobson PW, Seaton JS, Holmes J, et al Alcohol tax pass-through across the product and price range: do retailers treat cheap alcohol differently? Addiction. 2014;109:1994–2002. 10.1111/add.12590 24957220

[27] Health and Social Care Information Centre. Health Survey for England, 2012: health, social care and lifestyles. London: Health and Social Care Information Centre; 2013.

[28] Meng Y, Brennan A, Purshouse R, Hill-MacManus D, Angus C, Holmes J, et al Estimation of own and cross price elasticities of alcohol demand in the UK: a pseudo-panel approach using the Living Costs and Food Survey 2001–2009. J Health Econ. 2014;34:96–103. 24508846 10.1016/j.jhealeco.2013.12.006 PMC3991422

[29] Brennan A, Meng Y, Holmes J, Hill-McManus D, Meier PS. Potential benefits of minimum unit pricing for alcohol versus a ban on below cost selling in England 2014: modelling study. BMJ. 2014;349:g5452 10.1136/bmj.g5452 25270743 PMC4180296

[30] Hill-McManus D, Angus C, Meng Y, Holmes J, Brennan A, Sylvia Meier P. Estimation of usual occasion-based individual drinking patterns using diary survey data. Drug Alcohol Depend. 2014;134:136–143. 10.1016/j.drugalcdep.2013.09.022 24128380

[31] Hill-Mcmanus D, Angus C, Meng Y, Holmes J, Brennan A, Meier P. Injury alcohol-attributable fractions: methodological issues and developments. Sheffield: University of Sheffield; 2014.

[32] Jones L, Bellis MA. Updating England-specific alcohol-attributable fractions. Liverpool: Centre for Public Health, Liverpool John Moores University; 2014.

[33] Siegler V, Al-Hamad A, Johnson B, Wells C, Sheron N. Social inequalities in alcohol-related adult mortality by National Statistics Socio-economic Classification, England and Wales, 2001–03. Health Stat Q. 2011;50:1–36. 10.1057/hsq.2011.7 21647087

[34] Holmes J, Meier PS, Booth A, Guo Y, Brennan A. The temporal relationship between per capita alcohol consumption and harm: a systematic review of time lag specifications in aggregate time series analyses. Drug Alcohol Depend. 2012;123:7–14. 10.1016/j.drugalcdep.2011.12.005 22197480

[35] Purshouse RC, Brennan A, Latimer N, Meng Y, Rafia R, Jackson R. Modelling to assess the effectiveness and cost-effectiveness of public health related strategies and interventions to reduce alcohol attributable harm in England using the Sheffield Alcohol Policy Model version 2.0: report to the NICE Public Health Programme Development Group. Sheffield: University of Sheffield; 2009.

[36] Sousa J. Estimation of price elasticities of demand for alcohol in the United Kingdom. London: Her Majesty’s Revenue and Customs; 2014.

[37] Mäkelä P, Paljärvi T. Do consequences of a given pattern of drinking vary by socioeconomic status? A mortality and hospitalisation follow-up for alcohol-related causes of the Finnish Drinking Habits Survey. J Epidemiol Community Health. 2008;62:728–733. 10.1136/jech.2007.065672 18621959

[38] Her Majesty’s Revenue and Customs, Her Majesty’s Treasury. 2010 to 2015 government policy: tax evasion and avoidance. London: Her Majesty’s Revenue and Customs; 2015.

[39] Sassi F, Cecchini M, Devaux M, Astolfi R. Health and economic impacts of key alcohol policy options In: Organization for Economic Co-operation and Development, editor. Tackling harmful alcohol use: economics and public health policy. Paris: OECD Publishing; 2015.

[40] Brønnum-Hansen H. How good is the Prevent model for estimating the health benefits of prevention? J Epidemiol Community Health. 1999;53:300–305. 10396537 10.1136/jech.53.5.300 PMC1756876

[41] Meier PS, Purshouse R, Brennan A. Policy options for alcohol price regulation: the importance of modelling population heterogeneity. Addiction. 2010;105:383–393. 10.1111/j.1360-0443.2009.02721.x 19839965

[42] Meier PS, Meng Y, Holmes J, Baumberg B, Purshouse R, Hill-MacManus D, et al Adjusting for unrecorded consumption in survey and per capita sales data: quantification of impact on gender- and age-specific alcohol attributable fractions for oral and pharyngeal cancers in Great Britain. Alcohol Alcohol. 2013;48:241–249. 23345391 10.1093/alcalc/agt001

[43] Cases C-197/08 Commission v France, C-198/08 Commission v Austria and CC-221/08 Commission v Ireland. Court of Justice of the European Union; 2010.

[44] Kerr WC, Greenfield TK. Distribution of alcohol consumption and expenditures and the impact of improved measurement on coverage of alcohol sales in the 2000 National Alcohol Survey. AlcoholClin Exp Res. 2007;31:1714–1722. 10.1111/j.1530-0277.2007.00467.x 17651465

[45] White J, Lynn R, Ong SW, Whittington P, Condon C, Joy S. The effectiveness of alcohol pricing policies. Wellington (New Zealand): Ministry of Justice; 2014.

[46] Meng Y, Sadler S, Gell L, Holmes J, Brennan A. Model-based appraisal of minimum unit pricing for alcohol in Wales: an adaptation of the Sheffield Alcohol Policy Model version 3. Sheffield: School of Health and Related Research, University of Sheffield; 2014.

[47] Meng Y, Hill-MacManus D, Brennan A, Meier P. Model-based appraisal of alcohol minimum pricing and off-licensed trade discount bans in Scotland using the Sheffield Alcohol Policy Model (v 2): second update based on newly available data. Sheffield: School of Health and Related Research, University of Sheffield; 2012.

[48] Angus C, Meng Y, Ally A, Holmes J, Brennan A. Model-based appraisal of minimum unit pricing for alcohol in Northern Ireland: an adaptation of the Sheffield Alcohol Policy Model version 3. Sheffield: School of Health and Related Research, University of Sheffield; 2014.

[49] Angus C, Meng Y, Ally A, Holmes J, Brennan A. Model-based appraisal of minimum unit pricing for alcohol in the Republic of Ireland: an adaptation of the Sheffield Alcohol Policy Model version 3. Sheffield: School of Health and Related Research, University of Sheffield; 2014.

[50] Hill-McManus D, Brennan A, Stockwell T, Giesbrecht N, Thomas G, Zhao J, et al Model-based appraisal of alcohol minimum pricing in Ontario and British Columbia: a Canadian adaptation of the Sheffield Alcohol Policy Model Version 2. Sheffield: School of Health and Related Research, University of Sheffield; 2012.

[51] Angus C, Scafato E, Ghirini S, Torbica A, Ferre F, Struzzo P, et al Optimising delivery of health care interventions (ODHIN): cost-effectiveness model report. Sheffield: University of Sheffield; 2013.

