# Supplementary material for: Estimated Effects of Different Alcohol Taxation and Price Policies on Health Inequalities: A Mathematical Modelling Study
Source: PLoS Med. 2016 Feb 23;13(2):e1001963. doi: 10.1371/journal.pmed.1001963 (PMC4764336; doi:10.1371/journal.pmed.1001963)
Supplement: S1 Appendix — (PDF) [file pmed.1001963.s001.pdf]

Comparing the effects of different alcohol taxation and price policies on health inequalities:

Technical Appendix

Produced by Colin Angus, Abdallah Ally, Tony Stone, Yang Meng, John Holmes, Daniel Hill-McManus, Alan Brennan, & Petra Meier

© ScHARR, University of Sheffield, November 2015

CONTENTS

Contents 2

Index of Tables 5

Index of Figures 7

1 Overview of the Sheffield Alcohol Policy Model 8

2 Discussion of Issues Relating to the Quantification of Alcohol Consumption in England 10

3 Alcohol Consumption Data 12

3.1 Health Survey for England 12

3.2 Mean Weekly Consumption 12

3.3 Peak Day Consumption in Most recent week 12

3.4 Relating Peak Day Maximum Consumption to Mean Consumption Using the hse13

3.5 Relating Further Single Occasion Drinking Parameters to Mean Consumption Using the HSE 14

4 Prices Paid Data 17

4.1 Living Costs and Food Data 17

4.2 Market research Data on the Price Distribution of Alcohol Sold in the On- and Off-Trades 20

4.3 Accounting for Price Inflation 22

4.4 Adjusting LCF/EFS Data using Nielsen and CGA Data 23

4.5 Estimating Beverage Preferences 27

5 Price Elasticities of Alcohol Demand 28

6 Modelling the Impact of Pricing Policies on Alcohol Prices 30

6.1 Modelling the UK baseline tax system 30

6.2 Modelling differential pass-through of tax increases by product and baseline price 32

7 Price to Consumption Model 34

7.1 Model Overview 34

7.2 Modelling the 'No Policy' Scenario 35

8 Consumption to Health Harms Model 37

8.1 Health Model Structure 37
8.2 Mortality Model Structure 39
9 Approach to Modelling the Relationship between Consumption and Harm 41
9.1 Model Structure 41
9.2 Alcohol-Attributable Fractions and Potential Impact Fractions 41
9.3 Derivation of Risk Functions 43
9.3.1 Relative risk functions already available in the published literature 43
9.3.2 Modelling partially attributable acute health harms 46
9.3.3 Absolute risk functions for wholly attributable harms 46
9.4 Time Lag Effects for Chronic Harms 48
9.5 Mortality Model Parameters 50
9.6 Deriving socioeconomic classification-specific mortality rates 50
9.6.1 Step 1: Derive baseline age, gender and health condition-specific mortality rates for use as the basis for adjustments 50
9.6.2 Step 2: Derivation of age, gender and SEC-specific adjustment factors for alcohol-related mortality rates. 50
9.6.3 Step 3: Estimation of SEC-specific alcohol-related mortality rates using SAPM53
9.6.4 Step 4: Modifying adjustment factors to account for SEC-related differences in drinking patterns already modelled in SAPM 54
9.7 Mortality Selection in SAPM 55
10 Partial Mortality Results 56
11 Deterministic Sensitivity Analysis 57
11.1 Alternative elasticity estimates 57
11.2 Results 59
12 Probabilistic Sensitivity Analysis 60
12.1 Rationale 60
12.2 Approaches to quantifying uncertainty 60
12.2.1 Baseline consumption data 60
12.2.2 Baseline price data 61
12.2.3 Price elasticities 61

12.2.4 Tax pass-through ... 61
12.2.5 Mortality risk functions ... 61
12.4 PSA Results ... 64
Reference List ... 76

Index of Tables

Table 1.1: Lists of categories by which population subgroups are defined 8
Table 3.1: Statistical regression model estimating number of occasions per week and two-stage model estimating standard deviation in consumption across occasions. 16
Table 4.1: Matching of LCF/EFS product categories to modelled categories and ABV estimates 19
Table 4.2: Price ranges for the Nielsen data 21
Table 4.3: ONS alcohol-specific RPIs 2001 to 2014 22
Table 5.1: Estimated own- and cross-price elasticities for off- and on-trade beer, cider, wine, spirits and RTDs in the UK 29
Table 6.1: Alcohol duty rates in the UK from 24th March 2014 31
Table 6.2: Modelled duty rates by beverage type 31
Table 6.3: Pass-through adjustment factors by price band and beverage 33
Table 8.1: Health conditions included in the model 38
Table 9.1: Modelled lag times between changes in consumption and changes in mortality risk 49
Table 9.2: Male alcohol-related mortality rates (per 100,000) by age and SEC classification, England and Wales, 2001-2003 51
Table 9.3: Female alcohol-related mortality rates (per 100,000) by age and SEC classification, England and Wales, 2001-2003 51
Table 9.4: Males alcohol-related mortality rates (per 100,000) by SAPM age groups and SEC classification, England and Wales, 2001-2003 52
Table 9.5: Females alcohol-related mortality rates (per 100,000) by SAPM age groups and SEC classification, England and Wales, 2001-2003 52
Table 9.6: Male ratios of the mortality rate of each age-sex-SEC group to the mortality rate of the age-sex group, England and Wales, 2001-2003 52
Table 9.7: Female ratios of the mortality rate of each age-sex-SEC group to the mortality rate of the age-sex group, England and Wales, 2001-2003 53
Table 9.8: SAPM-derived estimated alcohol-related deaths wholly attributable to alcohol and mortality rates and ratios of the alcohol-attributable mortality rate of each SEC group to the alcohol-attributable rate of the population 53
Table 9.9: Male adjustment factors for alcohol-related mortality rates by NS-SEC group 54
Table 9.10: Female adjustment factors for alcohol-related mortality rates by NS-SEC group 54
Table 10.1: Change in annual deaths in years 1-20 for all policies 56
Table 11.1: HMRC elasticity estimates 58

Table 11.2: Meng et al. elasticity estimates (for comparison) 58
Table 11.3: Comparison of modelled impacts on consumption and deaths under baseline and alternative elasticitiy assumptions 59
Table 12.1: Summary of sampling methods and sampled distributions for partial PSA:....... 63
Table 12.2: PSA impacts of modelled policies on alcohol consumption by drinker group 64
Table 12.3:PSA impacts of modelled policies on alcohol consumption by income quintile .. 65
Table 12.4: PSA impacts of modelled policies on spending on alcohol by drinker group 66
Table 12.5: PSA impacts of modelled policies on spending on alcohol by income quintile .. 67
Table 12.6: PSA impacts of modelled policies on annual deaths due to alcohol by drinker group 68
Table 12.7: PSA impacts of modelled policies on annual deaths due to alcohol by socioeconomic group 69
Table 12.8: Mean PSA results with 95% Uncertainty Intervals for absolute changes in alcohol consumption 70
Table 12.9: Mean PSA results with 95% Uncertainty Intervals for relative changes in alcohol consumption 71
Table 12.10: Mean PSA results with 95% Uncertainty Intervals for absolute changes in spending on alcohol 72
Table 12.11: Mean PSA results with 95% Uncertainty Intervals for relative changes in spending on alcohol 73
Table 12.12: Mean PSA results with 95% Uncertainty Intervals for absolute changes in annual deaths (full effect) 74
Table 12.13: Mean PSA results with 95% Uncertainty Intervals for relative changes in annual deaths (full effect) 75

Index of Figures

Figure 1.1: Schematic on integrating data sources 9
Figure 3.1: Illustrative example of binge relationship in males aged 35 to 44 14
Figure 4.1: Impact of Nielsen and CGA adjustments on beverage-specific and overall price distributions 26
Figure 7.1: Model construction steps: creation of a “new HSE” and Nielsen/CGA adjusted LCF/EFS dataset 35
Figure 8.1: Simplified mortality model structure 39
Figure 9.1: Relative Risk functions for partially alcohol-attributable chronic health conditions 45
Figure 9.2: Illustrative linear absolute risk function for a wholly attributable acute harm (threshold of 4 units) 47

1 OVERVIEW OF THE SHEFFIELD ALCOHOL POLICY MODEL

The aim of SAPM3 is to appraise the impact of alcohol pricing policy options on a range of health care and societal outcomes. We have broken down the aims into a linked series of policy impacts to be modelled

- The effect of the policy on the distribution of prices for different types of alcohol;
- The effect of changes in price distributions on patterns of both on-trade and off-trade alcohol consumption;
- The effect of changes in alcohol consumption patterns on levels of alcohol-related health harms;

To estimate these effects, two connected models have been built:

a) A model of the relationship between alcohol prices and alcohol consumption which accounts for the relationship between mean weekly and peak daily consumption and how consumption is distributed within the population. These relationships are modelled for both the total population and for population subgroups defined by age, sex, consumption level and income or socioeconomic status (see Table 1.1).
b) A model of the relationship between (1) mean weekly consumption and single occasion consumption measures and (2) alcohol-related mortality.

Figure 1.1 indicates the main datasets used to provide different aspects of the picture. The model links evidence from these datasets to enable comprehensive appraisals of the potential impacts of a policy on a range of outcomes of interest.

Table 1.1: Lists of categories by which population subgroups are defined

**Age**
18 – 24
25 – 34
35 – 54
55+

**Sex**
Male
Female

**Consumption Level**[1]
Moderate
Increasing risk
Heavy

**Income**
Quintiles of equivalised household income

**Socioeconomic status**
Professional / managerial
Intermediate
Routine / manual

[1] See Section 2 for definitions

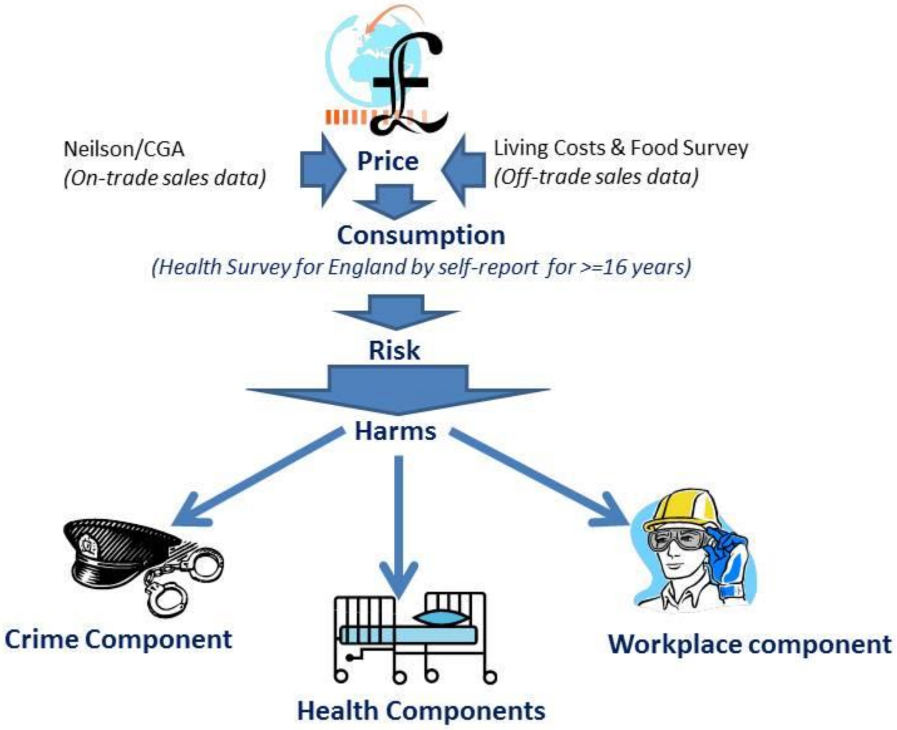

Figure 1.1: Schematic on integrating data sources

2 DISCUSSION OF ISSUES RELATING TO THE QUANTIFICATION OF ALCOHOL CONSUMPTION IN ENGLAND

One major aspect in the development of SAPM3 was to integrate datasets on price and consumption due to the absence of an English dataset covering both of these components. While the Health Survey for England (HSE) provides good estimates of subgroup-specific alcohol consumption patterns in England, it does not contain information on purchasing. In particular, it provides no information on how much was paid for alcohol consumed or whether it was purchased in the on-trade or the off-trade. Conversely, while the Living Costs and Food Survey (LCF) provides a good picture of alcohol purchasing in England, a consumption distribution based on this dataset may not reflect accurately patterns of consumption in England at the subgroup level, as it only covers a two week diary period and purchasers of alcohol are not necessarily the consumers. The link between price and consumption was thus modelled using different datasets.

Population surveys continue to provide the main approach to assessing sub-population-level alcohol consumption in England. Such surveys ask respondents about the volume of certain types of alcoholic beverages bought or consumed over a certain time period. The reported beverage volumes are then converted to units of alcohol (1 UK unit = 8g/10ml of pure ethanol) by a methodology outlined in Section 3.2. From 2006, UK government surveys have started to implement a revised methodology of unit counting which addresses several problems related to the underestimation of consumption [1].

It is generally accepted that this self-reported data continues to underestimate actual consumption by as much as 50% [2]. For example, in the British General Household Survey in 2005, males and females reported a mean weekly alcohol consumption of 15.8 units and 6.5 units respectively [3], whereas the estimate for all adults based on clearance data from HMRC was 21.9 units [4]. It is important to understand not only the magnitude of such underestimation, but also the potential biases which lead to it. These include:

- **Under-sampling:** household surveys under-represent some of the groups who drink the most (e.g. those in unstable living conditions and dependent drinkers) [2,5]
- **Variation in under-reporting by pattern of consumption:** when asked about typical drinking, people may not take into account heavy drinking occasions [1,2].
- **Variation in under-reporting by drinker type:** underestimation of consumption may vary across the population in ways which are largely unquantified to date [2,6].

- **Variation in under-reporting by beverage type:** underestimation is greater for some beverage types than others and this partially reflects drinkers' uncertainty regarding the quantity of beverage, particularly spirits, consumed when pouring their own drinks [2,5].

For analytical purposes a key task is the classification of drinkers in terms of typical alcohol intake per week and how their intake is distributed across individual drinking occasions (their drinking pattern).

Consistent with other analyses [7], in this analysis drinkers in England are classified into one of three drinking categories based on their mean intake per week:

- *Moderate drinkers* – drinkers with an intake of alcohol less likely to damage health and/or be associated with negative consequences (less than 21 units per week for men; less than14 units per week for women). This category broadly corresponds to drinking within the current UK drinking guidelines.
- *Increasing risk drinkers* – drinkers with an increased risk of psychological consequences (such as mood disturbance) and physical consequences (such as injuries) due to alcohol intake (more than or equal to 21 but less than 50 units per week for men; more than or equal to 14 but less than 35 units for women).
- *Heavy drinkers* – drinkers with an intake that is likely to adversely affect health and/or have other negative consequences (more than or equal to 50 units per week for men; more than or equal to 35 units per week for women).

Additionally, an individual is classified as a *binge drinker* in some analyses if he or she exceeds a certain maximum intake of alcohol during a single occasion. A binge is commonly defined as an intake of over twice the recommended daily limit in the UK Chief Medical Officer's low risk drinking guidelines (ie. over 8 units per day for men and over 6 units per day for women). Binge drinking can and does occur in each of the three drinking categories above; however both the likelihood and scale of the binge (how much is drunk on each occasion) are strongly associated with mean consumption. SAPM3 adopts a new approach to modelling the risks of occasion-specific consumption which is set out in Section 3.5.

3 ALCOHOL CONSUMPTION DATA

3.1 HEALTH SURVEY FOR ENGLAND

Estimates of alcohol consumption for people in England aged 16 and over are taken from the Health Survey for England (HSE). The HSE is an annual cross-sectional survey of individuals living in private households in England. Along with detailed demographic characteristics (such as age, sex, and income), respondents are asked how often over the last year they have drunk each of four different types of alcoholic beverage (beer and cider, wine, spirits, and RTDs[1]), and how much they have usually drunk of each beverage type on any one drinking day. Respondents are also asked how much of the four different types of alcoholic beverage they have consumed on the heaviest drinking day in the past week. The volumes drunk are then standardised by converting them into units of alcohol. The conversion of reported volumes to units is based on assumptions made by the ONS [1] about the average alcohol by volume (ABV) content of the different beverage types.

In terms of limitations, the HSE does not provide:

- Information on some at-risk groups (e.g. homeless people and others not living in private households).
- Information on whether binge drinking occurred on more than one occasion in the past week or how typical this is for the respondent

Data for the most recent available year of the HSE (at the time of analysis) is used to represent baseline consumption in the model. In this analysis the baseline consumption year is 2012.

3.2 MEAN WEEKLY CONSUMPTION

The method used for calculating mean weekly consumption for an individual is to multiply the number of units of each type of beverage drunk on a usual drinking day by the frequency with which the beverage was drunk and then to sum these values across the four beverage types. The mean weekly consumption was capped at a maximum value of 300 units.

3.3 PEAK DAY CONSUMPTION IN MOST RECENT WEEK

The method used for calculating peak day consumption for an individual is to sum the number of units of each type of beverage drunk on the heaviest drinking day in the past week. Peak day consumption provides a measure of one dimension of binge drinking

[1] RTDs – ready-to-drinks (also known as alcopops or pre-mixed drinks)

behaviour. The proportion of respondents reporting zero consumption is larger for peak day consumption than for mean weekly consumption as it is based only on drinking in the survey week rather than the last year.

3.4 RELATING PEAK DAY MAXIMUM CONSUMPTION TO MEAN CONSUMPTION USING THE HSE

As in previous versions of the model, the price elasticities used in SAPM3 relate a change in price to a change in mean consumption; therefore an additional step is required to estimate the effects of a change in price on peak daily consumption This is achieved by estimating the average relationship between relative change in mean weekly consumption and relative change in peak daily consumption at subgroup level and using this relationship to estimate how individuals' peak daily consumption changes following a change in mean weekly consumption.

One main advantage of the HSE is the availability of data for both the mean weekly intake (here converted to mean daily intake) and peak day intake. It was thus possible to map the scale of binge from the mean intake using standard statistical regression model techniques. Separate linear models were constructed for each drinker type due to the anticipated differences in behaviour of moderate, increasing risk and high risk drinkers. For each age group and sex, the models predict the peak day intake from the mean daily intake of alcohol.

The regression models are used to predict the relative change in the scale of binging between baseline and an intervention. The relative change is then applied to the baseline units of alcohol drunk on the heaviest drinking day (original data from the HSE). Figure 3.1 provides an illustrative example of the three models plotted for males aged 35 to 54 years. The gradient of the regression models are less steep as the daily intake of alcohol increase.

To illustrate the functionality of the binge model, consider a male aged 40 with a mean daily intake at baseline of 3.5 units (i.e. an increasing risk drinker) who drank 10 units on the heaviest drinking day. Consider a policy that reduces the mean daily intake by 1 unit. This changes the mean consumption from 3.5 units to 2.5 units, a reduction of 29%. The regression model for an increasing risk drinker with mean daily consumption of 3.5 units predicts a maximum consumption on the heaviest day of 8.6 units. The same regression model is extrapolated for a mean daily consumption of 2.5 units and predicts a maximum consumption on the heaviest day of 7.7 units, i.e. a reduction of 11% in the scale of the binge. The predicted maximum consumption on the heaviest day for the selected individual would thus be reduced by 11%, from 10 units to 8.9 units.

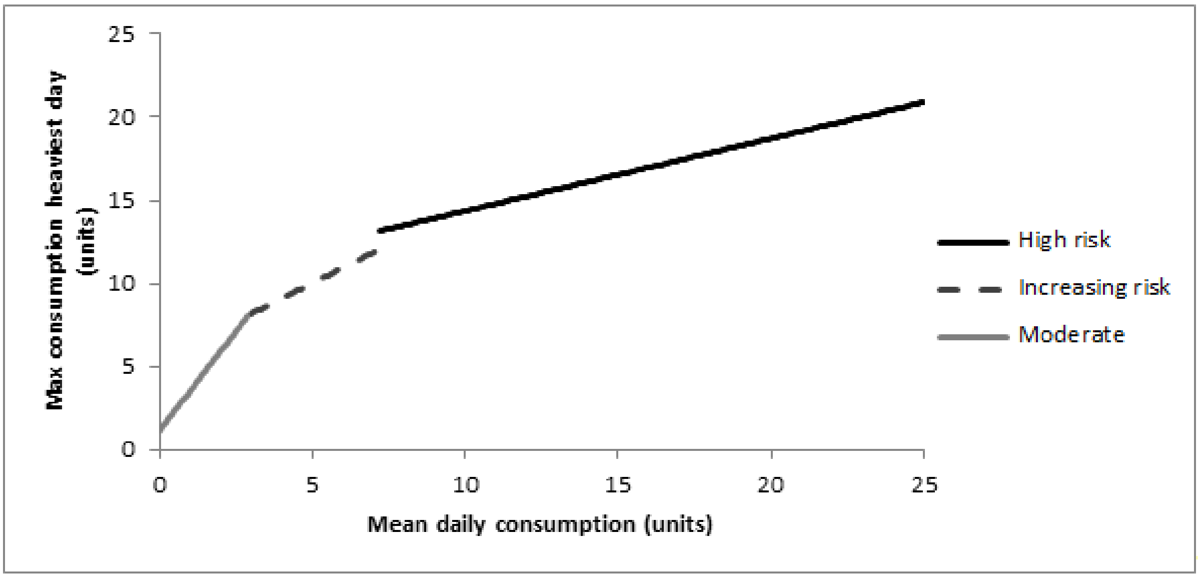

Figure 3.1: Illustrative example of binge relationship in males aged 35 to 44

3.5 RELATING FURTHER SINGLE OCCASION DRINKING PARAMETERS TO MEAN CONSUMPTION USING THE HSE

For version 3, SAPM has been developed to incorporate new methods of modelling the relationship between single occasion consumption and risks of acute harms partially attributable to alcohol (e.g. falls, road traffic injuries etc.). These methods are based on three additional measures of single occasion drinking; specifically the number of drinking occasions per week, the mean level of drinking on a given occasion and the variability of consumption across drinking occasions. As these three measures are not present in HSE, values for number of occasions per week and variability of consumption across occasions were imputed for HSE individuals based on previously reported regression analyses [8]. Mean consumption on a given occasion was calculated from mean weekly consumption and number of occasions per week.

The regression analyses fitted models which relate the three measures of single occasion drinking to mean consumption and a range of independent variables including age, gender, education and ethnicity. As the regression models were estimated using data taken from one week drinking diaries, reliable estimation of the standard deviation in consumption across drinking across occasions was only possible for drinkers with at least three occasions in the diary week. To address the resultant problem of selection bias, a two-step Heckman regression was used [9].

The regression model used in SAPM3 to relate mean weekly consumption to number of drinking occasions per week and the two-stage Heckman model used to estimate standard deviation in consumption across occasions are shown in Table 3.1. Full details of all regression models and methods of estimation are available elsewhere [8].

Table 3.1: Statistical regression model estimating number of occasions per week and two-stage model estimating standard deviation in consumption across occasions.

Independent variables	Number of weekly occasions	Heckman selection model†	Standard deviation in consumption per occasion
Log-weekly mean consumption	0.422	0.592	0.829
Age group: 19-24	-	-	-
Age group: 25-34	0.323	0.686	-
Age group: 35-44	0.467	0.757	-
Age group: 45-54	0.661	0.978	-
Age group: 55-64	0.745	1.092	-
Income: not in poverty	-	-	-
Income: in poverty	-0.168	-0.310	-0.438
Ethnicity: White	-	-	-
Ethnicity: Non-white	-0.254	-.0574	-
Age left education: None	-0.413	-0.380	-
Age left education: 15	-0.146	-0.545	-
Age left education: 16-18	-0.220	-0.395	-
Age left education: 19+	-	-	-
Children: none	-	-	-
Children: 1	-0.037	-0.032	-
Children: 2	0.137	0.205	-
Children: 3+	-0.166	-0.253	-
Socioeconomic status: non-manual	-	-	-
Socioeconomic status: manual	-0.221	-0.285	-
Employed		-	-
Not employed		0.062	-
Constant	0.063	-1.349	-
alpha††	0.175	-	-
Number of Observations	1548	1548	725
Inverse Mills Ratio	-	-	1.194
McFadden's R^2	0.101	0.298	-
ML (Cox-Snell) R^2	0.359	0.336	-
R^2	-		0.894

† Selection for probability that an individual drinks on at least three separate occasions during the diary period. †† Negative binomial dispersal term.

4 PRICES PAID DATA

4.1 LIVING COSTS AND FOOD DATA

The Living Cost and Food Survey (LCF), previously known as the Expenditure and Food Survey (EFS), is an annual survey of around 6,500 households in the United Kingdom. It records the purchasing of a range of goods, via a diary system for the individual over a two week period. Parents keep diaries for children under 16, whilst over 16s complete their own diary. In general, LCF records the amount of a good bought, the price paid by the purchaser and the type of outlet where the purchase was made.

The standard LCF/EFS data is available from the UK Data Archive; however anonymised transaction-level LCF/EFS diary data for individuals was obtained directly from DEFRA after a special data request. Anonymised individual-level diary data on 25 categories of alcohol (e.g., off-trade beers, see Table 4.1 for a complete list) detailing both expenditure (in pence) and quantity (in natural volume of product) were made available to the authors. England-only LCF/EFS transaction data for the nine years from 2001/2 to 2009 is used with a total sample size of 227,933 purchasing transactions. These transactions were used for constructing the baseline empirical price distributions for each modelled subgroup and each of ten modelled beverage types (off/on-trade: beer, cider, wine, spirits, and RTD).

Some limitations of the LCF/EFS need to be taken into consideration:

- A low response rate of 55.6% of all approached households for survey years 2001/2 to 2009, with potentially important differences in the response rates by age, social class and educational status [10].

The resulting data allows an assessment for each individual of:

- The price paid, type of alcohol, volume of beverage and hence number of units purchased. This is split by beverage type (beer, cider, wine, spirits, and RTDs) and by on-trade versus off-trade purchasing. Aggregation of lower-level beverage types and assumed ABVs are provided in Table 4.1.
- Mean units per week purchased over the two weeks (split as above), providing a proxy for mean consumption.
- Units purchased on each day during the two weeks. Although off-trade purchasing may be consumed over several days or weeks (see below), on-trade purchasing probably provides a satisfactory proxy of actual consumption.

- Purchasers’ individual characteristics including age, sex, income, education.

The LCF/EFS does not provide:

- Information on actual consumption of alcohol – only purchasing and prices paid.
- Information on some high-risk groups not covered by household surveys (e.g. those who are homeless and others not living in private households).

It is clear that off-trade purchasing on a particular day may bear little relationship to actual consumption that day since the purchase can be stored and consumed later. It is also the case that at a population level, the fortnightly purchasing distribution from the LCF/EFS may bear some relationship to the mean weekly consumption from HSE. Comparison of this with the analogous HSE distribution shows that a higher proportion of the population are towards either end of the distribution in the LCF/EFS and fewer in the middle area of the distribution. This is firstly because many of the people who purchased no alcohol in the LCF/EFS may have purchased just before or just after the fortnight diary. Secondly, some of the ‘high risk purchase’ from LCF/EFS may be shared with other individuals in terms of consumption. This comparison underlines the need to utilise HSE as the baseline for consumption patterns, and to make some form of link to LCF/EFS, which has the data combining purchases and prices paid.

Table 4.1: Matching of LCF/EFS product categories to modelled categories and ABV estimates

LCF off/on trade	LCF category	Modelled category	ABV estimate
Off-trade	Beers	off-trade beer	3.9%
Off-trade	Lagers and continental beers	off-trade beer	3.9%
Off-trade	Ciders and perry	off-trade cider	4.8%
Off-trade	Champagne, sparkling wines and wine with mixer	off-trade wine	11.2%
Off-trade	Table wine	off-trade wine	12.7%
Off-trade	Spirits with mixer	off-trade spirits	7.3%
Off-trade	Fortified wines	off-trade wine	14.3%
Off-trade	Spirits	off-trade spirits	39.6%
Off-trade	Liqueurs and cocktails	off-trade spirits	33.3%
Off-trade	Alcopops	off-trade RTD	4.6%
On-trade	Spirits	on-trade spirits	41.8%
On-trade	Liqueurs	on-trade spirits	29.9%
On-trade	Cocktails	on-trade spirits	13.2%
On-trade	Spirits or liqueurs with mixer	on-trade spirits	7.7%
On-trade	Wine (not sparkling) including unspecified 'wine'	on-trade wine	11.1%
On-trade	Sparkling wines and wine with mixer (e.g. Bucks Fizz)	on-trade wine	9.5%
On-trade	Fortified wine	on-trade wine	17.3%
On-trade	Cider or perry - half pint or bottle	on-trade cider	4.8%
On-trade	Cider or perry - pint or can or size not specified	on-trade cider	4.8%
On-trade	Alcoholic soft drinks (alcopops), and ready-mixed bottled drinks	on-trade RTDs	4.6%
On-trade	Bitter - half pint or bottle	on-trade beer	4.3%
On-trade	Bitter - pint or can or size not specified	on-trade beer	4.3%
On-trade	Lager or other beers including unspecified 'beer' - half pint or bottle	on-trade beer	5.0%
On-trade	Lager or other beers including unspecified 'beer' - pint or can or size not specified	on-trade beer	5.0%
On-trade	Round of drinks, alcohol not otherwise specified	on-trade beer	4.8%

4.2 MARKET RESEARCH DATA ON THE PRICE DISTRIBUTION OF ALCOHOL SOLD IN THE ON- AND OFF-TRADES

In 2012 updated data from the Nielsen Company for England and Wales were published by NHS Health Scotland [11]. These data provide price distributions for alcohol sold in the off-trade. The Nielsen Company is unable to estimate off-trade sales by the low-priced supermarkets Aldi and Lidl from September 2011, and therefore the off-trade price distributions for 2011 are based on off-trade sales excluding these stores [11]. The impact of excluding Aldi and Lidl on off-trade price distributions in Scotland using 2009 and 2010 data was examined and only a marginal impact on the overall off-trade price distribution was detected [11]. Data is available for Great Britain and can also be partitioned for England and Wales. Data for England in isolation is not available hence data from England and Wales was used for the analysis.

Nielsen collects data from off-trade stores across the UK on a weekly basis. They maintain an extremely detailed dataset over the most recent three years. As each new week of data becomes available, the three year period is redefined and data older than three years is discarded. Whilst the detailed data provides a wealth of material, Nielsen does not provide any demographic data on purchasers (eg. no age/sex data), nor does it provide any direct information on actual consumption (as distinct from purchase) of alcohol.

For the database known as *Grocery Multiples channel*, which is essentially supermarket chains, sales data is stored at ‘stock keeping unit (SKU) level’. An SKU would, for example, be a 4-pack of 440ml cans of Carling and is defined by a unique bar-code. To protect the anonymity of individual brand data, Nielsen are unable to provide data at SKU level. However they are able to group the SKUs into 32 product types. The Nielsen data on a particular SKU for alcoholic beverages include the following fields: SKU code, week, store/outlet (at individual store level), volume of sales (in litres of beverage – Nielsen are unable to convert to units of alcohol using ABV), value of sales (in £), and product category.

The model performs analysis at the aggregated level of beer, cider, wine, spirits, and RTD, requiring further aggregation of the Nielsen product categories. The aggregation requires a transformation from litres of beverage to units of alcohol. This is achieved by applying ABV estimates, provided by Nielsen, to the volume of the product to obtain ethanol quantity and then converting to units.

For SKU anonymity reasons, Nielsen limited the number of categories of price range for which data was to be summarised to 17. These were defined at product level in terms of

price per litre of beverage, with the price ranges selected such that each category mapped back to an equivalent price per unit of alcohol (see Table 4.2).

Table 4.2: Price ranges for the Nielsen data

**Price category**	**Off-trade price (£) per unit of alcohol**	
	**Lower**	**Upper**
1	0.00	0.10
2	0.10	0.15
3	0.15	0.20
4	0.20	0.25
5	0.25	0.30
6	0.30	0.35
7	0.35	0.40
8	0.40	0.45
9	0.45	0.50
10	0.50	0.55
11	0.55	0.60
12	0.60	0.65
13	0.65	0.70
14	0.70	0.75
15	0.75	0.80
16	0.80	0.85
17	0.85	N/A

CGA Strategy, a market research company specialising in on-trade information, maintain a database of prices for beer/cider, wine, spirits, and RTD purchases in the on-trade. CGA Strategy data for England and Wales in 2011 was used to adjust the LCF/EFS on-trade prices. The CGA data was purchased by the Home Office and, although the detailed dataset is not publicly available, the University of Sheffield is permitted to use the data for SAPM.

CGA Strategy's pricing database for the on-trade (known as *Ons Prices*) records price information for products in a sample of outlets. The outlets in the sample are selected to be representative of the entire on-trade universe. Unique products are defined by brand and method of serve (eg. for beer, a product could be a 4 pint jug of draught Carling or a 330ml bottle of Becks).

To construct a price distribution, sales volumes (in terms of alcohol units) are required. Unfortunately CGA's pricing database does not include data of this type. However, a separate sales database (known as *Managed House EPoS Pricing Data Pool*) does record

total daily sales value (in £) and sales volume (in litres of beverage) for a sample of outlets. For most products, ABV information is also recorded, enabling volume to be converted to units of alcohol.

4.3 ACCOUNTING FOR PRICE INFLATION

Alcohol-specific RPIs for off- and on-trade beer - including cider - and off- and on-trade wine and spirits - including RTDs - (see Table 4.3) were used to adjust to 2011 prices the data in the LCF/EFS 2001/2 to 2009 [12,13]. The 2011 prices could then be aligned with the more accurate but more aggregated sales data from the Nielsen Company data and CGA Strategy data using the methods described in Section 4.4. These Neilsen/CGA aligned prices were then inflated to baseline year prices, again using alcohol-specific RPIs (Table 4.3). In this analysis 2014 is the baseline year for prices.

Table 4.3: ONS alcohol-specific RPIs 2001 to 2014

Year	Beer on-trade	Beer off-trade	Wine & spirits on-trade	Wine & spirits off-trade
2001	215.6	161.6	203.3	152.3
2002	221.7	160.7	210.6	153.3
2003	228.3	157.8	217.5	153.7
2004	234.9	153.5	223.0	155.0
2005	242.8	148.3	228.5	155.6
2006	251.1	147.8	235.4	156.5
2007	261.0	148.9	243.3	158.4
2008	272.4	149.0	253.1	165.2
2009	281.4	153.6	261.9	173.2
2010	291.8	155.4	271.5	180.4
2011	307.8	163.9	287.2	191.8
2012	318.1	169.7	298.9	196.2
2013	327.5	174.7	307.7	202.0
2014	337.1	179.9	316.8	207.9

4.4 ADJUSTING LCF/EFS DATA USING NIELSEN AND CGA DATA

The LCF/EFS provides the basis for the price distributions (comprised of individual transactions, defined by purchase price, purchase volume and sample weight). In the off-trade, the more aggregated but more accurate Nielsen data is used to adjust the LCF/EFS cumulative distribution so that it matches the Nielsen data at the known price points. The CGA data is used in the same manner for the on-trade data.

The LCF/EFS data is linearly interpolated between the known market research price points (retaining the maximum and minimum LCF/EFS prices as the boundaries of the distribution). Figure 4.1 illustrates the impact of these adjustments by beverage type and overall.

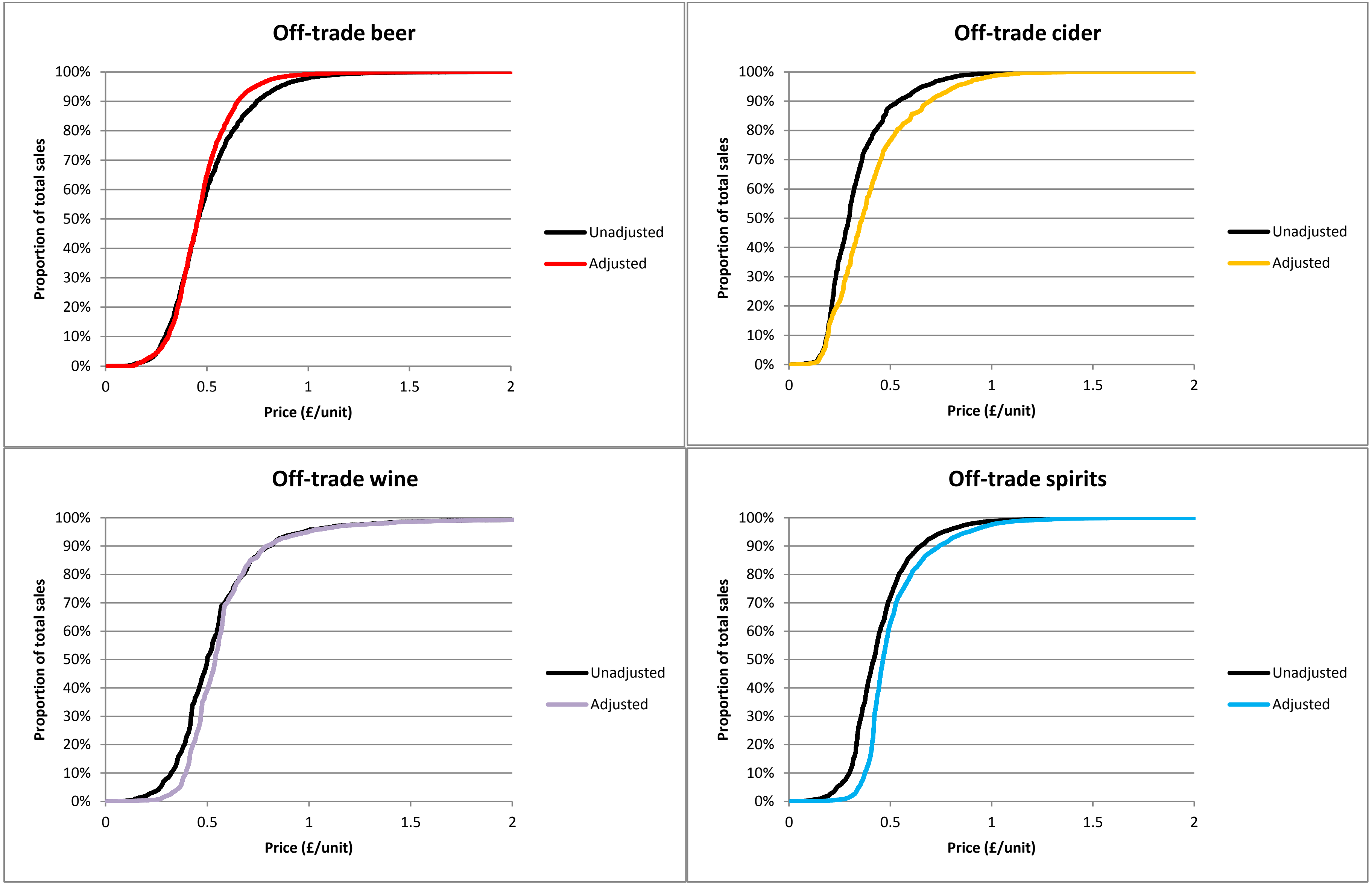
Off-trade beer
Proportion of total sales
100%
90%
80%
70%
60%
50%
40%
30%
20%
10%
0%
0
0.5
1
1.5
2
Price (£/unit)
Unadjusted
Adjusted
Off-trade cider
Proportion of total sales
Price (£/unit)
Unadjusted
Adjusted
Off-trade wine
Proportion of total sales
Price (£/unit)
Unadjusted
Adjusted
Off-trade spirits
Proportion of total sales
Price (£/unit)
Unadjusted
Adjusted

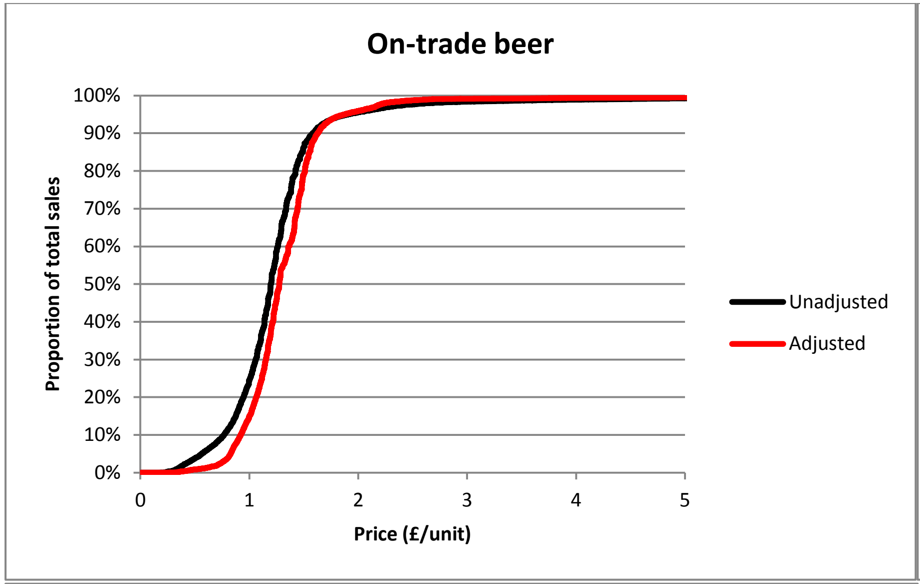
On-trade beer
100%
90%
80%
70%
60%
50%
40%
30%
20%
10%
0%
Proportion of total sales
0
1
2
3
4
5
Price (£/unit)
Unadjusted
Adjusted

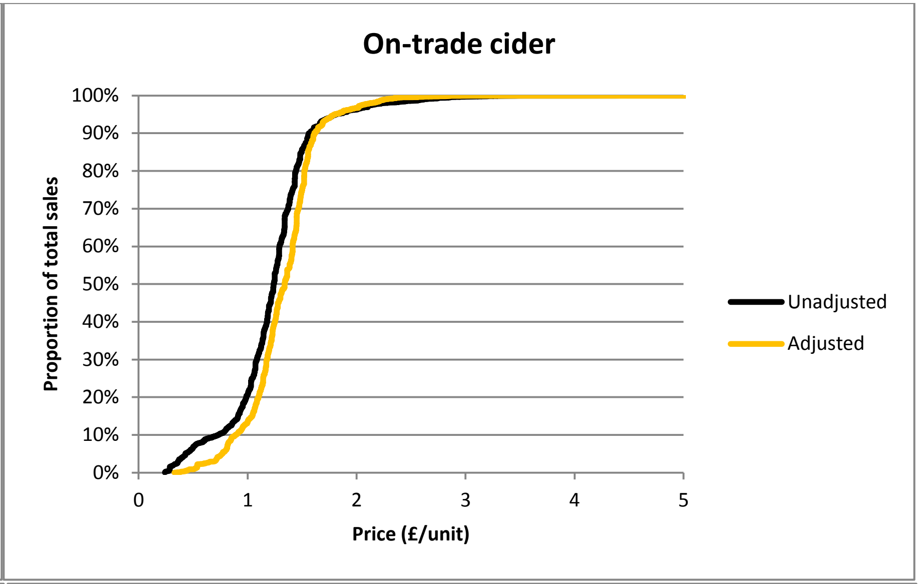
On-trade cider
100%
90%
80%
70%
60%
50%
40%
30%
20%
10%
0%
Proportion of total sales
0
1
2
3
4
5
Price (£/unit)
Unadjusted
Adjusted

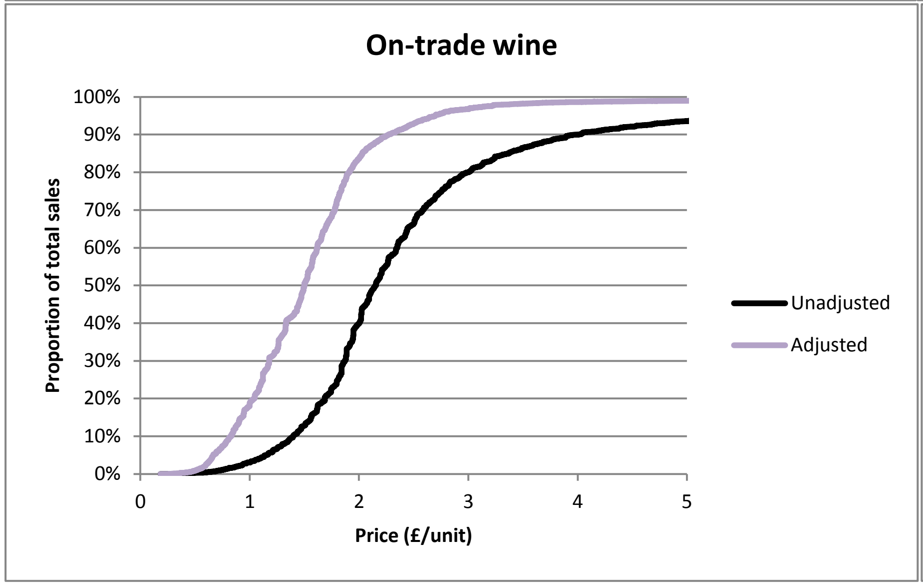
On-trade wine
100%
90%
80%
70%
60%
50%
40%
30%
20%
10%
0%
Proportion of total sales
0
1
2
3
4
5
Price (£/unit)
Unadjusted
Adjusted

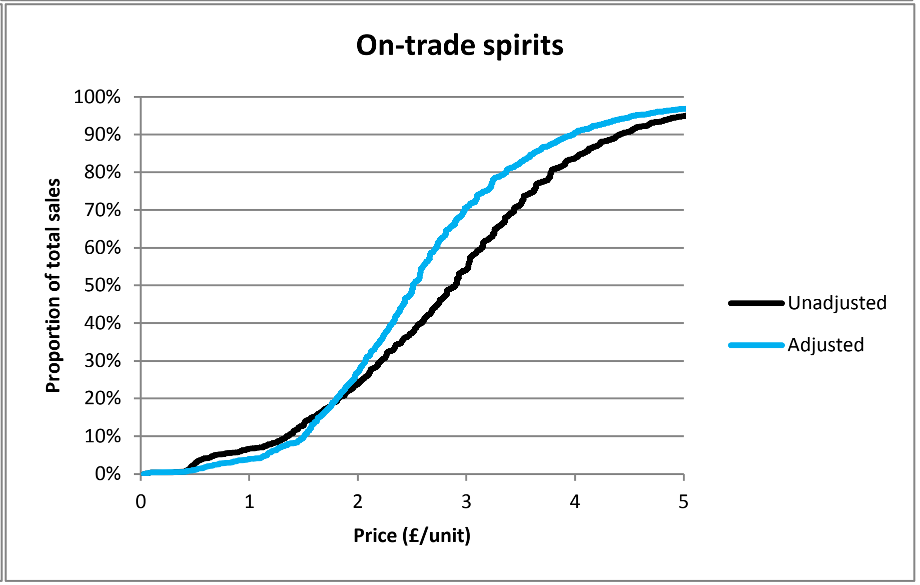
On-trade spirits
100%
90%
80%
70%
60%
50%
40%
30%
20%
10%
0%
Proportion of total sales
0
1
2
3
4
5
Price (£/unit)
Unadjusted
Adjusted

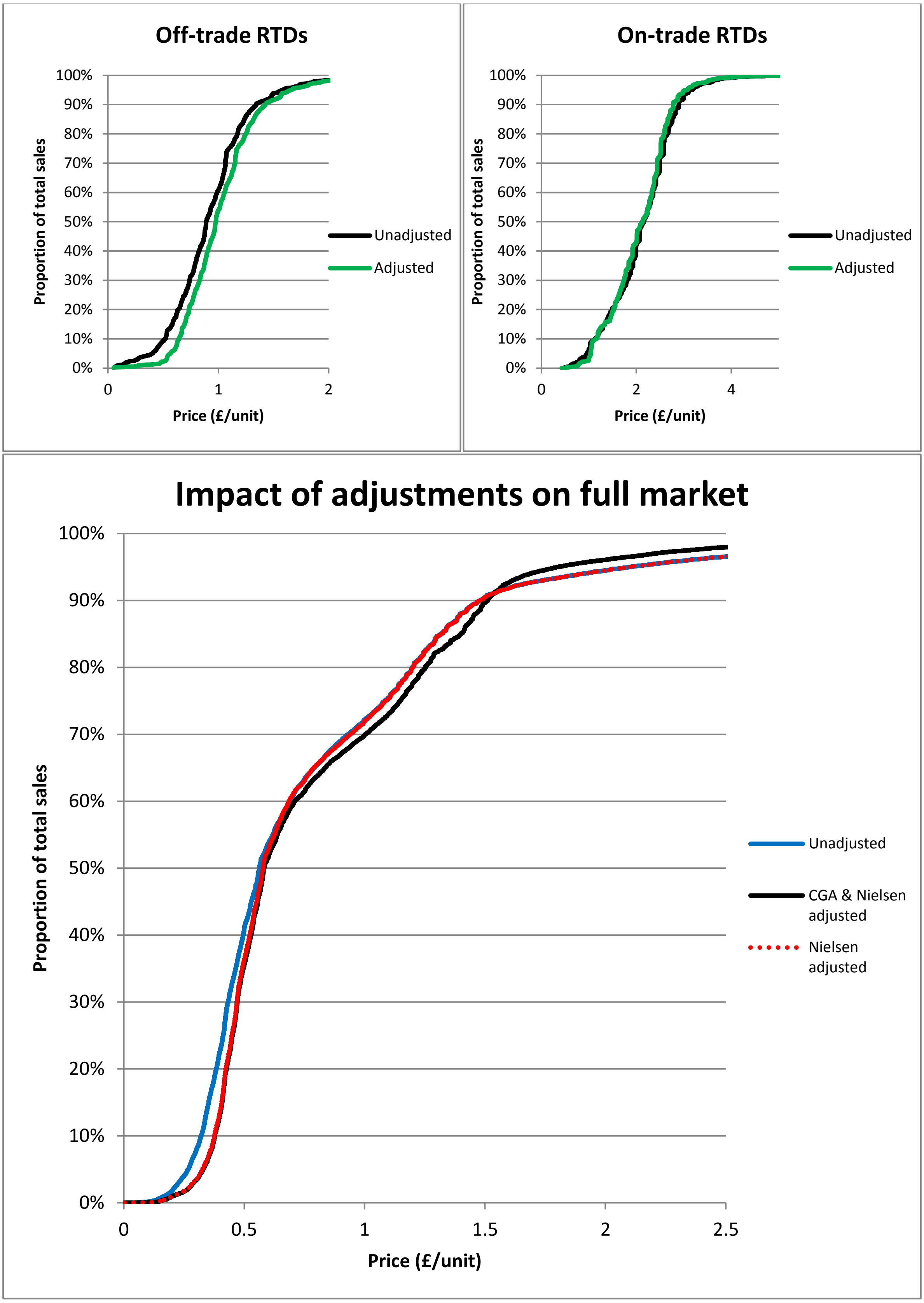

Figure 4.1: Impact of Nielsen and CGA adjustments on beverage-specific and overall price distributions

4.5 ESTIMATING BEVERAGE PREFERENCES

For each individual HSE respondent their preferences for beer (including cider), wine (including sherry), spirits and RTDs are captured by the beverage-specific quantity-frequency questions which are asked in the survey. Beer and cider are then disaggregated using the subgroup level combined LCF/EFS, Nielsen and CGA purchasing data for that subgroup. On- and off-trade preferences for each beverage are similarly disaggregated using the same combined LCF/EFS, Nielsen and CGA data. This produces a 10-element beverage preference vector for each individual.

The beverage preference vector for each HSE respondent can then be applied to the mean weekly alcohol consumption recorded in the HSE for the same individual to derive consumption estimates for the 10 modelled beverage types (off- and on- trade beer, cider, wine, spirits, RTDs) for each HSE respondent.

5 PRICE ELASTICITIES OF ALCOHOL DEMAND

SAPM3 uses the econometric model developed to estimate price elasticities of demand for alcohol which has been used from SAPM2.5 onwards [14].

The econometric model applies a pseudo-panel approach to the cross-sectional LCF/EFS 2001/2–2009 datasets to estimate the own- and cross- price elasticities of off- and on- trade beer, cider, wine, spirits and ready-to-drinks in the UK. 72 pseudo-panels were constructed defined by birth year (12), sex (2) and socioeconomic status (3). Fixed effects models were applied to analyse the pseudo-panel data.

Details of the econometric model have been published in the Journal of Health Economics [15]. The paper describes the rationale, method, data, results and limitations of the econometric analysis; and it forms an essential accompaniment to this technical appendix. Table 5.1 summaries the key result of the econometric analysis as a 10x10 elasticity matrix, with values on the diagonal representing own-price elasticities and remaining values representing cross-price elasticities. Elasticities are available for the 10 modelled categories of beverage: on- and off- trade beer, cider, wine, spirits, and RTDs.

As a simple example of how to interpret the elasticity matrix, consider Table 5.1. The lead diagonal shown in bold in the table contains the own-price elasticities. For example, the table shows an own-price elasticity of -0.980 for off-trade beer, indicating that a 1% increase in the price of off-trade beer would lead to an approximately 0.98% reduction in the demand for this beverage. Complement and substitute relationships between beverages are also indicated by the cross-price elasticities that comprise the remainder of the matrix. The majority of cross-price effects are of a substitute-based nature. For example, the cross-price elasticity between off-trade beer and off-trade wine is indicating an estimated 0.096% rise in demand for off-trade wine if the price of off-trade beer were to rise by 1%.

Table 5.1: Estimated own- and cross-price elasticities for off- and on-trade beer, cider, wine, spirits and RTDs in the UK

		Purchase									
		Off-beer	Off-cider	Off-wine	Off-spirits	Off-RTD	On-beer	On-cider	On-wine	On-spirits	On-RTD
Price	Off-beer	-0.980*	-0.189	0.096	-0.368	-1.092	-0.016	-0.050	0.253	0.030	0.503
	Off-cider	0.065	-1.268*	0.118	-0.122	-0.239	-0.053	0.093	0.067	-0.108	-0.194
	Off-wine	-0.040	0.736*	-0.384*	0.363	0.039	-0.245	-0.155	0.043	-0.186	0.110
	Off-spirits	0.113	-0.024	0.163	-0.082	-0.042	0.167	0.406	0.005	0.084	0.233
	Off-RTD	-0.047	-0.159	-0.006	0.079	-0.585*	-0.061	0.067	0.068	-0.179*	0.093
	On-beer	0.148	-0.285	0.115	-0.028	0.803	-0.786*	0.867	1.042*	1.169*	-0.117
	On-cider	-0.100	0.071	0.043	0.021	0.365	0.035	-0.591*	0.072	0.237*	0.241
	On-wine	-0.197	0.094	-0.154	-0.031	-0.093	-0.276	-0.031	-0.871*	-0.021	-0.363
	On-spirits	0.019	-0.117	-0.027	-0.280	-0.145	-0.002	-0.284	0.109	-0.890*	0.809*
	On-RTD	0.079	0.005	-0.085	-0.047	0.369	0.121	-0.394	-0.027	-0.071	-0.187

Remarks *: p-value <0.05

6 MODELLING THE IMPACT OF PRICING POLICIES ON ALCOHOL PRICES

Pricing policies are operationalised in SAPM3 through modifying the price paid in each transaction recorded in the LCF (see Section 4). A revised set of price distributions is then calculated. The mean of each revised price distribution is compared to the mean of the baseline price distribution to derive each population subgroup’s vector of percentage price changes which is the main input to the econometric model described in Section 5.

6.1 MODELLING THE UK BASELINE TAX SYSTEM

The baseline tax system used in all analyses is the duty and value added tax (VAT) system in England on 24th March (2014) and duties are shown in Table 6.1 [16]. In addition to alcohol duty, all alcoholic products are subject to general value added taxation levied at 20% of product price including excise duty. As shown in Table 6.1, duty structures vary by beverage type such that tax for wine and cider is proportionate to the volume of product, whilst beer, spirits and spirits-based drinks (including RTDs) are taxed proportionate to the volume of ethanol. Each beverage category is also taxed at a different level with spirits taxed at the highest rate per unit of alcohol, followed by wine and then beer while cider in its most common form has a notably low tax rate. There are alcoholic strength-based thresholds for all beverage categories except spirits and also differentiations made between still and sparkling varieties of wine and cider. In addition, a floor price is in place which prohibits the sale of alcoholic drinks below the cost of the duty and VAT payable on the product.

A number of assumptions are made when modelling the tax system. As the market shares of sparkling wines, ciders and perries are very small, we assume the standard duty rate for these products [17]. For the same reason, the standard duty rate is also assumed for beverages falling within higher and lower strength tax bands. For wine and cider, where duty is calculated based on volume of product, average ABV content of wines and ciders sold in the UK were obtained from, the market research company, Nielsen and used to calculate the duty per unit levels required by the analysis. These values are 12.58% for wine and 4.9% for cider. This means duty per unit of wine and cider remains constant irrespective of ABV which is not the case in reality. However, variations in wine ABV are modest and cider accounts for less than 10% of total consumption despite being an important product category for some dependent drinkers [18]. Finally, SAPM3 does not incorporate the ban on selling alcohol below the price of the duty and VAT payable as previous analyses have

shown the effect of this ban to be negligible [19].The resulting baseline duty rates as modelled by SAPM3 are shown in Table 6.2.

Table 6.1: Alcohol duty rates in the UK from 24th March 2014

Beverage	ABV range	Method of taxation	Rate
Spirits	N/A	Per litre of pure alcohol	£28.22
Spirits-based ready-to-drinks	N/A		
Wine and made-wine	>22%		
Beer (General)	≥1.2% - ≤7.5%	Per hectolitre per cent of alcohol in the beer	£18.74
Beer (High strength)	>7.5%		£5.29
Beer (Lower strength)	>1.2% - ≤2.8%		£8.62
Still cider and perry	>1.2% - ≤7.5%	Per hectolitre of product	£39.66
Still cider and perry	>7.5% - <8.5%		£59.52
Sparkling cider and perry[1]	>1.2% - ≤5.5%		£39.66
Sparkling cider and perry[1]	>5.5% - <8.5%		£246.61
Wine and made-wine	>1.2% - ≤4.0%		£84.21
Wine and made-wine	>4% - ≤15%		£273.31
Wine and made-wine	>15% - ≤22%		£364.37
Sparkling wine and made-wine	>5.5% - ≤8.5%		£264.61
Sparkling wine and made-wine	≥8.5% - ≤15%		£350.07

[1]Sparkling cider and perry is defined for duty purposes as being in a closed bottle with excess pressure, due to carbon dioxide, of three bars or more at 20 degrees Celsius or, regardless of pressure, being contained in a closed bottle with a mushroom shaped stopper held in place by a tie or fastening [20]. In practice, most ciders which appear 'sparkling' to consumers are taxed as 'still' due to this definition.

Table 6.2: Modelled duty rates by beverage type

Beverage	Modelled duty rate per unit[1]	Modelled duty rate per unit including VAT[1]
Beer	£0.19	£0.22
Cider (average ABV: 4.90%)	£0.08	£0.10
Wine (average ABV: 12.58%)	£0.22	£0.26
Spirits	£0.28	£0.34
RTDs	£0.28	£0.34

[1] All figures rounded to nearest pence.

6.2 MODELLING DIFFERENTIAL PASS-THROUGH OF TAX INCREASES BY PRODUCT AND BASELINE PRICE

A recent analysis [21] of the extent to which alcohol duty and VAT changes lead to changes in the price of alcoholic products in UK supermarkets showed variation in 'pass-through' of tax changes by beverage type and baseline price. In brief, the analyses used weekly data on prices charged for a panel of 254 alcoholic products sold in four major UK supermarkets between March 2008 and August 2011. The results suggested taxes are not fully passed-through for cheaper products meaning prices changed by less than implied by the tax change. For more expensive products the reverse was true and prices changed by more than implied by the tax change. Although the general pattern of pass-through was consistent across beverage types, the scale of the effect and the point in the price distribution at which pass-through shifted from less to more than expected varied by beverage type.

SAPM3 incorporates this evidence when modelling policies affecting baseline tax rates. Further adjustments are made to the revised transaction-level prices paid to reflect tax pass-through rates. Table 6.3 summarises the adjustment factors (pass-through rates) by beverage and for price per unit bands within beverages. The price bands are quantiles of the price distribution for the panel of products.

These adjustments are only made for off-trade beverages as no evidence was available on pass-through rates in the on-trade and it cannot be assumed on-trade and off-trade pass-through rates are similar given the typical difference in number of products sold in on-trade outlets and business model.

Table 6.3: Pass-through adjustment factors by price band and beverage

**Beer**		**Cider/RTDs**		**Spirits**		**Wine**	
Price per unit	Pass-through adjustment factor	Price per unit	Pass-through adjustment factor	Price per unit	Pass-through adjustment factor	Price per unit	Pass-through adjustment factor
0.466<	0.852	0.287<	0.889	0.358<	0.86	0.377<	0.908
(0.466, 0.531]	0.971	(0.287, 0.31]	1.012	(0.358, 0.409]	0.942	(0.377, 0.436]	1.06
(0.531, 0.58]	1.03	(0.31, 0.469]	1.05	(0.409, 0.488]	1.048	(0.436, 0.479]	1.099
(0.58, 0.62]	1.042	(0.469, 0.676]	1.056	(0.488, 0.568]	1.069	(0.479, 0.533]	1.108
(0.62, 0.651]	1.053	(0.676, 0.777]	1.06	(0.568, 0.62]	1.082	(0.533, 0.56]	1.109
(0.651, 0.668]	1.06	(0.777, 0.851]	1.065	(0.62, 0.661]	1.087	(0.56, 0.583]	1.113
(0.668, 0.703]	1.063	(0.851, 0.91]	1.067	(0.661, 0.746]	1.089	(0.583, 0.616]	1.113
(0.703, 0.728]	1.072	(0.91, 0.958]	1.076	(0.746, 0.856]	1.093	(0.616, 0.689]	1.12
(0.728, 0.776]	1.077	(0.958, 0.992]	1.082	(0.856, 0.961]	1.096	(0.689, 0.754]	1.127
(0.776, 0.849]	1.103	(0.992, 1.102]	1.109	(0.961, 1.135]	1.11	(0.754, 0.915]	1.147
>0.849	1.139	>1.102	1.185	>1.135	1.126	>0.915	1.18

7 PRICE TO CONSUMPTION MODEL

7.1 MODEL OVERVIEW

Data from the HSE 2012 were used to provide the baseline data for alcohol consumption in England. The main mechanism of the price to consumption model is that a change in price modifies the consumption patterns derived from the HSE. Within the model, a new HSE is simulated for each modelled year based on the estimated impact of the policy which is being appraised. However, the HSE does not provide information about on- and off-trade consumption, nor does it differentiate between beer and cider consumption. These are critical additional components required to model the impact of policies with differential impacts on prices by beverage type, and by the on- and off-trade. Thus the baseline HSE needs to be augmented using the LCF so that beer and cider consumption can be disaggregated and the on- versus off- trade distinction can be properly accommodated in the model.

The price to consumption model is therefore composed of three major steps (Figure 7.1):

1. The LCF is used to derive a “new HSE” containing consumption estimates for 10 beverage types; off- and on- trade beer, cider, wine, spirits, RTDs (described in Section 4.5).
2. The LCF is adjusted using Nielsen and CGA data (described in Section 4.4).
3. The model is then used to estimate the impact of a proposed policy change in terms of change in consumption.

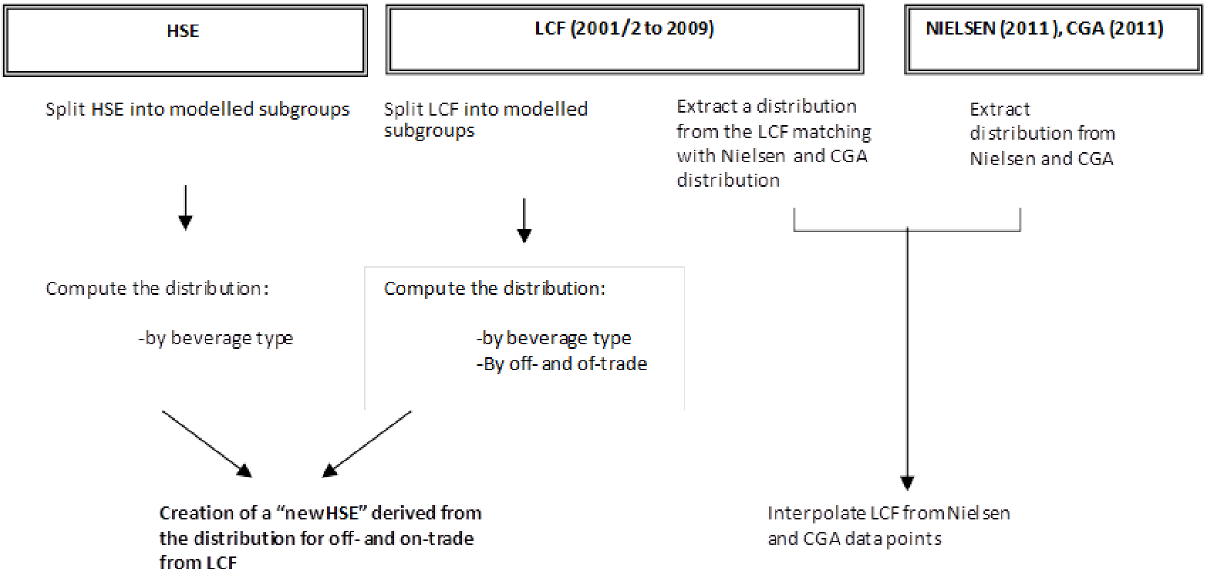

Figure 7.1: Model construction steps: creation of a "new HSE" and Nielsen/CGA adjusted LCF/EFS dataset

Step 3 estimates the impact of a price policy on mean weekly consumption for each modelled subgroup, taken from the "new HSE", using the elasticity matrix described in Table 5.1. The formula used to apply the elasticity matrix is shown below:

$$\%\Delta C_i = (1 + e_{ii}\%\Delta p_i)(1 + \sum_{j \neq i}^{\forall j} e_{ij}\%\Delta p_j) - 1 \qquad \textit{Equation 1}$$

Where, $\%\Delta C_i$ is the estimated percentage change in consumption for beverage *i*, e_{ii} is the own-price elasticity for beverage *i*, $\%\Delta p_i$ is the percentage change in price for beverage *i*, e_{ij} is the cross-price elasticities for the consumption of beverage *i* due to a change in the price of beverage *j*, and $\%\Delta p_j$ is the percentage change in price for beverage *j*.

As described in Sections 3.4 and 3.5, the estimated relative change in weekly consumption for each subgroup is then used to predict the relative change in peak daily consumption, the number of drinking occasions per week and variation in consumption on those occasions for that subgroup.

7.2 MODELLING THE 'NO POLICY' SCENARIO

In order to estimate the impact of any modelled policy on alcohol consumption and subsequent health outcomes it is necessary not only to model the future alcohol consumption of the population under the policy, but to model the counterfactual scenario in which no policy is implemented. SAPM is constructed on the assumption that, in this scenario, alcohol consumption remains unchanged in the population. That is to say that the

40 year olds of the future drink the same as the 40 year olds of the present. This assumption is unlikely to hold true in practice, as we know that there are both period effects – changes in consumption over time driven by temporal trends in the whole population – and cohort effects – trends across successive generations – in both rates of abstention from drinking and levels of drinking among drinkers have been observed in Great Britain over the past 25 years [22] However, these trends are volatile and inherently difficult to forecast and, as a result the assumption that consumption (and implicitly abstention) will remain static is made as it is both simple and transparent.

It should be noted that this assumption underpins both ‘arms’ of the model (i.e. the policy and no policy scenarios) as the intervention arm models consumption changing only as a result of the policy, rather than any additional period or cohort effects.

8 CONSUMPTION TO HEALTH HARMS MODEL

8.1 HEALTH MODEL STRUCTURE

The model aims to capture the policy impact for the large number of health conditions for which evidence suggests alcohol plays a contributory role. Table 8.1 presents a list of all included conditions, which has been adapted from recent global meta-analyses and burden of disease studies [23,24]. These conditions are divided into four categories of attribution:

1. Wholly attributable, chronic – meaning that the harm cannot occur in the absence of alcohol consumption, and risk of occurrence changes with chronic exposure to alcohol (e.g. alcoholic liver disease, ICD-10 code = K70).
2. Wholly attributable, acute – meaning that the harm cannot occur without alcohol as its cause, and risk of occurrence changes with acute exposure to alcohol including intoxication (e.g. Ethanol poisoning, ICD-10 code = T51.0).
3. Partially attributable, chronic – meaning that the harm can occur without alcohol but the risk of occurrence changes with chronic exposure to alcohol (e.g. malignant neoplasm (cancer) of the oesophagus, ICD-10 code = C15). There are three conditions within this category – ischaemic heart disease, ischaemic stroke and type II diabetes – in which alcohol may have an overall protection effect.
4. Partially attributable, acute – meaning that the harm can occur without alcohol but the risk of occurrence changes with acute exposure to alcohol (e.g. falls, ICD-10 code = W00-W19, or assault, ICD-10 = X85-Y09).

Table 8.1: Health conditions included in the model

Category	Disease or injury	ICD-10 codes	Source for risk function
Wholly attributable to alcohol, chronic (10)	Alcohol-induced pseudo-Cushing's syndrome	E24.4	
	Degeneration of nervous system due to alcohol	G31.2	
	Alcoholic polyneuropathy	G62.1	
	Alcoholic myopathy	G72.1	
	Alcoholic cardiomyopathy	I42.6	
	Alcoholic gastritis	K29.2	
	Alcoholic liver disease	K70.0-K70.4, K70.9	
	Acute pancreatitis (alcohol induced)	K85.2	
	Chronic pancreatitis (alcohol induced)	K86.0	
	Maternal care for (suspected) damage to foetus from alcohol	O35.4	
Wholly attributable to alcohol, acute (7)	Mental and behavioural disorders due to use of alcohol	F10	
	Excessive Blood Level of Alcohol	R78.0	
	Toxic effect of alcohol	T51.0, T51.1, T51.8, T51.9	
	Accidental poisoning by exposure to alcohol	X45	
	Intentional self-poisoning by and exposure to alcohol	X65	
	Poisoning by and exposure to alcohol, undetermined intent	Y15	
	Evidence of alcohol involvement determined by blood alcohol level	Y90	
Partially attributable to alcohol, chronic (14)	Tuberculosis	A15-A19, B90	Lonnroth *et al* 2008 [25]
	Malignant neoplasm of lip, oral cavity and pharynx	C00-C14	Tramacere *et al* 2010 [26]
	Malignant neoplasm of oesophagus	C15	Rota *et al* 2009 [27]
	Malignant neoplasm of colon and rectum	C18-C21	Fedirko *et al* 2011 [28]
	Malignant neoplasm of liver and intrahepatic bile ducts	C22	Corrao *et al* 2004 [29]
	Malignant neoplasm of larynx	C32	Islami *et al* 2011 [30]
	Malignant neoplasm of breast	C50	Key *et al* 2006 [31]
	Epilepsy and status epilepticus	G40-G41	Samokhvalov *et al* 2010 [32]
	Hypertensive diseases	I10-I14	Taylor *et al* 2009 [33]
	Cardiac arrhythmias	I47-I48	Kodama *et al* 2011 [34]
	Haemorrhagic and other non-ischaemic stroke	I60-I62, I69.0-I69.2	Patra *et al* 2010 [35]
	Lower respiratory infections: pneumonia	J09-J22, J85, P23	Samokhvalov *et al* 2010 [36]
	Cirrhosis of the liver (excluding alcoholic liver disease)	K70 (excl. K70.0-K70.4, K70.9), K73-K74	Rehm *et al* 2010 [37]
	Acute and chronic pancreatitis	K85-K86 excl. K85.2, K86.0	Irving *et al* 2009 [38]
Partially attributable to alcohol, chronic, beneficial effect (3)	Diabetes mellitus (type II)	E10-E14	Baliunas *et al* 2009 [39]
	Ischaemic heart disease	I20-I25	Roerecke and Rehm 2012 [40][1]
	Ischaemic stroke	I63-I67, I69.3	Patra *et al* 2010 [35]
Partially attributable to alcohol, acute (9)	Transport injuries (including road traffic accidents)	V01-V98, Y85.0	Taylor *et al* 2011 [41][2]
	Fall injuries	W00-W19	
	Exposure to mechanical forces (including machinery accidents)	W20-W52	
	Drowning	W65-W74	
	Other Unintentional Injuries	W75-W99, X30-X33, X50-X58	
	Accidental poisoning by exposure to noxious substances	X40-X49 excl. X45	
	Intentional self-harm	X60-X84, Y87.0 excl. X65	
	Assault	X85-Y09, Y87.1	
	Other intentional injuries	Y35	

[1] See Section 9.3.1 for further details
[2] See Section 0 for further details

8.2 MORTALITY MODEL STRUCTURE

A simplified version of the model structure for mortality is presented in Figure 8.1. The model is developed to represent the population of England in a life table. Separate life tables have been implemented for males and females.

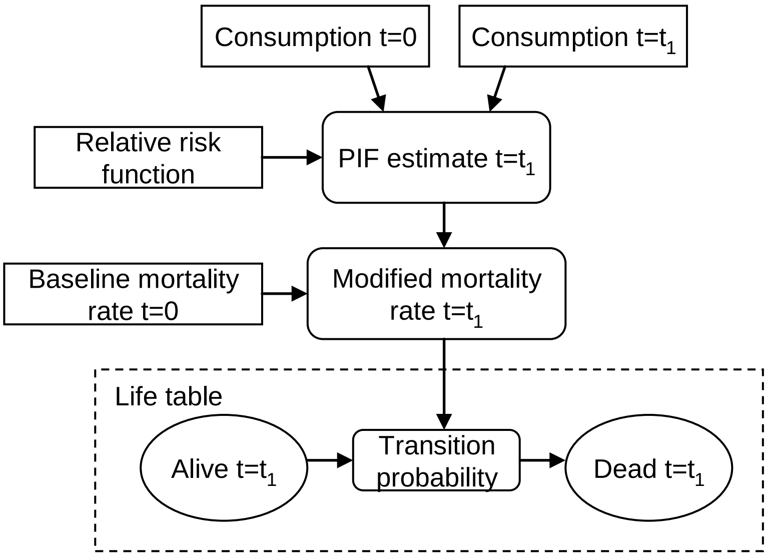

Figure 8.1: Simplified mortality model structure

The life table is implemented as a linked set of simple Markov models with individuals of age *a* transitioning between two states – alive and dead – at model time step *t*. Those of age *a* still alive after the transition then form the initial population for age *a+1* at time *t+1* and the sequence repeats.

The transition rates from the alive to dead state are broken down by condition and are individually modified via potential impact fractions over time *t*, where the PIF essentially varies with consumption (mean for chronic conditions and maximum daily for acute conditions) over time:

$$PIF_t = 1 - \frac{\sum_{i=1}^{N} w_i RR_{i,t}}{\sum_{i=1}^{N} w_i RR_{i,0}}$$ *Equation 2*

where PIF_t is the potential impact fraction relating to consumption at time *t*, *i* = HSE sample number, *N* = number of samples in subgroup *i*, $RR_{i,t}$ is the risk relating to the consumption of HSE sample *i* at time *t*, $RR_{i,0}$ is the risk at baseline, and w_i is the weight of sample *i*.

Note that the PIF can be decomposed to enable different population groups at baseline – for example, moderate, increasing risk and high risk drinkers – to be followed separately over the course of the model.

The model computes mortality results for two separate scenarios (a baseline – implemented as ‘no change to consumption’ in the analysis herein – and an intervention). The effect of the intervention is then calculated as the difference between the life tables of two scenarios: enabling the change in the total expected deaths attributable to alcohol due to the policy to be estimated.

9 APPROACH TO MODELLING THE RELATIONSHIP BETWEEN CONSUMPTION AND HARM

9.1 MODEL STRUCTURE

An epidemiological approach is used to model the relationship between consumption and harm, relating changes in the prevalence of alcohol consumption to changes in prevalence of risk of experiencing harmful outcomes. Risk functions relating consumption (however described) to level of risk are the fundamental components of the consumption to harm model.

The consumption to harm model considers the impact of consumption on harms in three domains: health (including the impact on both mortality and morbidity), crime and the workplace. As the present paper focusses only on mortality outcomes these are described below. Full details on the morbidity, crime and workplace aspects of SAPM3 can be found elsewhere [42–44]

9.2 ALCOHOL-ATTRIBUTABLE FRACTIONS AND POTENTIAL IMPACT FRACTIONS

The methodology is similar to that used in Gunning-Scheper's Prevent model [45], being based on the notion of the alcohol-attributable fraction (AAF) and its more general form, the potential impact fraction (PIF).

The AAF of a disease can be defined as the difference between the overall average risk (or incidence rate) of the disease in the entire population (drinkers and never-drinkers) and the average risk in those without the exposure factor under investigation (never-drinkers), expressed as a fraction of the overall average risk. For example, the AAF for female breast cancer is simply the risk of breast cancer in the total female population minus the risk of breast cancer in women who have never drunk alcohol, divided by the breast cancer risk for the total female population. Thus, AAFs are used as a measure of the proportion of the disease that is attributable to alcohol. While this approach has traditionally been used for chronic health-related outcomes, it can in principle be applied to other harms (including those outside of the health domain).

The AAF can be calculated using the following formula:

$$AF = \frac{\sum_{i=1}^{n} p_i \left(RR_i - 1\right)}{1 + \sum_{i=1}^{n} p_i \left(RR_i - 1\right)}$$

Equation 3

where, *RRi* is the relative risk (RR) due to exposure to alcohol at consumption state *i*, *pi* is the proportion of the population exposed to alcohol at consumption state *i*, and *n* is the number of consumption states.

If the reference category is abstention from alcohol then the AAF describes the proportion of outcomes that would not have occurred if everyone in the population had abstained from drinking. Thus the numerator is essentially the excess expected cases due to alcohol exposure and the denominator is the total expected cases. In situations where certain levels of alcohol consumption reduce the risk of an outcome (e.g. coronary heart disease) the AAF can be negative and would describe the additional cases that would have occurred if everyone was an abstainer.

Note that there are methodological difficulties with AAF studies. One problem is in defining the non-exposed group – in one sense ‘never drinkers’ are the only correct non-exposed group, but they are rare and usually quite different from the general population in various respects. However, current non-drinkers include those who were heavy drinkers in the past (and these remain a high-risk group, especially if they have given up due to alcohol-related health problems). Several studies show that findings of avoided coronary heart disease risk may be based on systematic errors in the way abstainers were defined in the underlying studies [46].

The PIF is a generalisation of the AAF based on arbitrary changes to the prevalence of alcohol consumption (rather than assuming all drinkers become abstainers). Note that a lag may exist between the exposure to alcohol and the resulting change in risk. The PIF can be calculated using the following formula:

$$PIF = 1 - \frac{\sum_{i=0}^{n} \overline{p_i} RR_i}{\sum_{i=0}^{n} p_i RR_i}$$

Equation 4

where $\overline{p_i}$ is the modified prevalence for consumption state *i* and state 0 corresponds to abstention.

In the model, alcohol consumption in a population subgroup is described non-parametrically by the associated observations from the HSE. For any harmful outcome, risk levels are associated with consumption level for each of the observations (note that these are not person-level risk functions). The associated prevalence for the observation is simply defined by its sample weight from the survey. Therefore, the PIF is implemented in the model as:

$$PIF = 1 - \frac{\sum_{i=0}^{N} w_i \overline{RR_i}}{\sum_{i=0}^{N} w_i RR_i} \qquad \textit{Equation 5}$$

where w_i is the weight for observation i, $\overline{RR_i}$ is the modified risk for the new consumption level and N is the number of samples.

9.3 DERIVATION OF RISK FUNCTIONS

The impact of a change in consumption on harm was examined using three categories of risk functions:

1. Relative risk functions already available in the published literature.
2. Relative risk functions derived from the AAF for partially attributable harms.
3. Absolute risk functions for wholly attributable harms.

9.3.1 Relative risk functions already available in the published literature

The relative risk functions for all chronic conditions that are partially attributable to alcohol are shown in Figure 9.1 and are taken from the published literature (see Table 8.1).

A number of recent studies have highlighted that the finding that some levels of alcohol consumption appear to have a protective effect for some cardiovascular health conditions, in particular Ischaemic Heart Disease (IHD), does not apply for drinkers who consume their alcohol on a small number of heavy episodic occasions (i.e. ‘binges’) rather than drinking smaller amounts more regularly [47–49]. Following an extensive review of this evidence, we operationalise this in SAPM3 by removing any protective effect for individuals whose mean consumption means that they must drink at least 60g of alcohol at least once per month. This approach is in line with that previously used elsewhere [50].

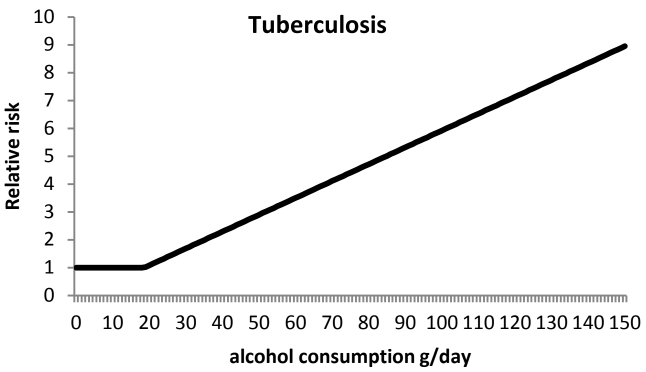
Tuberculosis
Relative risk
0 1 2 3 4 5 6 7 8 9 10
0 10 20 30 40 50 60 70 80 90 100 110 120 130 140 150
alcohol consumption g/day

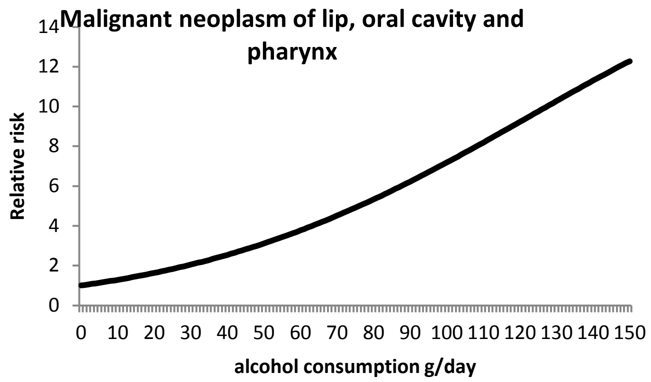
Malignant neoplasm of lip, oral cavity and pharynx
Relative risk
0 2 4 6 8 10 12 14
0 10 20 30 40 50 60 70 80 90 100 110 120 130 140 150
alcohol consumption g/day

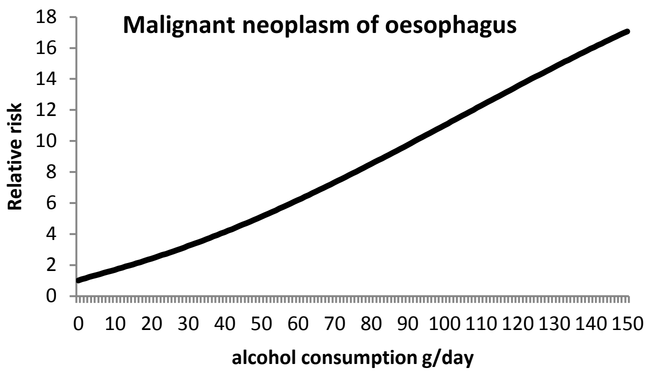
Malignant neoplasm of oesophagus
Relative risk
0 2 4 6 8 10 12 14 16 18
0 10 20 30 40 50 60 70 80 90 100 110 120 130 140 150
alcohol consumption g/day

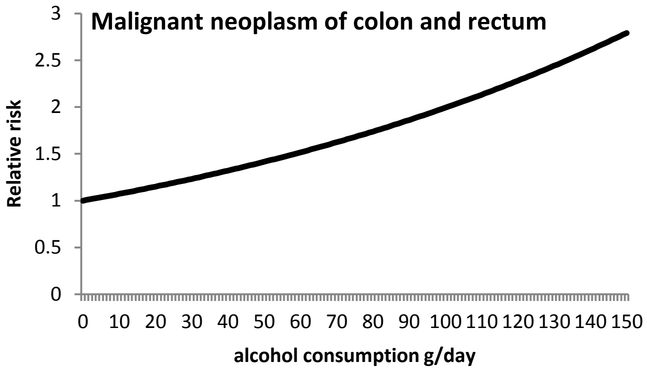
Malignant neoplasm of colon and rectum
Relative risk
0 0.5 1 1.5 2 2.5 3
0 10 20 30 40 50 60 70 80 90 100 110 120 130 140 150
alcohol consumption g/day

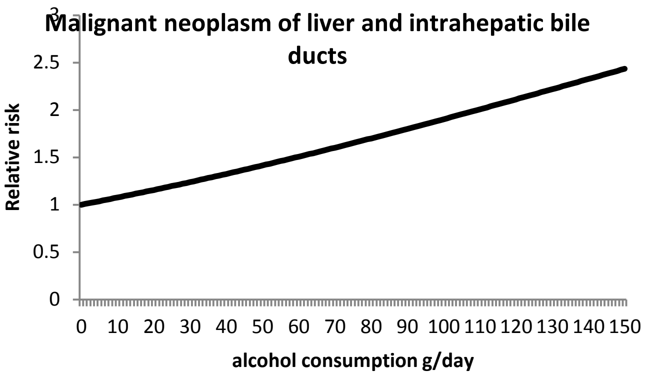
Malignant neoplasm of liver and intrahepatic bile ducts
Relative risk
0 0.5 1 1.5 2 2.5 3
0 10 20 30 40 50 60 70 80 90 100 110 120 130 140 150
alcohol consumption g/day

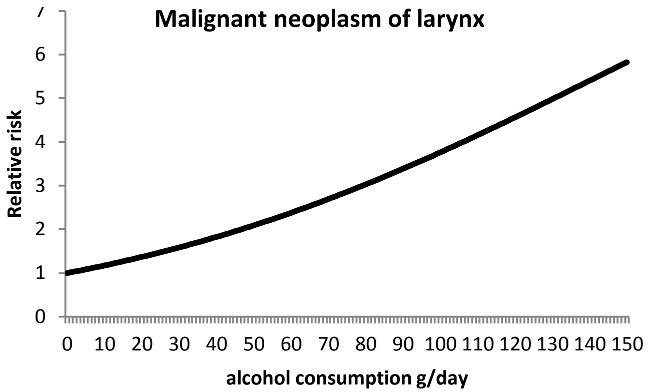
Malignant neoplasm of larynx
Relative risk
0 1 2 3 4 5 6 7
0 10 20 30 40 50 60 70 80 90 100 110 120 130 140 150
alcohol consumption g/day

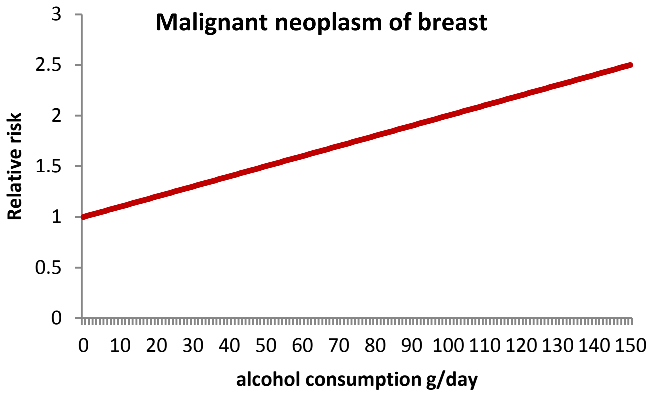
Malignant neoplasm of breast
Relative risk
0 0.5 1 1.5 2 2.5 3
0 10 20 30 40 50 60 70 80 90 100 110 120 130 140 150
alcohol consumption g/day

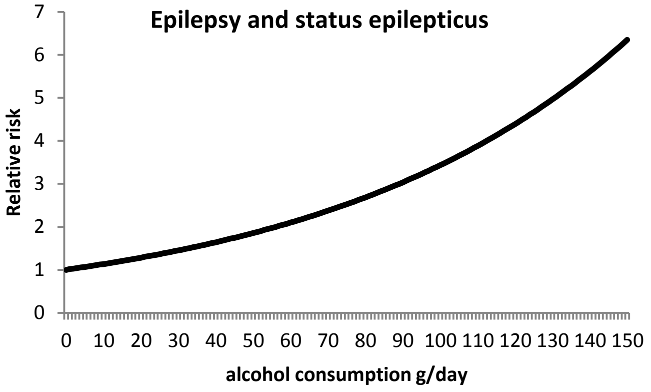
Epilepsy and status epilepticus
Relative risk
0 1 2 3 4 5 6 7
0 10 20 30 40 50 60 70 80 90 100 110 120 130 140 150
alcohol consumption g/day

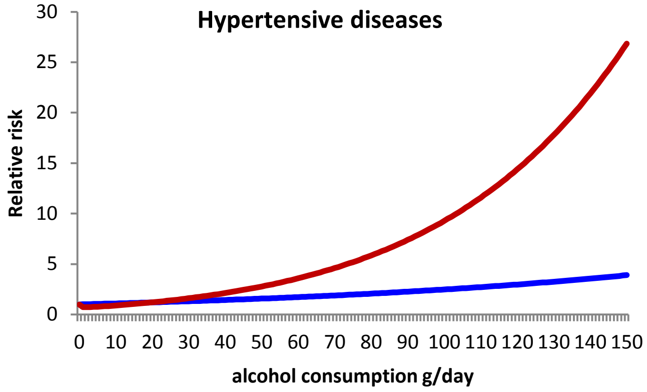
Hypertensive diseases
Relative risk
0 5 10 15 20 25 30
0 10 20 30 40 50 60 70 80 90 100 110 120 130 140 150
alcohol consumption g/day

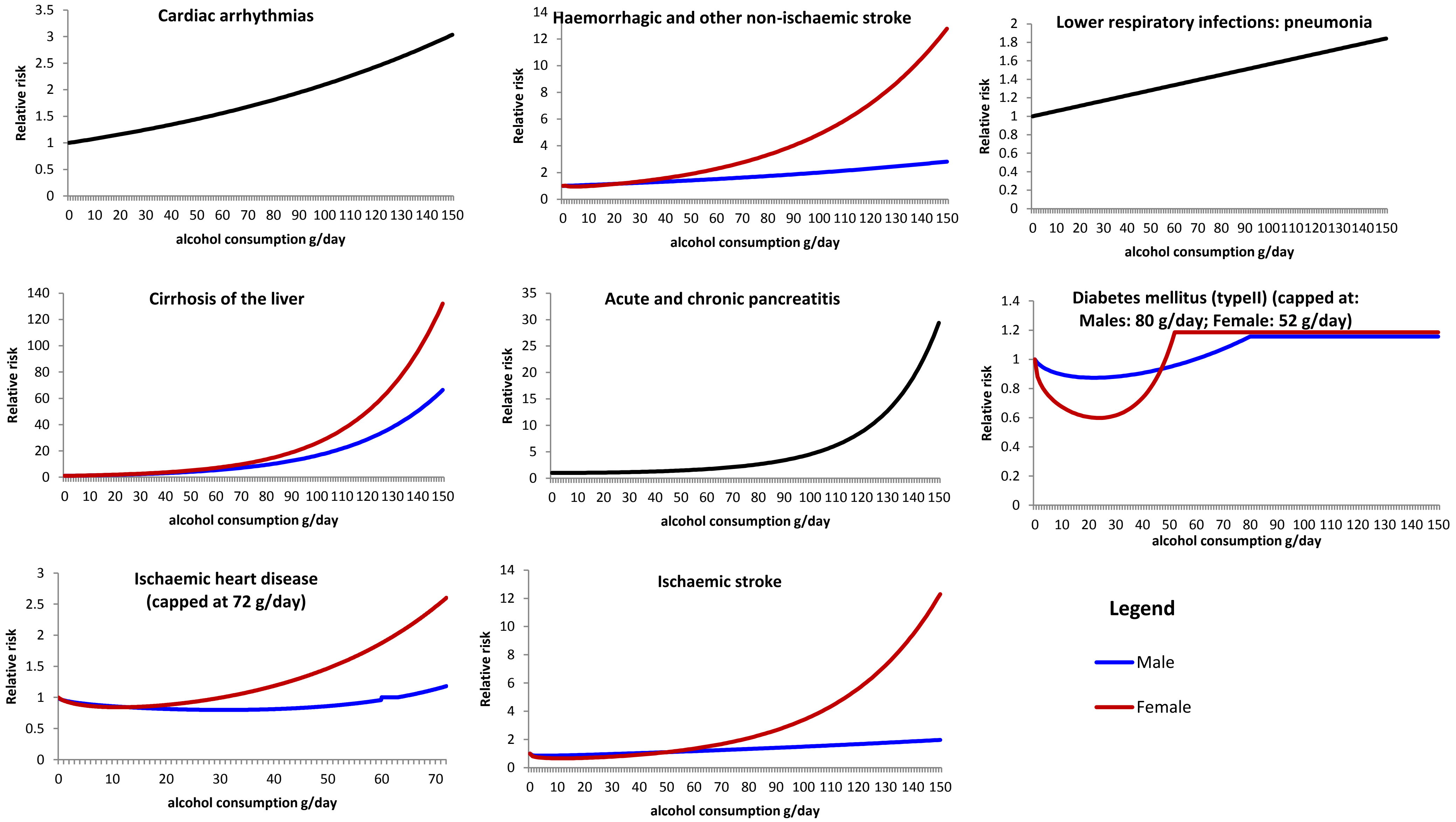

Figure 9.1: Relative Risk functions for partially alcohol-attributable chronic health conditions

9.3.2 Modelling partially attributable acute health harms

Partially attributable acute health harms include various traffic and non-traffic injuries. A new method of modelling risks associated with these harms was developed for SAPM3. Within the new method, the identified relative risk functions for these harms are different from the relative risk functions for partially attributable chronic conditions and cannot be used directly in Equation 2. The input and outcome of the relative risk functions for partially attributable chronic conditions are usual alcohol consumption and relative risk over a certain period of time; however, the input and outcome of the identified relative risk functions for traffic and non-traffic injuries are levels of drinking on the occasion prior to the injury and the relative risk for the drinking occasions [41]. As SAPM3 works on annual cycles, relative risk in Equation 2 is defined as annual relative risk. Therefore, to apply Equation 2, single drinking occasion based relative risk needs to be converted to long-term (i.e. annual) relative risk of a surveyed individual.

A new method to estimate annualised relative risk of alcohol-affected traffic and non-traffic injuries has been developed and is described fully elsewhere [8,51]. Briefly, the three measures described in Section 3.5 are defined to represent drinking patterns based on single drinking occasions. These are (1) the frequency of drinking occasions (drinking occasions per week); (2) the mean level of alcohol consumption for a given drinking occasion (units of alcohol) and (3) the variability of alcohol consumption for a given drinking occasion (standard deviation of units of alcohol consumed in drinking occasions). Using the estimated values for these parameters - derived using the methods in Section 3.5 - and the duration of intoxication for a given drinking occasions calculated by applying the Widmark equation [52,53], a series of integrations was performed to calculate the annualised relative risk for traffic and non-traffic accidents. The annualised relative risk is used in Equation 4 to estimate the potential impact factor for partially attributable acute health harms.

9.3.3 Absolute risk functions for wholly attributable harms

While it was possible to estimate relative risk functions for most harms, it was impossible to derive such functions for wholly alcohol-attributable harms (with an AAF of 100%) due to the absence of a reference group (as, by definition, abstainers have zero risk of suffering these harms).

An alternative approach was thus adopted: absolute risk functions were calculated based on the number of harm events, the drinking prevalence, and the total population. In order to do this, assumptions were necessary about the form and the starting threshold of the absolute risk functions.

For wholly attributable chronic harms the risk was assumed to start from 3 units per day for males and 2 units per day for females. These thresholds were derived from the Royal College of Physicians' (RCP) limits [54], (i.e. drinking less than 21 units per week for males and 14 units per week for females). Risk was not assumed to start from zero units, since it was thought inappropriate to assume that populations drinking below the RCP limits would be at increased risk of chronic conditions such as alcoholic liver disease.

The resulting absolute risk function is therefore a function of consumption (for which a slope is defined) and threshold as follows:

$AR(c) = 0 \; if \; c < T$

$AR(c) = \beta \; (c - T) \;\; otherwise$ *Equation 6*

where AR = absolute risk, *c* = peak consumption level, *T* = threshold and *β* = slope parameter.

An example of a linear absolute risk function constructed from the number of deaths is presented in Figure 9.2. When using real data, the units on the vertical axis would be deaths. The key difference of the absolute risk function compared to RR function is that the absolute risk equals 0, rather than 1, when the peak day intake is below the threshold.

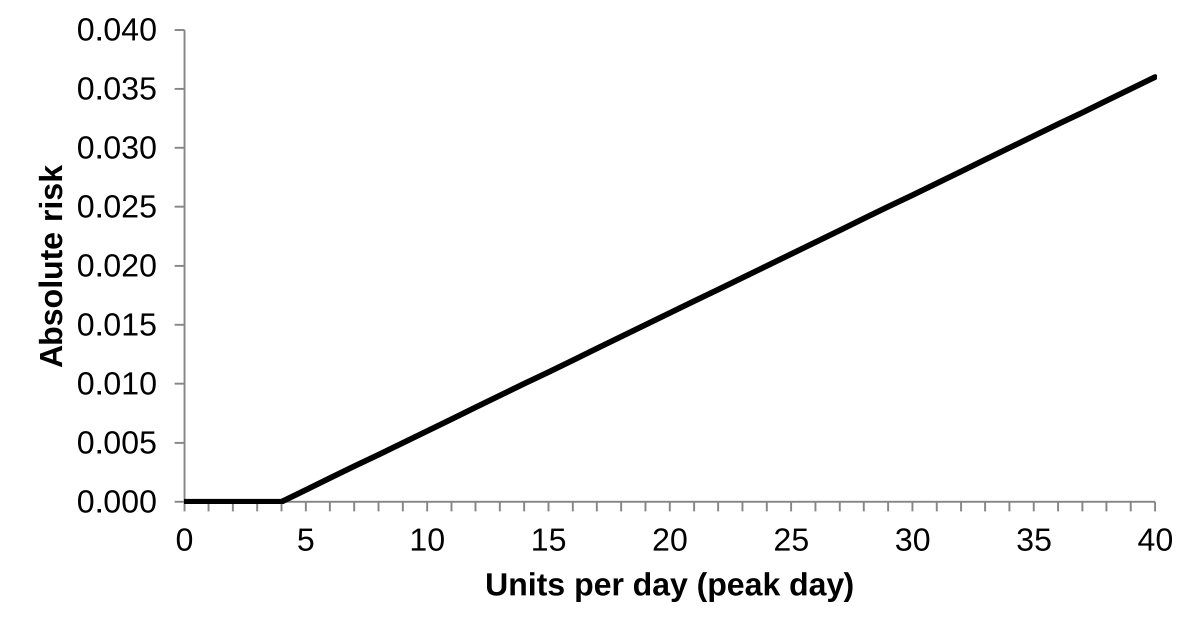

Figure 9.2: Illustrative linear absolute risk function for a wholly attributable acute harm (threshold of 4 units)

9.4 TIME LAG EFFECTS FOR CHRONIC HARMS

When modelling the link between consumption and harm, one important input is the assumption surrounding the ‘time lag’ – the time needed to achieve the full benefit (reduction in harms) associated with a reduction of consumption. Such data is necessary for chronic conditions where the development of diseases often occurs over many years.

Lag times used in the model are taken from a recent systematic review [55]. Lags are specified using three parameters: (1) the time to first effect from a consumption change; (2) the time to full effect and (3) the distribution of the total effect across the intervening time period. The full lag structures as implemented in the model are presented in Table 9.1. One potential limitation is the assumption that the time lag is similar for both morbidity and mortality which may or may not be true for all conditions. However in the absence of data and consensus, such an assumption had to be made.

These time lags are implemented in the model to calculate a lag-adjusted PIF, denoted $\overline{PIF}$, for health condition h and subgroup j at time $t = T$, from the unlagged PIFs as follows:

$$\overline{PIF_{Thj}} = \sum_{t=1}^{T} \lambda_{th} \, PIF_{thj} \qquad \textit{Equation 7}$$

where λ_{th} is the lag time from Table 9.1. Where the modelled policy only alters alcohol consumption in year 1, as is the case with all 4 policies modelled here, this simplifies to:

$$\overline{PIF_{Thj}} = PIF_{1hj} \sum_{t=1}^{T} \lambda_{th} \qquad \textit{Equation 8}$$

Table 9.1: Modelled lag times between changes in consumption and changes in mortality risk

Condition	Year from change in consumption																			
	1	2	3	4	5	6	7	8	9	10	11	12	13	14	15	16	17	18	19	20
Alcohol-induced pseudo-Cushing's syndrome	5%	5%	5%	5%	5%	5%	5%	5%	5%	5%	5%	5%	5%	5%	5%	5%	5%	5%	5%	5%
Degeneration of the nervous system	5%	5%	5%	5%	5%	5%	5%	5%	5%	5%	5%	5%	5%	5%	5%	5%	5%	5%	5%	5%
Alcoholic polyneuropathy	5%	5%	5%	5%	5%	5%	5%	5%	5%	5%	5%	5%	5%	5%	5%	5%	5%	5%	5%	5%
Alcoholic myopathy	5%	5%	5%	5%	5%	5%	5%	5%	5%	5%	5%	5%	5%	5%	5%	5%	5%	5%	5%	5%
Alcoholic cardiomyopathy	5%	5%	5%	5%	5%	5%	5%	5%	5%	5%	5%	5%	5%	5%	5%	5%	5%	5%	5%	5%
Alcoholic gastritis	50%	25%	13%	6%	3%	2%	1%	0%	0%	0%	0%	0%	0%	0%	0%	0%	0%	0%	0%	0%
Alcoholic liver disease	21%	13%	9%	7%	6%	5%	4%	4%	4%	3%	3%	3%	3%	3%	2%	2%	2%	2%	2%	2%
Acute pancreatitis	20%	16%	13%	10%	8%	7%	5%	4%	3%	3%	2%	2%	1%	1%	1%	1%	1%	0%	0%	0%
Chronic pancreatitis	20%	16%	13%	10%	8%	7%	5%	4%	3%	3%	2%	2%	1%	1%	1%	1%	1%	0%	0%	0%
Malignant neoplasm of lip, oral cavity and pharynx	0%	0%	0%	0%	0%	0%	0%	0%	0%	0%	10%	10%	10%	10%	10%	10%	10%	10%	10%	10%
Malignant neoplasm of oesophagus	0%	0%	0%	0%	0%	0%	0%	0%	0%	0%	10%	10%	10%	10%	10%	10%	10%	10%	10%	10%
Malignant neoplasm of colon and rectum	0%	0%	0%	0%	0%	0%	0%	0%	0%	0%	10%	10%	10%	10%	10%	10%	10%	10%	10%	10%
Malignant neoplasm of liver and intrahepatic bile ducts	0%	0%	0%	0%	0%	0%	0%	0%	0%	0%	10%	10%	10%	10%	10%	10%	10%	10%	10%	10%
Malignant neoplasm of larynx	0%	0%	0%	0%	0%	0%	0%	0%	0%	0%	10%	10%	10%	10%	10%	10%	10%	10%	10%	10%
Malignant neoplasm of breast	0%	0%	0%	0%	0%	0%	0%	0%	0%	0%	10%	10%	10%	10%	10%	10%	10%	10%	10%	10%
Epilepsy and status epilepticus	43%	26%	16%	9%	6%	0%	0%	0%	0%	0%	0%	0%	0%	0%	0%	0%	0%	0%	0%	0%
Hypertensive diseases	22%	18%	14%	11%	9%	7%	6%	5%	4%	3%	0%	0%	0%	0%	0%	0%	0%	0%	0%	0%
Cardiac arrhythmias	22%	18%	14%	11%	9%	7%	6%	5%	4%	3%	0%	0%	0%	0%	0%	0%	0%	0%	0%	0%
Haemorrhagic and other non-ischaemic stroke	31%	22%	15%	11%	7%	5%	4%	3%	2%	1%	0%	0%	0%	0%	0%	0%	0%	0%	0%	0%
Lower respiratory infections: pneumonia	61%	24%	10%	4%	1%	0%	0%	0%	0%	0%	0%	0%	0%	0%	0%	0%	0%	0%	0%	0%
Cirrhosis of the liver (excluding alcoholic liver disease)	20%	16%	13%	10%	8%	7%	5%	4%	3%	3%	2%	2%	1%	1%	1%	1%	1%	0%	0%	0%
Diabetes mellitus (type II)	22%	18%	14%	11%	9%	7%	6%	5%	4%	3%	0%	0%	0%	0%	0%	0%	0%	0%	0%	0%
Ischaemic heart disease	31%	22%	15%	11%	7%	5%	4%	3%	2%	1%	0%	0%	0%	0%	0%	0%	0%	0%	0%	0%
Ischaemic stroke	31%	22%	15%	11%	7%	5%	4%	3%	2%	1%	0%	0%	0%	0%	0%	0%	0%	0%	0%	0%

9.5 MORTALITY MODEL PARAMETERS

Age group and gender-specific mortality rates for each of the 43 modelled health conditions are derived from analysis published by the Centre for Public Health at Liverpool John Moores University [56] in combination with baseline population data from the ONS [57]. This population data is also used to create the baseline population for the life table, used to model transitions between alive and dead states. Other cause mortality is calculated by subtracting the total alcohol-related mortality rates for each subgroup from all-cause mortality rates taken from ONS mortality statistics for England & Wales for 2010 [58]. Where necessary risk functions were estimated using the methods described in Section 9.3.3 using HSE age/sex specific distributions of average weekly and peak day alcohol consumption.

9.6 DERIVING SOCIOECONOMIC CLASSIFICATION-SPECIFIC MORTALITY RATES

Investigation and adjustment of SAPM's subgroup-specific mortality and morbidity rates to account for variation in risk across socioeconomic (SEC) groups was undertaken in five steps, as detailed below. These steps follow in large part those used in previous versions of SAPM [59] although fewer socioeconomic groups are modelled (three rather than eight) and mortality rate adjustment factors are based on a narrower set of alcohol-related health conditions (those wholly attributable to alcohol rather than all conditions).

9.6.1 Step 1: Derive baseline age, gender and health condition-specific mortality rates for use as the basis for adjustments

As discussed in Section 9.5, age group and gender-specific mortality rates are calculated for every modelled health condition.

9.6.2 Step 2: Derivation of age, gender and SEC-specific adjustment factors for alcohol-related mortality rates.

Siegler *et al.* [60] derived age, gender and SEC group-specific alcohol-related mortality rates for England and Wales using 2001-2003 ONS data on deaths 'closely related to alcohol' (reproduced in Table 9.2 and Table 9.3). These are the most recent data of this kind available. As the age groups defined by Siegler *et al.* are different to those used in SAPM and Siegler *et al.* report for eight SEC groups whereas SAPM uses three SEC groups, population weights taken from HSE 2012 are used to allow estimation of rates for the modelled age and SEC groups (Table 9.4 and Table 9.5). Next, the alcohol-related mortality rate for each SEC group within an age and sex group is expressed as a ratio of the total alcohol-related mortality rate for that age and sex group (Table 9.6 and Table 9.7).

Table 9.2: Male alcohol-related mortality rates (per 100,000) by age and SEC classification, England and Wales, 2001-2003

	25-29	30-34	35-39	40-44	45-49	50-54	55-59	60-64
**NS-SEC1.1**	n/a	0.7	1.3	2.6	7.4	13.8	14.2	17.6
**NS-SEC1.2**	n/a	2.0	4.3	8.5	12.4	20.9	20.9	27.6
**NS-SEC2**	0.3	1.2	5.1	13.3	19.4	25.6	30.3	28.4
**NS-SEC3**	0.9	3.9	6.1	11.1	26.4	34.4	30.2	26.1
**NS-SEC4**	1.8	6.5	8.1	15.3	25.9	27.8	27.8	29.0
**NS-SEC5**	1.7	3.6	8.6	18.6	27.9	37.9	39.1	34.3
**NS-SEC6**	2.8	8.6	16.9	34.5	46.6	42.5	48.9	42.0
**NS-SEC7**	3.8	10.0	20.4	35.7	49.1	52.2	45.3	41.1
**All**	1.5	4.4	9.0	18.1	27.1	32.1	33.2	32.5

Source: Table 6 in Siegler et al.[1]

Table 9.3: Female alcohol-related mortality rates (per 100,000) by age and SEC classification, England and Wales, 2001-2003

	25-29	30-34	35-39	40-44	45-49	50-54	55-59	60-64
**NS-SEC1.1**	n/a	1.0	1.8	3.2	7.3	7.4	7.4	n/a
**NS-SEC1.2**	0.7	n/a	2.2	3.1	5.6	6.9	8.7	0.7
**NS-SEC2**	0.2	1.2	2.8	7.0	9.7	12.8	12.3	0.2
**NS-SEC3**	0.7	2.2	5.5	9.5	13.3	14.5	12.2	0.7
**NS-SEC4**	n/a	1.1	4.1	8.6	13.6	14.2	17.1	n/a
**NS-SEC5**	2.0	2.7	7.1	11.3	18.5	22.5	24.2	2.0
**NS-SEC6**	2.7	5.2	7.5	15.8	21.7	22.1	20.1	2.7
**NS-SEC7**	1.0	6.2	13.6	27.4	42.0	38.2	29.5	1.0
**All**	1.0	2.3	4.9	9.6	14.3	16.1	16.0	1.0

Source: Table 7 in Siegler et al.[1]

[1] Siegler *et al.* [60] use the National Statistics definition of alcohol-related deaths [63], those classified, under ICD-10, to: F10; G31.2; G62.1; I42.6; K29.2; K70; K73; K74 (excl. K74.3-K74.5); K86.0; X45; X65; or Y15.

Table 9.4: Males alcohol-related mortality rates (per 100,000) by SAPM age groups and SEC classification, England and Wales, 2001-2003

		16-24	25-34	35-54	55-89	All
**Professional/ managerial**	**NS-SEC1.1**<br>**NS-SEC1.2**<br>**NS-SEC2**	1.0	1.0	13.0	26.3	11.9
**Intermediate**	**NS-SEC3**<br>**NS-SEC4**	3.3	3.3	19.2	28.4	17.5
**Routine/ Manual**	**NS-SEC5**<br>**NS-SEC6**<br>**NS-SEC7**	5.2	5.2	33.9	42.1	27.0
**All**		3.0	3.0	21.6	32.9	18.7

Table 9.5: Females alcohol-related mortality rates (per 100,000) by SAPM age groups and SEC classification, England and Wales, 2001-2003

		16-24	25-34	35-54	55-89	All
**Professional/ managerial**	**NS-SEC1.1**<br>**NS-SEC1.2**<br>**NS-SEC2**	0.7	0.7	7.1	11.4	5.6
**Intermediate**	**NS-SEC3**<br>**NS-SEC4**	1.4	1.4	10.5	13.4	8.3
**Routine/ Manual**	**NS-SEC5**<br>**NS-SEC6**<br>**NS-SEC7**	3.6	3.6	20.9	22.7	15.5
**All**		1.7	1.7	11.2	16.0	8.9

Table 9.6: Male ratios of the mortality rate of each age-sex-SEC group to the mortality rate of the age-sex group, England and Wales, 2001-2003

	16-24	25-34	35-54	55-89
**Professional/ managerial**	0.3	0.3	0.6	0.8
**Intermediate**	1.1	1.1	0.9	0.9
**Routine/ Manual**	1.7	1.7	1.6	1.3
**All**	1.0	1.0	1.0	1.0

Table 9.7: Female ratios of the mortality rate of each age-sex-SEC group to the mortality rate of the age-sex group, England and Wales, 2001-2003

	16-24	25-34	35-54	55-89
**Professional/ managerial**	0.4	0.4	0.6	0.7
**Intermediate**	0.8	0.8	0.9	0.9
**Routine/ Manual**	2.1	2.1	1.9	1.4
**All**	1.0	1.0	1.0	1.0

9.6.3 Step 3: Estimation of SEC-specific alcohol-related mortality rates using SAPM

SAPM already implicitly accounts for differences in drinking patterns between SEC groups; therefore an assessment is required of the extent to which the differences in alcohol-related mortality between SEC groups seen in Table 9.6 and Table 9.7 are solely due to drinking patterns. This assessment is undertaken by modelling within SAPM a scenario where everybody stops drinking and extracting the resulting estimated reductions in mortality for wholly alcohol-attributable conditions. These results are then used to derive alcohol-related mortality rates for these conditions for each age, gender and SEC group. If the estimated alcohol-related mortality rates are not comparable to the patterns in Table 9.6 and Table 9.7, then this indicates the differential mortality rates reported by SAPM are not solely attributable to differences in drinking patterns across SEC groups. Adjustments to the underlying absolute alcohol-related mortality risks used in SAPM would then be required.

Unlike Table 9.6 and Table 9.7 the results from SAPM show higher alcohol-related mortality rates for higher SEC groups. Although contrary to the evidence from Siegler et al., this is expected as higher SEC groups drink more on average than lower SEC groups and, therefore, SAPM would be expected to estimate a higher alcohol-related mortality rate for higher SEC groups. Adjustments to SAPM to account for this are required.

Table 9.8: SAPM-derived estimated alcohol-related deaths wholly attributable to alcohol and mortality rates and ratios of the alcohol-attributable mortality rate of each SEC group to the alcohol-attributable rate of the population

	Alcohol-attributable deaths	Alcohol-attributable mortality rate (per 100,000)	Ratios of NS-SEC rate to total rate
**Managerial/professional**	1,511	9.9	0.8
**Intermediate**	1,153	10.9	0.9
**Routine/Manual**	2,401	14.0	1.2
**Total**	5,065	11.8	1.0

9.6.4 Step 4: Modifying adjustment factors to account for SEC-related differences in drinking patterns already modelled in SAPM

Adjustment factors are calculated by dividing the estimated age, gender and SEC group ratios in *Step 2* by the corresponding SEC group ratio in *Step 3*. For example, Table 9.7 gives a ratio of 0.41 for 25-34 year-old women in professional or managerial occupations based on evidence from Siegler et al. and the corresponding ratio of 0.84 is estimated for the same SEC group in the unadjusted version of SAPM. Therefore, the final adjustment factor for 25-34 year-old women in professional or managerial occupations is estimated to be 0.49 (0.41 / 0.84 = 0.49). The adjustment factors are shown in Table 9.9 and Table 9.10. These are applied to all of the conditions in Table 8.1 to derive age, gender and SEC group-specific mortality rates. For example, the original mortality rate for alcoholic liver disease for 25-34 year-old men is 2.5 per 100,000. Applying the adjustment factors gives a mortality rate of 1.0 per 100,000 for 25-34 year-old men in professional/managerial occupations (2.5 * 0.4 = 1.0) and 3.6 per 100,000 for counterparts in routine/manual occupations (2.5 * 1.5 = 3.6). As no data are available on socioeconomic differences in alcohol-related mortality for conditions partially attributable to alcohol, the approach taken in SAPM3 assumes the adjustment factors are the same for wholly alcohol-attributable and partially alcohol-attributable conditions.

Given the data reported by Siegler *et al.*, it is not possible to derive adjustment factors for the 16-24 year old age group. Therefore, it is assumed that the adjustment factors for this younger age groups are the same as for 25-34 year-olds

Table 9.9: Male adjustment factors for alcohol-related mortality rates by NS-SEC group

	16-24	25-34	35-54	55-89
**Professional/ managerial**	0.4	0.4	0.7	1.0
**Intermediate**	1.2	1.2	1.0	0.9
**Routine/ Manual**	1.5	1.5	1.3	1.1
**All**	1.0	1.0	1.0	1.0

Table 9.10: Female adjustment factors for alcohol-related mortality rates by NS-SEC group

	16-24	25-34	35-54	55-89
**Professional/ managerial**	0.5	0.5	0.8	0.8
**Intermediate**	0.9	0.9	1.0	0.9
**Routine/ Manual**	1.8	1.8	1.6	1.2
**All**	1.0	1.0	1.0	1.0

9.7 MORTALITY SELECTION IN SAPM

When modelling the relationship between exposure to a risk factor (e.g. alchol consumption) using a cohort-based approach as is the case in SAPM, an issue known as ‘mortality selection’ may introduce error into the estimated mortality outcomes, leading the model to overestimate the impact of an intervention. This was first described by Brønnum-Hansen who compared individual microsimulation and cohort-level models and identified that, as cohort models do not explicitly kill those with the greatest exposure (and therefore risk) within a cohort, the level of exposure in that cohort in later years is overestimated and therefore the model will overestimate deaths attributable to the risk factor in later years [61].

Whilst this issue is present in SAPM, the impact of it is likely to be small for a number of reasons. Firstly, the scale of the changes in exposure (i.e. alcohol consumption) are small compared to those modelled by Brønnum-Hansen, who modelled an intervention which reduced exposure for older cohorts to almost zero.

Secondly, there is not a clear gradient in changes in exposure across the modelled interventions/policies. That is to say that, unlike the scenario modelled by Brønnum-Hansen, people in SAPM do not experience a sudden reduction in exposure as they age, something which significantly exacerbates the error.

Finally, SAPM is a highly disaggregated model. Separate life tables are constructed and estimated for each gender (2), socioeconomic group (3) and drinking group (3). Thus instead of estimating annual exposure, risk and mortality rates across each age group, as in Brønnum-Hansen’s analysis, we estimate exposure, risk and mortality rates within each of 18 subgroups within each age group. This means that the heterogeneity in exposure within each group which gives rise to the issue of mortality selection is substantially reduced, particularly so since we stratify by the exposure by having separate life tables for each drinker group (within each age-gender-SES subgroup).

In conclusion, the issue of mortality selection is present within SAPM. To eradicate it would require moving to a fully individual-based model. As a result, both intervention and reference arms of the model will overestimate the number of alcohol-related deaths in later years of the model. However the error arises only where these overestimates are systematically different between the two arms. Further, this error is likely to be minor for the reasons outlined above.

10 PARTIAL MORTALITY RESULTS

All model results are presented for the 20th year following policy implementation as this represents the maximum time lag identified between changes in consumption and changes in harm. This is therefore the period after which the full effect of an intervention on health outcomes is reached. Table 10.1 below shows the change in deaths for the intervening years.

Table 10.1: Change in annual deaths in years 1-20 for all policies

		Current tax	Ad valorem tax	Volumetric tax	Minimum Unit Price
**Baseline deaths p.a.**		**12,190**			
**Absolute change in deaths per annum**	**Year 1**	-187	-189	-178	-165
	**Year 2**	-242	-242	-219	-209
	**Year 3**	-279	-278	-248	-241
	**Year 4**	-305	-303	-270	-265
	**Year 5**	-325	-323	-287	-284
	**Year 6**	-341	-338	-300	-300
	**Year 7**	-353	-350	-311	-312
	**Year 8**	-363	-359	-320	-323
	**Year 9**	-371	-367	-328	-331
	**Year 10**	-377	-373	-334	-338
	**Year 11**	-394	-391	-353	-356
	**Year 12**	-412	-410	-374	-377
	**Year 13**	-429	-427	-395	-398
	**Year 14**	-446	-444	-416	-418
	**Year 15**	-462	-461	-437	-439
	**Year 16**	-477	-477	-457	-459
	**Year 17**	-491	-491	-476	-478
	**Year 18**	-505	-504	-495	-495
	**Year 19**	-517	-517	-512	-513
	**Year 20**	-529	-530	-530	-530

11 DETERMINISTIC SENSITIVITY ANALYSIS

11.1 ALTERNATIVE ELASTICITY ESTIMATES

Her Majesty's Revenue and Customs (HMRC) have recently published new alcohol price elasticity estimates [62]. These estimates are derived using a cross-sectional methodology using Heckman, or 'double-hurdle', regression models, which is a very different approach to the quasi-longitudinal pseudo-panel models used in Meng et al. [15]. However, the estimates are produced in the same 10x10 beverage type and location split as the baseline elasticities used in the model and we are therefore able to use them in a deterministic sensitivity analysis to examine the impact that the use of alternative elasticity estimates has on the model results. The HMRC elasticities are presented in Table 11.1.

Table 11.1: HMRC elasticity estimates

		Purchase									
		Off-beer	Off-cider	Off-wine	Off-spirits	Off-RTD	On-beer	On-cider	On-wine	On-spirits	On-RTD
Price	Off-beer	-0.74*	0.07	-0.08*	-0.11*	-0.01	-0.08	-0.02	-0.02	-0.10	0.02
	Off-cider	-0.01	-0.74*	-0.09*	0.05	0.13	-0.06	-0.25*	-0.04	0.02	0.30*
	Off-wine	0.00	0.05	-0.08*	-0.07*	0.10	0.01	0.15*	0.03	-0.02	0.14*
	Off-spirits	0.04	0.13	-0.02	-0.45*	-0.09	0.00	-0.06	-0.01	-0.16*	-0.22
	Off-RTD	-0.03	-0.04	-0.03	-0.02	-0.52*	0.00	0.03	0.04	-0.03	-0.03
	On-beer	0.03	0.11	-0.04	0.08	0.11	-0.34*	0.05	0.10*	0.26*	0.08
	On-cider	0.05	-0.13	-0.06	0.10	0.24	-0.06	-0.49*	0.02	0.04	-0.04
	On-wine	0.02	-0.04	0.02	0.00	0.01	0.02	0.07	-0.24*	0.12*	-0.07
	On-spirits	-0.01	0.03	0.05*	0.01	0.00	-0.10*	-0.02	0.01	-1.25*	0.04
	On-RTD	0.09	0.00	0.00	0.05	-0.03	0.00	-0.02	-0.04	0.17*	-0.24*

Remarks: * p-value<0.05

Table 11.2: Meng et al. elasticity estimates (for comparison)

		Purchase									
		Off-beer	Off-cider	Off-wine	Off-spirits	Off-RTD	On-beer	On-cider	On-wine	On-spirits	On-RTD
Price	Off-beer	-0.980*	-0.189	0.096	-0.368	-1.092	-0.016	-0.050	0.253	0.030	0.503
	Off-cider	0.065	-1.268*	0.118	-0.122	-0.239	-0.053	0.093	0.067	-0.108	-0.194
	Off-wine	-0.040	0.736*	-0.384*	0.363	0.039	-0.245	-0.155	0.043	-0.186	0.110
	Off-spirits	0.113	-0.024	0.163	-0.082	-0.042	0.167	0.406	0.005	0.084	0.233
	Off-RTD	-0.047	-0.159	-0.006	0.079	-0.585*	-0.061	0.067	0.068	-0.179*	0.093
	On-beer	0.148	-0.285	0.115	-0.028	0.803	-0.786*	0.867	1.042*	1.169*	-0.117
	On-cider	-0.100	0.071	0.043	0.021	0.365	0.035	-0.591*	0.072	0.237*	0.241
	On-wine	-0.197	0.094	-0.154	-0.031	-0.093	-0.276	-0.031	-0.871*	-0.021	-0.363
	On-spirits	0.019	-0.117	-0.027	-0.280	-0.145	-0.002	-0.284	0.109	-0.890*	0.809*
	On-RTD	0.079	0.005	-0.085	-0.047	0.369	0.121	-0.394	-0.027	-0.071	-0.187

11.2 RESULTS

Table 11.3: Comparison of modelled impacts on consumption and deaths under baseline and alternative elasticitiy assumptions

**Alcohol consumption**						
**Consumption Breakdown**		**Elasticities**	**Current tax**	**Ad valorem tax**	**Volumetric tax**	**Minimum Unit Pricing**
All drinkers	All incomes	Meng et al 2014	-1.7%	-1.8%	-1.9%	-1.8%
		Sousa et al 2014	-2.5%	-1.7%	-3.2%	-4.7%
Moderate	All incomes	Meng et al 2014	-1.5%	-1.7%	-1.4%	-0.9%
		Sousa et al 2014	-2.2%	-1.6%	-2.2%	-2.6%
Increasing risk	All incomes	Meng et al 2014	-1.7%	-1.8%	-1.5%	-1.2%
		Sousa et al 2014	-2.5%	-1.7%	-2.9%	-4.3%
Heavy	All incomes	Meng et al 2014	-1.9%	-1.9%	-2.8%	-3.3%
		Sousa et al 2014	-2.7%	-1.8%	-4.5%	-7.3%
**Income Quintile Breakdown**						
Total drinker population	Q1 (low)	Meng et al 2014	-2.3%	-2.2%	-4.9%	-5.8%
		Sousa et al 2014	-2.9%	-1.9%	-4.0%	-7.4%
	Q2	Meng et al 2014	-2.1%	-2.2%	-2.7%	-2.8%
		Sousa et al 2014	-2.8%	-1.8%	-3.4%	-5.9%
	Q3	Meng et al 2014	-2.0%	-2.0%	-2.0%	-1.9%
		Sousa et al 2014	-2.6%	-1.7%	-3.0%	-4.6%
	Q4	Meng et al 2014	-1.6%	-1.7%	-1.0%	-0.3%
		Sousa et al 2014	-2.3%	-1.6%	-3.1%	-3.7%
	Q5 (high)	Meng et al 2014	-1.1%	-1.2%	0.1%	0.4%
		Sousa et al 2014	-2.1%	-1.4%	-2.7%	-3.1%
**Alcohol-Related Deaths**						
**Consumption Breakdown**						
All drinkers	All incomes	Meng et al 2014	-4.3%	-4.3%	-4.3%	-4.3%
		Sousa et al 2014	-6.3%	-4.1%	-8.7%	-13.5%
Moderate	All incomes	Meng et al 2014	1.4%	1.5%	1.3%	0.7%
		Sousa et al 2014	1.8%	1.4%	1.8%	1.9%
Increasing risk	All incomes	Meng et al 2014	-5.1%	-5.2%	-3.5%	-2.6%
		Sousa et al 2014	-7.3%	-4.7%	-8.7%	-13.3%
Heavy	All incomes	Meng et al 2014	-2.4%	-2.2%	-3.1%	-3.8%
		Sousa et al 2014	-3.5%	-2.2%	-5.7%	-9.2%
**Socioeconomic Breakdown**						
Total drinker population	Routine/manual	Meng et al 2014	-4.9%	-4.8%	-8.1%	-8.9%
		Sousa et al 2014	-5.9%	-3.8%	-7.8%	-13.3%
	Intermediate	Meng et al 2014	-4.3%	-4.3%	-2.5%	-2.2%
		Sousa et al 2014	-6.7%	-4.3%	-10.0%	-14.8%
	Professional/ managerial	Meng et al 2014	-3.3%	-3.6%	1.2%	2.4%
		Sousa et al 2014	-6.8%	-4.4%	-9.3%	-12.8%

12 PROBABILISTIC SENSITIVITY ANALYSIS

12.1 RATIONALE

SAPM3 is a deterministic model with over 1,000 distinct input parameters. There is limited or no information available to allow a full quantification of the uncertainty around many of these parameters and, more crucially, no information on the joint uncertainty (i.e. how parameter values may vary with respect to each other). However, we do have methods available to allow independent quantification (i.e. not allowing for joint uncertainty) of uncertainty around a number of key model parameter sets. We present here our methods for doing this and the results from 30 probabilistic sensitivity analysis (PSA) runs of SAPM3. SAPM is both computationally intensive (one model run takes approximately 20-25 minutes) and also time-consuming to set up (particularly when implementing the PSA methods outlined below) (set up can take 5-60 minutes), precluding the feasibility of a greater number of PSA runs. The fact that we have not accounted for uncertainty in all parameters will bias the uncertainty estimates presented here downwards (i.e. less uncertain), however this must be set against the fact that not accounting for correlation between parameters will bias the estimates upwards (i.e. more uncertain). In view of this, and the partial nature of the PSA, we still believe the deterministic model results to be our most robust estimates of the true impact of the modelled policies. However, the PSA results presented here highlight the potential impact of uncertainty on the conclusions of this study.

12.2 APPROACHES TO QUANTIFYING UNCERTAINTY

Our Partial PSA focuses on 5 key model input areas:

1. The baseline alcohol consumption data
2. The baseline alcohol purchasing/price data
3. The price elasticities of demand
4. The estimates of tax pass-through
5. The risk functions relating consumption level to mortality

We will describe our approach to uncertainty for each area in turn before presenting the overall results. For each PSA run we generate a new ‘alternate’ version of SAPM3 using the methods described below, then run all 4 modelled policies through this version of the model. Table 12.1 summarises the sampling methods and distributions.

12.2.1 Baseline consumption data

As described in Section 3, baseline alcohol consumption data for SAPM3 is taken from the Health Survey for England 2012. For each PSA run we take a nonparametric bootstrap

sample from the original HSE dataset which is the same size as the original sample. Essentially this means we recreate a new, sampled, HSE by sampling with replacement from the original HSE respondents. The sampled dataset then forms the baseline consumption data for use in the model.

12.2.2 Baseline price data

As described in Section 4, baseline alcohol purchasing and price data comes from the Living Costs and Food Survey (formerly the Expenditure and Food Survey) 2001-2009, adjusted to match aggregate sales data as described in Section 4.4. As for the baseline consumption data we take a nonparametric bootstrap sample from the original, adjusted, LCF dataset to form a new baseline purchasing and price dataset, which is used in the model to generate new subgroup-specific price distributions and consequently estimate the effect of pricing policies on these distributions.

12.2.3 Price elasticities

As described in Section 5, the price elasticities which relate changes in alcohol prices to changes in consumption, are taken from Meng *et al.* [15]. The authors provide standard errors around the central estimates in Table 2, and we can therefore sample from the error terms, assuming they are normally distributed, for each parameter in the 10x10 elasticity matrix (as in Table 5.1) to generate a new elasticity matrix for each PSA run.

12.2.4 Tax pass-through

As described in Section 6.2, estimates of the extent to which changes in taxation are passed through into the prices actually paid by consumers, and how this pass-through varies by beverage type and across the price distribution, are taken from Ally *et al.* [21]. The authors provide standard errors around the central estimates in Table S1, and we can therefore sample from the error terms as for the price elasticities to generate a new tax pass-through matrix similar to Table 6.3 for each PSA run.

12.2.5 Mortality risk functions

As discussed in Section 9.3.1, risk functions relating level of consumption with mortality risk for 17 partially alcohol-attributable health conditions are taken from a range of published literature sources (see Table 8.1 for references). These risk functions consist of continuous (usually) polynomial functions relating Relative Risk of mortality to alcohol consumption. For 15 of these health conditions the standard errors around all coefficients in the functions and their variance-covariance matrices were provided by the authors of the original studies. For

these conditions we sampled from the error terms, assuming normality, for every coefficient to obtain new functions for every PSA.

The remaining 2 studies (for cancer of the colon and rectum and cardiac arrhythmias) did not provide standard errors around the coefficients in these functions, but 95% confidence intervals around the point estimates at 3 levels of consumption (25, 50 and 100g of alcohol/day) are available from Corrao et al [29]. For each condition at each of these 3 levels we estimate the standard errors from the 95% confidence intervals assuming normality. We then draw one sample from each of the 3 points and fit a new polynomial function, of the same form as the original, to these points. We repeat this process 250 times and calculate the standard error around each polynomial coefficient from these empirical distributions. We then sample from these error distributions for every PSA run.

For both of the above methods, we sample the uncertainty separately for male and female risk functions, irrespective of whether or not the original risk functions are stratified by gender.

Table 12.1: Summary of sampling methods and sampled distributions for partial PSA:

Parameter group		Sampling method	Distribution(s)
**Baseline consumption data**		Nonparametric bootstrapping	Joint nonparametric distribution of age, gender, ethnicity, income quintile, SES, education, no. of children in household, employment status, mean alcohol consumption, peak day alcohol consumption in the last week and the 4-dimensional preference vector describing % of consumption which is beer/cider, wine, spirits and RTDs
**Baseline price data**			Joint nonparametric distribution of age, gender, income quintile, consumption group (moderate, increasing risk, heavy) and prices paid by beverage category (on- and off-trade beer, cider, wine, spirits and RTDs)*
**Price elasticities**		Random sampling	Joint 100-dimensional multivariate normal distribution of elasticity values
**Tax pass-through**			4 (one per beverage type) joint 11-dimensional multivariate normal distributions of pass-through rates
**Mortality risk functions**	**Tuberculosis**		2 (gender) normal distributions of polynomial coefficients
	**Malignant neoplasm of lip, oral cavity and pharynx**		2 (gender) joint 3-dimensional multivariate normal distributions of polynomial coefficients
	**Malignant neoplasm of oesophagus**		2 (gender) joint 2-dimensional multivariate normal distributions of polynomial coefficients
	**Malignant neoplasm of colon and rectum**		2 (gender) x 2 normal distributions of polynomial coefficients
	**Malignant neoplasm of liver and intrahepatic bile ducts**		2 (gender) joint 2-dimensional multivariate normal distributions of polynomial coefficients
	**Malignant neoplasm of larynx**		2 (gender) joint 2-dimensional multivariate normal distributions of polynomial coefficients
	**Malignant neoplasm of breast**		1 normal distribution of polynomial coefficients
	**Epilepsy and status epilepticus**		2 (gender) normal distributions of polynomial coefficients
	**Hypertensive disease**		2 (gender) joint 2-dimensional multivariate normal distributions of polynomial coefficients
	**Cardiac arrhythmias**		2 (gender) normal distributions of polynomial coefficients
	**Haemorrhagic and other non-ischaemic stroke**		2 (gender) normal distributions of polynomial coefficients
	**Pneumonia**		2 (gender) normal distributions of polynomial coefficients
	**Cirrhosis of the liver (excl. alcoholic liver disease)**		2 (gender) joint 2-dimensional multivariate normal distributions of polynomial coefficients
	**Acute and chronic pancreatitis**		2 (gender) normal distributions of polynomial coefficients
	**Diabetes mellitus (type II)**		2 (gender) joint 2-dimensional multivariate normal distributions of polynomial coefficients
	**Ischaemic heart disease**		2 (gender) joint 3-dimensional multivariate normal distributions of polynomial coefficients
	**Ischaemic stroke**		2 (gender) joint 2-dimensional multivariate normal distributions of polynomial coefficients

* Note that this data is also used within the model to split the 4-dimensional preference vectors for each individual in the consumption dataset into a 10-dimensional vector as discussed in Section 4.5

12.4 PSA RESULTS

Table 12.2: PSA impacts of modelled policies on alcohol consumption by drinker group

	All drinkers				Moderate drinkers				Increasing risk drinkers				Heavy drinkers			
PSA run	CT	AV	VT	MUP	CT	AV	VT	MUP	CT	AV	VT	MUP	CT	AV	VT	MUP
1	-2.8%	-3.2%	-6.5%	-4.8%	-2.6%	-3.0%	-5.1%	-2.5%	-2.8%	-3.2%	-6.1%	-4.0%	-3.1%	-3.4%	-8.2%	-7.7%
2	-0.5%	-1.4%	-1.7%	-2.1%	-1.1%	-1.7%	-1.4%	-1.5%	-0.5%	-1.4%	-1.4%	-1.2%	-0.1%	-1.0%	-2.3%	-3.6%
3	-1.9%	-2.1%	-0.5%	-1.2%	-2.0%	-2.2%	0.0%	-0.4%	-1.7%	-2.0%	-0.2%	-0.6%	-1.9%	-2.1%	-1.3%	-2.5%
4	-2.9%	-2.7%	-3.1%	-4.1%	-2.4%	-2.2%	-2.1%	-2.2%	-2.9%	-2.8%	-3.2%	-3.6%	-3.3%	-3.0%	-4.1%	-6.8%
5	-2.3%	-2.0%	-0.8%	-1.0%	-2.1%	-1.9%	-1.5%	-0.9%	-2.2%	-1.9%	0.1%	0.1%	-2.6%	-2.1%	-1.1%	-2.2%
6	-1.3%	-1.6%	-2.2%	-1.2%	-1.6%	-1.6%	-1.5%	-0.9%	-1.3%	-1.6%	-1.8%	-0.4%	-1.1%	-1.7%	-3.4%	-2.4%
7	-1.6%	-1.8%	-6.7%	-3.4%	-1.2%	-1.6%	-5.8%	-2.2%	-1.6%	-1.9%	-6.7%	-3.0%	-1.9%	-1.8%	-7.5%	-5.4%
8	-4.2%	-3.1%	-0.8%	-4.8%	-3.7%	-3.0%	0.0%	-2.3%	-4.4%	-3.2%	-0.4%	-4.2%	-4.5%	-3.2%	-2.1%	-8.1%
9	-2.6%	-2.4%	-0.3%	-1.0%	-1.8%	-1.8%	0.1%	-0.1%	-2.9%	-2.6%	-0.2%	-0.7%	-3.3%	-2.8%	-0.9%	-2.5%
10	-1.7%	-1.9%	-3.0%	-1.6%	-1.7%	-2.0%	-2.6%	-1.1%	-1.6%	-2.0%	-3.0%	-1.1%	-1.7%	-1.8%	-3.5%	-2.7%
11	-3.2%	-2.6%	-1.2%	-2.1%	-2.6%	-2.2%	-1.4%	-1.3%	-3.2%	-2.6%	-0.6%	-1.3%	-3.7%	-2.9%	-1.5%	-3.8%
12	-2.4%	-2.4%	-2.2%	-1.5%	-2.0%	-1.9%	-1.2%	-0.7%	-2.4%	-2.4%	-1.3%	-0.2%	-2.9%	-2.8%	-4.4%	-4.0%
13	-2.7%	-2.1%	-3.0%	-3.3%	-2.6%	-2.1%	-3.1%	-2.5%	-2.8%	-2.1%	-3.1%	-3.2%	-2.8%	-2.1%	-2.8%	-4.1%
14	-0.5%	-1.1%	-4.2%	-2.6%	-0.6%	-0.8%	-3.3%	-2.3%	-0.5%	-1.1%	-3.9%	-2.1%	-0.4%	-1.4%	-5.3%	-3.4%
15	-1.0%	-0.6%	-1.3%	-2.6%	-0.7%	-0.5%	-0.7%	-1.7%	-0.9%	-0.6%	-1.0%	-1.9%	-1.5%	-0.8%	-2.3%	-4.4%
16	-3.4%	-2.6%	-2.2%	-3.9%	-3.1%	-2.6%	-1.3%	-2.1%	-3.3%	-2.5%	-1.9%	-2.8%	-3.9%	-2.7%	-3.6%	-7.1%
17	-3.7%	-3.0%	-2.8%	-4.7%	-3.3%	-2.7%	-2.1%	-2.7%	-3.8%	-3.1%	-2.6%	-3.9%	-4.0%	-3.2%	-3.9%	-7.6%
18	-0.2%	-1.2%	-2.8%	-2.5%	-0.5%	-1.3%	-2.3%	-0.8%	-0.2%	-1.2%	-2.3%	-1.8%	0.1%	-1.0%	-3.8%	-5.1%
19	-2.1%	-2.5%	-2.7%	-2.0%	-1.8%	-2.2%	-2.3%	-0.9%	-2.1%	-2.6%	-2.6%	-1.5%	-2.3%	-2.7%	-3.3%	-3.7%
20	-3.1%	-2.9%	-3.7%	-3.3%	-3.0%	-2.6%	-2.4%	-1.8%	-3.0%	-2.9%	-3.3%	-2.1%	-3.3%	-3.2%	-5.4%	-6.2%
21	-3.7%	-3.2%	-5.7%	-7.1%	-2.8%	-2.6%	-4.6%	-3.5%	-3.7%	-3.2%	-5.7%	-6.5%	-4.5%	-3.6%	-6.5%	-11.1%
22	-0.6%	-0.5%	-3.2%	-2.4%	-0.2%	-0.3%	-2.6%	-1.1%	-0.6%	-0.4%	-2.4%	-1.6%	-1.1%	-0.7%	-4.7%	-4.7%
23	-0.7%	-0.8%	-2.3%	-1.9%	-0.3%	-0.6%	-2.0%	-0.6%	-0.7%	-0.7%	-1.6%	-1.1%	-1.2%	-1.0%	-3.5%	-4.4%
24	-0.3%	-1.7%	-1.5%	-3.5%	-0.2%	-1.6%	-0.6%	-1.3%	-0.3%	-1.8%	-1.6%	-2.8%	-0.4%	-1.8%	-2.4%	-6.3%
25	-1.8%	-1.9%	-4.4%	-3.6%	-1.4%	-1.7%	-3.1%	-1.9%	-1.9%	-2.0%	-3.8%	-2.3%	-2.0%	-2.0%	-6.1%	-6.5%
26	-3.2%	-3.0%	-1.2%	-1.9%	-3.1%	-2.8%	-1.3%	-1.5%	-3.3%	-3.1%	-1.0%	-1.5%	-3.2%	-3.1%	-1.2%	-2.7%
27	-3.1%	-2.5%	-4.1%	-3.8%	-2.5%	-2.1%	-3.1%	-2.0%	-2.9%	-2.4%	-3.7%	-2.7%	-3.9%	-3.0%	-5.5%	-6.9%
28	-2.1%	-2.6%	-2.1%	-3.7%	-2.4%	-2.6%	-2.0%	-2.2%	-2.3%	-2.8%	-2.2%	-3.1%	-1.6%	-2.2%	-2.1%	-6.0%
29	-1.5%	-1.5%	-2.4%	-2.5%	-0.9%	-1.3%	-1.4%	-0.7%	-1.6%	-1.6%	-2.2%	-2.1%	-1.9%	-1.7%	-3.7%	-4.8%
30	-1.6%	-2.2%	-3.2%	-1.2%	-1.8%	-2.1%	-1.8%	-0.5%	-1.6%	-2.2%	-2.9%	-0.5%	-1.3%	-2.2%	-4.9%	-2.6%

Table 12.3:PSA impacts of modelled policies on alcohol consumption by income quintile

	Income quintile 1 (lowest)				Income quintile 2				Income quintile 3				Income quintile 4				Income quintile 5 (highest)			
PSA run	CT	AV	VT	MUP	CT	AV	VT	MUP	CT	AV	VT	MUP	CT	AV	VT	MUP	CT	AV	VT	MUP
1	-3.3%	-3.6%	-10.2%	-9.6%	-3.2%	-3.6%	-7.2%	-6.1%	-3.4%	-3.7%	-6.7%	-5.4%	-2.5%	-3.0%	-5.6%	-3.2%	-2.2%	-2.6%	-4.3%	-1.8%
2	-0.8%	-1.7%	-4.2%	-6.2%	-1.0%	-1.7%	-2.3%	-2.5%	-0.7%	-1.5%	-1.8%	-2.2%	-0.3%	-1.1%	-0.7%	-1.4%	-0.2%	-0.9%	-0.2%	0.1%
3	-2.7%	-2.7%	-4.2%	-4.8%	-2.3%	-2.7%	-1.8%	-2.3%	-2.2%	-2.4%	-0.7%	-1.2%	-1.5%	-1.9%	0.4%	0.2%	-1.1%	-1.6%	1.6%	1.0%
4	-3.0%	-2.9%	-5.6%	-7.6%	-3.2%	-2.9%	-4.5%	-5.7%	-3.0%	-2.9%	-3.8%	-4.6%	-2.7%	-2.6%	-1.9%	-2.4%	-2.5%	-2.2%	-1.1%	-2.0%
5	-3.1%	-2.5%	-3.2%	-5.1%	-2.9%	-2.5%	-1.6%	-2.5%	-2.6%	-2.2%	-1.3%	-1.6%	-2.0%	-1.8%	-0.2%	0.6%	-1.4%	-1.3%	1.1%	1.9%
6	-1.9%	-2.0%	-4.1%	-4.2%	-1.6%	-2.0%	-2.5%	-2.8%	-1.5%	-1.8%	-2.1%	-0.8%	-1.3%	-1.7%	-1.8%	-0.3%	-0.7%	-1.1%	-1.2%	0.8%
7	-1.9%	-2.1%	-9.0%	-7.0%	-2.1%	-2.1%	-8.7%	-6.1%	-1.7%	-1.9%	-6.9%	-3.6%	-1.4%	-1.7%	-5.9%	-1.6%	-1.0%	-1.2%	-4.3%	-0.7%
8	-4.9%	-3.7%	-3.7%	-9.5%	-4.7%	-3.7%	-2.0%	-6.7%	-4.4%	-3.3%	-0.8%	-5.0%	-3.8%	-2.8%	0.4%	-2.7%	-3.6%	-2.5%	0.9%	-2.1%
9	-3.2%	-2.8%	-3.4%	-4.5%	-3.3%	-2.8%	-1.6%	-2.6%	-3.1%	-2.8%	-0.8%	-2.1%	-2.5%	-2.3%	1.0%	0.7%	-1.6%	-1.6%	1.8%	1.6%
10	-2.0%	-1.9%	-5.0%	-5.1%	-2.3%	-1.9%	-4.2%	-2.6%	-2.1%	-2.4%	-3.4%	-1.5%	-1.6%	-1.9%	-2.5%	-0.8%	-0.9%	-1.3%	-1.2%	0.6%
11	-4.2%	-3.5%	-4.8%	-7.6%	-3.6%	-3.5%	-2.6%	-3.1%	-3.3%	-2.8%	-0.9%	-1.3%	-3.0%	-2.4%	-0.2%	-0.3%	-2.2%	-1.6%	1.5%	0.5%
12	-3.2%	-2.9%	-6.5%	-7.8%	-2.8%	-2.9%	-3.2%	-2.9%	-2.4%	-2.4%	-1.7%	-0.4%	-2.4%	-2.4%	-0.9%	0.2%	-1.7%	-1.8%	0.0%	1.5%
13	-3.3%	-2.6%	-5.6%	-7.0%	-3.2%	-2.6%	-5.1%	-5.9%	-3.1%	-2.4%	-4.2%	-4.7%	-2.5%	-1.9%	-2.0%	-1.4%	-2.1%	-1.5%	-0.1%	-0.1%
14	-0.5%	-1.2%	-6.8%	-4.9%	-0.9%	-1.2%	-5.7%	-4.1%	-0.8%	-1.3%	-4.7%	-3.3%	-0.4%	-1.1%	-3.1%	-1.1%	-0.1%	-0.7%	-1.9%	-0.7%
15	-1.6%	-1.1%	-4.4%	-6.6%	-1.3%	-1.1%	-3.0%	-4.5%	-1.1%	-0.8%	-1.8%	-2.7%	-0.8%	-0.5%	0.2%	-0.7%	-0.5%	-0.1%	1.0%	-0.2%
16	-4.0%	-3.3%	-6.7%	-9.2%	-3.7%	-3.3%	-2.7%	-5.0%	-3.7%	-2.8%	-2.2%	-3.7%	-3.3%	-2.5%	-1.0%	-2.0%	-2.8%	-1.9%	0.4%	-1.0%
17	-4.1%	-3.2%	-4.7%	-8.9%	-4.3%	-3.2%	-3.8%	-6.5%	-4.0%	-3.2%	-2.7%	-4.9%	-3.6%	-3.0%	-2.4%	-3.2%	-3.0%	-2.4%	-1.4%	-1.9%
18	-0.9%	-1.8%	-7.3%	-6.4%	-0.3%	-1.8%	-3.1%	-3.8%	-0.6%	-1.5%	-2.8%	-3.0%	-0.2%	-1.2%	-1.7%	-0.6%	0.4%	-0.5%	-0.4%	-0.2%
19	-2.7%	-3.1%	-4.8%	-5.1%	-2.6%	-3.1%	-4.5%	-3.8%	-2.2%	-2.6%	-2.6%	-1.8%	-2.0%	-2.4%	-2.3%	-0.7%	-1.4%	-1.8%	-0.9%	-0.3%
20	-3.7%	-3.3%	-6.9%	-8.1%	-3.6%	-3.3%	-4.6%	-4.6%	-3.3%	-3.1%	-3.9%	-3.4%	-2.9%	-2.8%	-3.0%	-1.5%	-2.5%	-2.4%	-1.8%	-1.6%
21	-4.3%	-3.7%	-8.9%	-12.7%	-4.1%	-3.7%	-6.3%	-9.1%	-3.8%	-3.2%	-5.9%	-7.2%	-3.6%	-3.0%	-4.7%	-5.3%	-3.0%	-2.6%	-3.4%	-2.9%
22	-1.3%	-1.0%	-7.1%	-7.4%	-1.0%	-1.0%	-5.3%	-4.9%	-0.7%	-0.5%	-2.7%	-1.3%	-0.4%	-0.4%	-2.0%	-0.6%	0.0%	0.1%	-0.5%	0.3%
23	-0.9%	-1.1%	-4.8%	-5.5%	-1.0%	-1.1%	-3.6%	-3.1%	-1.0%	-1.0%	-2.7%	-2.3%	-0.7%	-0.7%	-1.4%	-0.4%	-0.3%	-0.2%	-0.3%	0.0%
24	-0.7%	-1.8%	-4.6%	-9.1%	-0.4%	-1.8%	-2.4%	-5.0%	-0.4%	-1.9%	-1.7%	-3.7%	-0.3%	-1.8%	-0.6%	-1.8%	-0.1%	-1.5%	0.1%	-0.7%
25	-2.0%	-2.3%	-8.5%	-9.3%	-2.1%	-2.3%	-5.0%	-4.2%	-2.2%	-2.2%	-4.4%	-3.6%	-1.5%	-1.7%	-3.1%	-1.2%	-1.4%	-1.4%	-1.9%	-0.8%
26	-3.7%	-3.3%	-4.4%	-7.2%	-3.8%	-3.3%	-3.1%	-4.3%	-3.4%	-3.2%	-1.1%	-1.5%	-2.9%	-2.9%	0.0%	0.6%	-2.5%	-2.5%	1.0%	0.7%
27	-4.4%	-3.4%	-7.1%	-9.2%	-3.6%	-3.4%	-4.6%	-4.8%	-3.3%	-2.7%	-4.2%	-4.6%	-2.7%	-2.3%	-3.4%	-1.8%	-2.1%	-1.7%	-2.2%	-0.6%
28	-2.2%	-2.6%	-4.2%	-7.8%	-2.7%	-2.6%	-2.8%	-5.3%	-2.6%	-3.0%	-2.4%	-2.9%	-2.0%	-2.5%	-1.6%	-2.4%	-1.3%	-1.8%	0.0%	-1.7%
29	-2.2%	-2.1%	-4.9%	-6.5%	-1.9%	-2.1%	-3.4%	-3.8%	-1.6%	-1.7%	-2.5%	-2.8%	-1.3%	-1.4%	-1.6%	-1.0%	-0.8%	-0.9%	-0.8%	0.1%
30	-1.8%	-2.4%	-6.9%	-5.5%	-1.9%	-2.4%	-4.4%	-2.6%	-1.7%	-2.4%	-3.3%	-1.4%	-1.4%	-2.1%	-2.3%	0.4%	-1.2%	-1.7%	-1.0%	1.0%

Table 12.4: PSA impacts of modelled policies on spending on alcohol by drinker group

	All drinkers				Moderate drinkers				Increasing risk drinkers				Heavy drinkers			
PSA run	CT	AV	VT	MUP	CT	AV	VT	MUP	CT	AV	VT	MUP	CT	AV	VT	MUP
1	2.0%	0.7%	-4.1%	-0.5%	1.7%	1.0%	-2.9%	-0.2%	2.1%	0.7%	-4.3%	-0.4%	2.2%	0.4%	-5.6%	-0.9%
2	3.2%	1.9%	-0.6%	1.5%	1.9%	1.6%	-0.3%	-0.1%	3.5%	1.9%	-0.7%	1.7%	4.8%	2.3%	-1.0%	3.3%
3	2.6%	1.6%	0.9%	2.9%	1.8%	1.6%	1.5%	1.5%	2.9%	1.7%	0.8%	3.1%	3.5%	1.6%	0.2%	4.6%
4	2.3%	1.9%	-2.0%	-0.2%	2.1%	2.3%	-0.7%	-0.5%	2.4%	1.7%	-2.5%	0.1%	2.7%	1.4%	-3.3%	-0.3%
5	2.4%	2.1%	-1.7%	1.6%	1.9%	2.2%	-2.6%	0.2%	2.6%	2.1%	-1.2%	2.1%	2.9%	1.9%	-1.0%	3.1%
6	2.7%	2.1%	-1.2%	2.1%	1.8%	2.2%	-0.5%	0.4%	2.8%	2.0%	-1.4%	2.6%	3.6%	1.9%	-2.0%	4.1%
7	3.6%	2.2%	-5.7%	0.0%	3.4%	2.6%	-4.1%	-0.3%	3.6%	2.1%	-6.5%	0.1%	3.8%	1.9%	-7.2%	0.3%
8	1.2%	1.1%	0.7%	0.0%	1.1%	1.3%	1.2%	0.0%	1.2%	1.0%	0.6%	0.2%	1.3%	0.8%	0.2%	-0.3%
9	3.0%	2.3%	1.2%	3.3%	3.5%	3.0%	1.8%	2.6%	3.0%	2.1%	0.8%	3.5%	2.5%	1.5%	0.8%	4.1%
10	3.2%	2.1%	-1.6%	2.2%	2.6%	2.1%	-0.9%	1.1%	3.5%	2.2%	-1.8%	2.4%	3.7%	2.1%	-2.3%	3.7%
11	2.0%	1.5%	-0.7%	1.7%	1.7%	1.9%	-1.1%	0.3%	2.1%	1.4%	-0.5%	2.0%	2.2%	1.2%	-0.5%	3.5%
12	2.8%	2.1%	-0.3%	2.5%	2.2%	2.4%	0.6%	1.1%	3.1%	2.1%	-0.3%	3.2%	3.1%	1.7%	-1.7%	3.6%
13	2.1%	1.9%	-1.8%	0.4%	1.5%	1.9%	-1.5%	-0.5%	2.3%	1.9%	-2.3%	0.2%	2.9%	2.0%	-1.7%	1.8%
14	4.1%	3.4%	-3.0%	-0.4%	3.1%	3.6%	-2.1%	-1.4%	4.3%	3.5%	-3.1%	-0.1%	5.0%	3.2%	-4.1%	0.8%
15	4.3%	3.6%	-0.9%	1.1%	3.7%	3.5%	-0.5%	-0.1%	4.5%	3.6%	-0.9%	1.5%	4.9%	3.5%	-1.5%	2.5%
16	1.5%	1.2%	-0.7%	0.1%	0.9%	1.0%	0.0%	-0.3%	1.8%	1.3%	-0.9%	0.4%	1.9%	1.3%	-1.3%	0.4%
17	1.3%	1.1%	-1.9%	-0.6%	1.0%	1.4%	-1.4%	-0.8%	1.4%	1.0%	-1.9%	-0.1%	1.5%	0.7%	-2.5%	-1.0%
18	3.9%	2.3%	-1.8%	2.4%	2.9%	2.1%	-1.3%	1.8%	4.0%	2.2%	-1.8%	2.8%	5.2%	2.6%	-2.4%	2.6%
19	2.7%	1.7%	-1.7%	1.5%	2.0%	1.8%	-1.0%	0.7%	3.0%	1.7%	-2.1%	1.4%	3.5%	1.5%	-2.0%	2.8%
20	1.3%	1.4%	-1.5%	0.1%	0.7%	1.7%	-0.2%	-0.3%	1.6%	1.4%	-1.7%	0.7%	1.8%	1.2%	-3.3%	-0.2%
21	2.1%	1.5%	-4.3%	-1.9%	2.2%	2.0%	-3.2%	-0.8%	2.1%	1.4%	-4.9%	-1.9%	1.8%	0.8%	-5.1%	-3.4%
22	4.8%	3.8%	-3.1%	1.3%	4.3%	3.9%	-2.2%	0.7%	5.0%	3.8%	-2.9%	1.6%	5.2%	3.5%	-4.7%	2.0%
23	4.5%	3.4%	-2.1%	2.3%	4.2%	3.6%	-1.8%	1.5%	4.8%	3.4%	-2.0%	2.5%	4.8%	3.1%	-2.7%	3.1%
24	4.5%	2.4%	0.6%	0.7%	3.7%	2.4%	1.6%	0.8%	4.7%	2.4%	0.3%	0.8%	5.4%	2.3%	-0.6%	0.5%
25	3.3%	2.3%	-2.3%	1.2%	3.0%	2.5%	-1.4%	0.4%	3.5%	2.2%	-2.4%	2.0%	3.6%	2.0%	-3.6%	1.3%
26	1.6%	1.1%	-0.7%	1.0%	0.9%	1.2%	-0.9%	-0.3%	1.6%	1.0%	-0.8%	1.0%	2.5%	1.0%	-0.1%	3.0%
27	2.1%	1.8%	-2.5%	0.1%	1.9%	2.1%	-1.6%	-0.2%	2.4%	1.9%	-2.8%	0.5%	2.0%	1.3%	-3.3%	0.0%
28	2.2%	1.2%	-1.8%	0.7%	1.5%	1.3%	-1.3%	0.0%	2.1%	1.0%	-2.2%	0.9%	3.3%	1.2%	-1.7%	1.7%
29	4.1%	2.9%	-0.5%	2.2%	3.9%	3.1%	0.6%	1.8%	4.2%	2.9%	-0.8%	2.1%	4.4%	2.7%	-1.6%	2.8%
30	2.8%	1.9%	-1.2%	2.1%	1.7%	1.9%	0.3%	0.7%	3.0%	1.8%	-1.4%	2.3%	4.2%	1.9%	-3.0%	3.9%

Table 12.5: PSA impacts of modelled policies on spending on alcohol by income quintile

	Income quintile 1 (lowest)				Income quintile 2				Income quintile 3				Income quintile 4				Income quintile 5 (highest)			
PSA run	CT	AV	VT	MUP	CT	AV	VT	MUP	CT	AV	VT	MUP	CT	AV	VT	MUP	CT	AV	VT	MUP
1	1.9%	0.4%	-7.1%	-1.8%	1.8%	0.4%	-4.8%	-0.7%	1.4%	0.1%	-4.5%	-1.2%	2.3%	0.9%	-3.7%	0.1%	2.4%	1.2%	-2.4%	0.4%
2	3.3%	1.7%	-2.3%	1.5%	2.9%	1.7%	-1.1%	2.3%	3.1%	1.7%	-1.0%	1.7%	3.4%	2.1%	0.0%	0.9%	3.3%	2.1%	0.3%	1.4%
3	2.0%	1.2%	-1.8%	2.3%	2.2%	1.2%	-0.4%	2.6%	2.4%	1.4%	0.4%	3.2%	2.7%	1.7%	1.6%	2.9%	3.1%	1.9%	2.5%	3.1%
4	2.4%	1.7%	-5.2%	-2.0%	2.1%	1.7%	-3.6%	-0.7%	2.2%	1.6%	-3.0%	-0.5%	2.4%	2.0%	-0.8%	0.6%	2.5%	2.2%	0.2%	0.5%
5	2.1%	1.9%	-3.1%	0.9%	1.9%	1.9%	-2.7%	1.0%	2.1%	1.9%	-2.4%	0.9%	2.6%	2.1%	-1.4%	2.1%	2.9%	2.4%	-0.1%	2.4%
6	2.3%	1.7%	-2.3%	1.4%	2.4%	1.7%	-1.8%	2.2%	2.4%	1.8%	-1.7%	2.7%	2.5%	2.1%	-0.8%	1.8%	3.2%	2.5%	-0.3%	2.1%
7	3.5%	2.1%	-7.6%	-1.6%	3.2%	2.1%	-7.8%	-1.1%	3.6%	2.2%	-6.6%	0.0%	3.6%	2.3%	-5.2%	0.7%	3.8%	2.6%	-3.5%	0.7%
8	0.8%	0.7%	-1.4%	-1.5%	0.9%	0.7%	-0.2%	-0.7%	1.2%	1.0%	0.5%	0.2%	1.4%	1.2%	1.4%	0.6%	1.4%	1.3%	1.8%	0.6%
9	2.3%	1.7%	-1.4%	2.6%	2.4%	1.7%	0.1%	3.2%	2.6%	2.0%	0.5%	2.6%	3.1%	2.4%	2.1%	3.8%	4.0%	3.0%	2.7%	3.9%
10	3.6%	2.4%	-4.1%	2.3%	2.8%	2.4%	-2.4%	2.3%	2.8%	1.8%	-1.9%	2.2%	3.1%	2.0%	-1.1%	2.0%	3.5%	2.5%	-0.1%	2.3%
11	1.3%	0.7%	-3.5%	-0.3%	1.6%	0.7%	-2.3%	1.8%	1.8%	1.3%	-0.9%	2.4%	2.0%	1.5%	-0.1%	2.0%	2.7%	2.4%	1.2%	2.2%
12	2.5%	1.8%	-3.1%	0.4%	2.6%	1.8%	-1.0%	2.4%	2.8%	2.1%	-0.5%	3.2%	2.7%	2.0%	0.5%	2.8%	3.0%	2.4%	1.0%	2.8%
13	2.3%	1.9%	-4.1%	-0.8%	1.9%	1.9%	-3.9%	-1.1%	1.7%	1.7%	-2.9%	-0.6%	2.2%	2.0%	-1.2%	1.1%	2.4%	2.2%	0.4%	1.6%
14	4.2%	3.7%	-5.6%	-1.3%	3.9%	3.7%	-4.7%	-1.4%	3.9%	3.3%	-3.6%	-0.5%	4.1%	3.4%	-2.3%	0.3%	4.1%	3.5%	-0.9%	0.1%
15	4.5%	3.6%	-3.8%	0.0%	4.3%	3.6%	-2.7%	0.5%	4.2%	3.4%	-1.7%	1.5%	4.3%	3.5%	0.4%	1.7%	4.3%	3.7%	1.2%	1.4%
16	1.0%	0.8%	-3.9%	-2.8%	1.4%	0.8%	-1.6%	-0.4%	1.3%	1.0%	-1.1%	0.4%	1.4%	1.2%	0.1%	0.5%	1.9%	1.6%	1.3%	1.6%
17	1.2%	1.1%	-3.4%	-1.5%	0.8%	1.1%	-2.4%	-1.0%	1.1%	1.0%	-2.2%	-0.7%	1.3%	1.0%	-1.6%	-0.3%	1.7%	1.5%	-0.8%	-0.1%
18	3.4%	1.7%	-5.3%	2.4%	4.1%	1.7%	-2.6%	2.7%	3.5%	1.9%	-2.4%	1.9%	3.8%	2.3%	-1.1%	2.9%	4.3%	2.9%	0.2%	2.1%
19	2.7%	1.3%	-3.5%	1.8%	2.2%	1.3%	-3.2%	0.6%	2.8%	1.5%	-2.1%	1.6%	2.6%	1.7%	-1.4%	1.6%	3.1%	2.2%	0.0%	1.8%
20	1.1%	1.4%	-3.5%	-1.5%	0.9%	1.4%	-2.3%	-0.4%	1.3%	1.2%	-2.2%	-0.1%	1.4%	1.4%	-1.2%	0.8%	1.6%	1.7%	-0.1%	0.6%
21	2.0%	1.3%	-7.0%	-3.9%	2.0%	1.3%	-5.4%	-2.8%	1.9%	1.3%	-5.0%	-2.3%	2.0%	1.5%	-3.6%	-1.6%	2.3%	1.8%	-2.4%	-0.2%
22	4.8%	3.5%	-6.6%	-0.4%	4.7%	3.5%	-5.5%	0.0%	4.9%	3.7%	-3.4%	2.5%	4.8%	3.7%	-2.3%	1.7%	4.8%	4.0%	-0.3%	2.0%
23	4.8%	3.4%	-4.4%	1.8%	4.5%	3.4%	-3.4%	2.3%	4.4%	3.1%	-2.9%	2.5%	4.4%	3.3%	-1.5%	2.6%	4.7%	3.7%	-0.4%	2.0%
24	4.5%	2.5%	-1.8%	-1.9%	4.6%	2.5%	-0.1%	0.3%	4.4%	2.3%	0.3%	0.2%	4.6%	2.3%	1.1%	1.2%	4.5%	2.4%	1.8%	2.0%
25	3.0%	1.8%	-5.0%	-0.8%	3.4%	1.8%	-3.5%	1.7%	3.2%	2.1%	-2.8%	1.3%	3.5%	2.5%	-1.5%	2.1%	3.5%	2.5%	-0.6%	1.3%
26	1.6%	1.1%	-3.4%	-1.0%	0.9%	1.1%	-2.3%	-0.4%	1.4%	1.0%	-1.1%	1.3%	1.7%	1.1%	-0.1%	2.1%	1.9%	1.3%	1.3%	1.7%
27	1.3%	1.3%	-4.4%	-1.8%	1.7%	1.3%	-3.0%	-0.3%	1.9%	1.7%	-2.9%	-0.2%	2.2%	1.8%	-2.1%	0.8%	2.7%	2.3%	-1.2%	0.8%
28	1.8%	0.9%	-3.7%	0.4%	1.9%	0.9%	-2.5%	0.6%	1.8%	0.8%	-2.4%	1.2%	2.1%	1.1%	-1.7%	0.9%	2.9%	1.7%	0.1%	0.6%
29	3.9%	2.7%	-2.4%	1.2%	3.8%	2.7%	-1.1%	2.1%	4.1%	2.9%	-1.1%	1.9%	4.2%	2.9%	0.2%	2.4%	4.5%	3.2%	0.7%	2.7%
30	2.9%	1.9%	-4.1%	1.2%	2.6%	1.9%	-2.5%	1.7%	2.7%	1.7%	-1.8%	1.9%	2.9%	1.9%	-0.5%	2.8%	2.8%	2.1%	0.8%	2.4%

Table 12.6: PSA impacts of modelled policies on annual deaths due to alcohol by drinker group

	All drinkers				Moderate drinkers				Increasing risk drinkers				Heavy drinkers			
PSA run	CT	AV	VT	MUP	CT	AV	VT	MUP	CT	AV	VT	MUP	CT	AV	VT	MUP
1	-980	-1072	-2261	-1928	-80	-94	-146	-66	-436	-499	-933	-616	-464	-479	-1182	-1246
2	-50	-329	-452	-645	-35	-51	-41	-19	-30	-173	-164	-120	15	-104	-247	-505
3	-497	-591	-260	-314	-44	-47	-9	-1	-252	-300	-61	-37	-201	-243	-190	-276
4	-844	-790	-853	-1261	-46	-44	-53	-42	-427	-404	-396	-499	-371	-341	-404	-720
5	-711	-592	-159	-258	-44	-39	-34	-16	-281	-244	124	147	-385	-309	-249	-389
6	-320	-465	-625	-362	-53	-51	-47	-10	-168	-231	-212	-21	-99	-183	-366	-331
7	-525	-556	-2066	-1245	-29	-42	-136	-47	-240	-262	-895	-420	-256	-253	-1036	-778
8	-1293	-904	-268	-1650	-84	-65	9	-46	-616	-437	-33	-583	-593	-402	-244	-1021
9	-955	-836	-103	-392	-44	-45	-2	-2	-459	-413	-19	-91	-452	-378	-81	-299
10	-486	-584	-923	-420	-53	-62	-80	-36	-243	-298	-420	-121	-190	-224	-423	-263
11	-1001	-766	-54	-481	-76	-68	-31	-34	-468	-373	18	-105	-456	-325	-41	-342
12	-687	-696	-563	-302	-44	-46	-43	-12	-314	-326	-103	45	-329	-324	-417	-335
13	-747	-579	-834	-967	-52	-42	-69	-54	-362	-283	-352	-385	-333	-255	-412	-529
14	-164	-389	-1412	-955	-18	-18	-83	-59	-72	-179	-579	-298	-74	-193	-749	-598
15	-346	-198	-408	-893	-19	-15	-20	-39	-149	-90	-124	-305	-178	-93	-263	-549
16	-1068	-753	-655	-1172	-84	-71	-28	-47	-513	-371	-234	-386	-470	-311	-394	-740
17	-1259	-1026	-966	-1696	-74	-62	-50	-50	-616	-497	-364	-619	-569	-467	-552	-1026
18	95	-274	-641	-919	-10	-33	-54	-3	43	-140	-209	-262	62	-101	-378	-653
19	-601	-743	-864	-552	-43	-51	-57	-19	-299	-368	-365	-189	-258	-323	-442	-344
20	-860	-848	-1184	-965	-69	-58	-62	-39	-417	-416	-485	-301	-374	-374	-636	-624
21	-1293	-1043	-1867	-2675	-69	-65	-115	-79	-582	-483	-848	-1017	-642	-495	-904	-1579
22	-268	-181	-870	-755	-4	-9	-66	-18	-109	-69	-262	-164	-155	-103	-542	-574
23	-286	-249	-678	-737	-11	-21	-63	-8	-114	-102	-185	-134	-161	-127	-430	-595
24	-87	-515	-583	-1213	-4	-26	-28	-31	-50	-265	-234	-449	-33	-224	-322	-734
25	-651	-655	-1622	-1468	-43	-50	-86	-41	-297	-308	-558	-385	-311	-297	-978	-1042
26	-999	-947	-305	-538	-66	-60	-35	-31	-489	-468	-126	-193	-444	-420	-144	-313
27	-937	-749	-1202	-1097	-56	-47	-65	-35	-403	-338	-508	-350	-477	-364	-630	-712
28	-589	-747	-500	-1187	-64	-70	-56	-61	-331	-403	-260	-476	-194	-273	-184	-650
29	-520	-476	-765	-875	-17	-27	-28	-5	-261	-247	-342	-338	-243	-201	-394	-531
30	-386	-633	-909	-154	-45	-55	-32	0	-194	-304	-314	83	-148	-274	-563	-237

Table 12.7: PSA impacts of modelled policies on annual deaths due to alcohol by socioeconomic group

	Routine/manual				Intermediate				Professional/managerial			
PSA run	CT	AV	VT	MUP	CT	AV	VT	MUP	CT	AV	VT	MUP
1	-579	-591	-1288	-1352	-196	-222	-447	-332	-205	-259	-526	-244
2	-135	-248	-404	-581	22	-52	-28	-69	63	-29	-20	5
3	-311	-321	-361	-477	-111	-139	-21	55	-76	-131	122	108
4	-375	-363	-637	-803	-197	-183	-160	-259	-272	-244	-56	-198
5	-475	-370	-394	-575	-152	-130	6	19	-84	-92	229	298
6	-221	-241	-341	-473	-70	-113	-151	-51	-30	-111	-134	162
7	-298	-332	-1222	-923	-118	-123	-450	-268	-108	-102	-395	-54
8	-587	-429	-316	-946	-326	-219	-40	-403	-381	-256	88	-302
9	-468	-404	-358	-495	-243	-206	28	-67	-244	-225	228	170
10	-306	-324	-616	-482	-97	-128	-160	-8	-83	-131	-147	70
11	-487	-419	-522	-635	-241	-174	120	-11	-272	-174	349	165
12	-342	-325	-571	-542	-166	-173	-36	56	-179	-198	44	184
13	-394	-315	-691	-762	-178	-136	-155	-210	-175	-128	13	5
14	-143	-192	-962	-870	-22	-89	-246	-97	0	-108	-203	12
15	-212	-148	-447	-697	-79	-46	-79	-163	-55	-4	118	-32
16	-472	-366	-555	-805	-267	-178	-112	-264	-328	-208	12	-104
17	-633	-518	-615	-1049	-293	-241	-208	-372	-333	-267	-143	-275
18	-84	-216	-629	-603	42	-51	-96	-209	137	-7	85	-106
19	-333	-381	-612	-541	-135	-172	-181	-83	-133	-189	-71	72
20	-465	-407	-737	-765	-185	-195	-224	-142	-210	-247	-224	-58
21	-582	-487	-1009	-1408	-336	-262	-463	-741	-375	-294	-395	-526
22	-195	-166	-912	-899	-44	-24	-85	-15	-29	10	127	159
23	-206	-216	-623	-643	-63	-46	-93	-118	-16	13	37	25
24	-49	-221	-487	-828	-25	-127	-69	-263	-13	-167	-27	-123
25	-330	-351	-1059	-1035	-167	-157	-344	-291	-154	-147	-219	-142
26	-556	-474	-568	-833	-200	-204	71	72	-243	-269	193	224
27	-501	-387	-682	-806	-211	-165	-250	-222	-224	-197	-270	-69
28	-392	-421	-513	-723	-111	-158	-35	-247	-87	-167	48	-217
29	-275	-258	-511	-623	-124	-113	-156	-177	-121	-105	-98	-74
30	-242	-303	-679	-466	-83	-150	-178	30	-61	-180	-52	281

Table 12.8: Mean PSA results with 95% Uncertainty Intervals for absolute changes in alcohol consumption

		Current tax	Ad valorem tax	Volumetric tax	Min. Unit Price
**Consumption level**		PSA mean change in units (95% UI)			
**All drinkers**		-15 (-28, -2)	-15 (-23, -4)	-19 (-46, -3)	-20 (-40, -7)
**Moderate**		-5 (-10, -1)	-6 (-9, -1)	-6 (-15, 0)	-4 (-8, -1)
**Increasing risk**		-29 (-55, -4)	-30 (-45, -7)	-34 (-88, -1)	-30 (-67, -1)
**Heavy**		-92 (-178, -1)	-89 (-139, -31)	-148 (-301, -41)	-198 (-351, -92)
**Income quintile x consumption level**					
**All drinkers**	**Q1 (low)**	-19 (-36, -5)	-19 (-28, -8)	-43 (-74, -23)	-53 (-85, -30)
	**Q2**	-15 (-27, -2)	-15 (-22, -6)	-23 (-46, -10)	-26 (-44, -14)
	**Q3**	-15 (-28, -3)	-15 (-24, -5)	-19 (-45, -5)	-20 (-41, -4)
	**Q4**	-13 (-26, -2)	-14 (-22, -3)	-13 (-40, 4)	-8 (-27, 4)
	**Q5 (high)**	-12 (-27, 1)	-13 (-22, 0)	-6 (-35, 14)	-3 (-19, 14)
**Moderate**	**Q1 (low)**	-6 (-10, -2)	-6 (-8, -2)	-9 (-17, -4)	-9 (-14, -5)
	**Q2**	-5 (-9, -1)	-5 (-8, -1)	-8 (-16, -2)	-6 (-11, -3)
	**Q3**	-6 (-11, -1)	-6 (-9, -2)	-6 (-16, 0)	-4 (-8, -1)
	**Q4**	-6 (-10, 0)	-6 (-9, -1)	-6 (-16, 2)	-2 (-6, 1)
	**Q5 (high)**	-4 (-10, 1)	-5 (-9, 0)	-2 (-12, 5)	-1 (-6, 3)
**Increasing risk**	**Q1 (low)**	-39 (-67, -12)	-39 (-56, -17)	-82 (-138, -45)	-89 (-136, -52)
	**Q2**	-37 (-64, -6)	-37 (-55, -14)	-56 (-111, -19)	-57 (-101, -20)
	**Q3**	-31 (-55, -5)	-31 (-46, -6)	-34 (-89, -1)	-29 (-72, 4)
	**Q4**	-28 (-54, -3)	-28 (-45, -6)	-21 (-79, 16)	-12 (-57, 17)
	**Q5 (high)**	-21 (-47, 1)	-22 (-36, -1)	-11 (-63, 23)	-2 (-31, 23)
**Heavy**	**Q1 (low)**	-125 (-239, -15)	-117 (-181, -52)	-325 (-532, -170)	-466 (-691, -257)
	**Q2**	-105 (-183, -9)	-102 (-155, -45)	-175 (-346, -49)	-254 (-421, -119)
	**Q3**	-105 (-184, -17)	-99 (-151, -44)	-158 (-290, -35)	-214 (-386, -58)
	**Q4**	-76 (-155, 10)	-78 (-124, -19)	-88 (-236, 27)	-82 (-232, 45)
	**Q5 (high)**	-59 (-147, 26)	-59 (-111, -1)	-22 (-166, 86)	-18 (-164, 115)

Table 12.9: Mean PSA results with 95% Uncertainty Intervals for relative changes in alcohol consumption

		Current tax	Ad valorem tax	Volumetric tax	Min. Unit Price
**Consumption level**		PSA mean % change in units (95% UI)			
**All drinkers**		-2.1% (-3.9%, -0.3%)	-2.1% (-3.2%, -0.6%)	-2.7% (-6.6%, -0.5%)	-2.8% (-5.4%, -1%)
**Moderate**		-1.9% (-3.4%, -0.2%)	-1.9% (-3%, -0.4%)	-2.1% (-5.3%, 0.1%)	-1.5% (-2.9%, -0.3%)
**Increasing risk**		-2.1% (-4%, -0.3%)	-2.1% (-3.2%, -0.5%)	-2.4% (-6.3%, -0.1%)	-2.1% (-4.8%, -0.1%)
**Heavy**		-2.3% (-4.5%, 0%)	-2.2% (-3.4%, -0.8%)	-3.7% (-7.7%, -1.1%)	-5% (-8.9%, -2.4%)
**Income quintile x consumption level**					
**All drinkers**	**Q1 (low)**	-2.6% (-4.6%, -0.7%)	-2.5% (-3.7%, -1.1%)	-5.7% (-9.4%, -3.3%)	-7.2% (-10.5%, -4.4%)
	**Q2**	-2.5% (-4.4%, -0.4%)	-2.5% (-3.6%, -0.9%)	-3.9% (-7.6%, -1.6%)	-4.4% (-7.4%, -2.4%)
	**Q3**	-2.3% (-4.1%, -0.5%)	-2.3% (-3.4%, -0.7%)	-2.9% (-6.8%, -0.7%)	-3% (-5.9%, -0.7%)
	**Q4**	-1.9% (-3.7%, -0.2%)	-2% (-3%, -0.4%)	-1.8% (-5.7%, 0.6%)	-1.2% (-3.8%, 0.6%)
	**Q5 (high)**	-1.5% (-3.2%, 0.1%)	-1.5% (-2.6%, -0.1%)	-0.7% (-4.3%, 1.7%)	-0.3% (-2.3%, 1.7%)
**Moderate**	**Q1 (low)**	-2.5% (-4.1%, -0.7%)	-2.4% (-3.4%, -0.8%)	-3.9% (-7%, -1.5%)	-3.8% (-5.8%, -2%)
	**Q2**	-2.2% (-3.8%, -0.5%)	-2.2% (-3.4%, -0.5%)	-3.2% (-6.8%, -1%)	-2.6% (-4.7%, -1.1%)
	**Q3**	-2.1% (-3.6%, -0.4%)	-2.1% (-3.2%, -0.6%)	-2.2% (-5.6%, 0.1%)	-1.5% (-2.9%, -0.2%)
	**Q4**	-1.7% (-3.2%, -0.1%)	-1.9% (-2.9%, -0.4%)	-1.8% (-5%, 0.5%)	-0.8% (-2%, 0.4%)
	**Q5 (high)**	-1.3% (-2.7%, 0.3%)	-1.3% (-2.4%, 0.1%)	-0.5% (-3.4%, 1.3%)	-0.3% (-1.5%, 1%)
**Increasing risk**	**Q1 (low)**	-2.8% (-4.6%, -0.8%)	-2.7% (-3.9%, -1.2%)	-5.7% (-9.7%, -3.3%)	-6.2% (-9.2%, -3.7%)
	**Q2**	-2.7% (-4.6%, -0.4%)	-2.6% (-3.9%, -1%)	-3.9% (-7.8%, -1.4%)	-4.1% (-7.1%, -1.5%)
	**Q3**	-2.2% (-4%, -0.4%)	-2.2% (-3.4%, -0.4%)	-2.5% (-6.5%, -0.1%)	-2.1% (-5.2%, 0.3%)
	**Q4**	-2% (-3.9%, -0.2%)	-2% (-3.2%, -0.5%)	-1.5% (-5.6%, 1.2%)	-0.9% (-4%, 1.2%)
	**Q5 (high)**	-1.5% (-3.3%, 0.1%)	-1.6% (-2.6%, 0%)	-0.8% (-4.5%, 1.7%)	-0.2% (-2.2%, 1.6%)
**Heavy**	**Q1 (low)**	-2.6% (-5.1%, -0.3%)	-2.4% (-3.9%, -1%)	-6.8% (-10.8%, -3.8%)	-9.8% (-14.8%, -5.6%)
	**Q2**	-2.7% (-4.9%, -0.2%)	-2.6% (-3.9%, -1.2%)	-4.5% (-8.2%, -1.1%)	-6.6% (-11.5%, -2.8%)
	**Q3**	-2.8% (-4.9%, -0.5%)	-2.6% (-4%, -1.2%)	-4.2% (-7.8%, -1%)	-5.7% (-10.1%, -1.6%)
	**Q4**	-2% (-4.1%, 0.3%)	-2.1% (-3.3%, -0.5%)	-2.4% (-6.7%, 0.7%)	-2.2% (-6.2%, 1.2%)
	**Q5 (high)**	-1.6% (-3.8%, 0.8%)	-1.6% (-3%, 0%)	-0.6% (-4.6%, 2.4%)	-0.5% (-4.3%, 3.1%)

Table 12.10: Mean PSA results with 95% Uncertainty Intervals for absolute changes in spending on alcohol

		Current tax	Ad valorem tax	Volumetric tax	Min. Unit Price
**Consumption level**		PSA mean change in £GBP (95% UI)			
**All drinkers**		18 (8, 29)	13 (6, 23)	-10 (-30, 6)	7 (-6, 19)
**Moderate**		7 (3, 14)	7 (3, 12)	-3 (-11, 5)	1 (-3, 6)
**Increasing risk**		36 (16, 59)	24 (11, 44)	-21 (-65, 9)	16 (-10, 40)
**Heavy**		96 (41, 153)	52 (18, 99)	-66 (-174, 10)	51 (-49, 120)
**Income quintile x consumption level**					
**All drinkers**	**Q1 (low)**	15 (5, 27)	10 (4, 21)	-22 (-42, -8)	-1 (-20, 14)
	**Q2**	13 (4, 23)	9 (3, 18)	-14 (-32, 0)	3 (-9, 14)
	**Q3**	15 (7, 27)	11 (4, 20)	-12 (-31, 3)	6 (-9, 19)
	**Q4**	19 (9, 30)	13 (7, 24)	-6 (-27, 12)	10 (-4, 20)
	**Q5 (high)**	26 (13, 39)	20 (10, 32)	1 (-23, 22)	13 (-1, 28)
**Moderate**	**Q1 (low)**	5 (1, 10)	5 (2, 8)	-6 (-12, 0)	-1 (-7, 6)
	**Q2**	5 (2, 10)	5 (2, 9)	-5 (-13, 2)	0 (-4, 6)
	**Q3**	7 (2, 13)	6 (2, 11)	-4 (-12, 4)	2 (-3, 6)
	**Q4**	8 (3, 15)	8 (4, 13)	-2 (-11, 8)	2 (-3, 8)
	**Q5 (high)**	11 (5, 20)	11 (6, 18)	3 (-8, 12)	2 (-5, 12)
**Increasing risk**	**Q1 (low)**	30 (10, 55)	20 (4, 44)	-52 (-99, -17)	1 (-33, 37)
	**Q2**	28 (9, 54)	17 (2, 38)	-33 (-77, -3)	7 (-35, 37)
	**Q3**	34 (16, 57)	22 (9, 44)	-25 (-71, 7)	18 (-16, 43)
	**Q4**	37 (17, 58)	24 (11, 42)	-12 (-56, 20)	22 (-8, 45)
	**Q5 (high)**	44 (23, 66)	31 (16, 51)	-5 (-45, 29)	21 (1, 41)
**Heavy**	**Q1 (low)**	92 (23, 160)	50 (11, 110)	-135 (-250, -59)	-1 (-120, 113)
	**Q2**	88 (17, 167)	47 (9, 91)	-87 (-200, -12)	48 (-67, 170)
	**Q3**	78 (26, 143)	38 (2, 86)	-92 (-206, -13)	32 (-96, 139)
	**Q4**	103 (45, 165)	56 (25, 107)	-46 (-161, 42)	71 (-48, 160)
	**Q5 (high)**	115 (54, 174)	66 (27, 102)	7 (-102, 94)	96 (4, 194)

Table 12.11: Mean PSA results with 95% Uncertainty Intervals for relative changes in spending on alcohol

		Current tax	Ad valorem tax	Volumetric tax	Min. Unit Price
**Consumption level**		PSA mean % change in £GBP (95% UI)			
**All drinkers**		2.8% (1.3%, 4.6%)	2% (1%, 3.6%)	-1.5% (-4.7%, 1%)	1% (-0.9%, 3%)
**Moderate**		2.3% (0.8%, 4.2%)	2.2% (1%, 3.7%)	-0.9% (-3.4%, 1.6%)	0.3% (-1%, 2%)
**Increasing risk**		3% (1.4%, 4.8%)	2% (0.9%, 3.7%)	-1.7% (-5.3%, 0.8%)	1.3% (-0.8%, 3.3%)
**Heavy**		3.3% (1.4%, 5.2%)	1.8% (0.6%, 3.5%)	-2.3% (-6%, 0.4%)	1.8% (-1.7%, 4.2%)
**Income quintile x consumption level**					
**All drinkers**	**Q1 (low)**	2.6% (1%, 4.8%)	1.8% (0.6%, 3.6%)	-4% (-7.2%, -1.4%)	-0.2% (-3.1%, 2.5%)
	**Q2**	2.5% (0.8%, 4.6%)	1.8% (0.6%, 3.5%)	-2.7% (-6.1%, -0.1%)	0.7% (-1.8%, 2.8%)
	**Q3**	2.6% (1.1%, 4.6%)	1.8% (0.6%, 3.5%)	-2.1% (-5.5%, 0.5%)	1% (-1.5%, 3.2%)
	**Q4**	2.8% (1.3%, 4.6%)	2% (1%, 3.6%)	-0.9% (-4.1%, 1.7%)	1.5% (-0.6%, 3.2%)
	**Q5 (high)**	3.1% (1.6%, 4.7%)	2.4% (1.3%, 3.8%)	0.2% (-2.7%, 2.6%)	1.6% (-0.2%, 3.3%)
**Moderate**	**Q1 (low)**	2.2% (0.5%, 4.3%)	2% (0.8%, 3.7%)	-2.8% (-5.1%, -0.1%)	-0.6% (-2.9%, 2.5%)
	**Q2**	2.2% (0.6%, 4.4%)	2.1% (0.9%, 3.9%)	-2.2% (-5.1%, 0.9%)	0% (-1.9%, 2.5%)
	**Q3**	2.2% (0.7%, 4.3%)	2% (0.8%, 3.6%)	-1.2% (-4.2%, 1.4%)	0.5% (-1.1%, 2.2%)
	**Q4**	2.3% (0.8%, 4%)	2.1% (1%, 3.6%)	-0.5% (-3.1%, 2.1%)	0.6% (-0.8%, 2.2%)
	**Q5 (high)**	2.5% (1%, 4.5%)	2.5% (1.2%, 4%)	0.6% (-1.7%, 2.8%)	0.5% (-1.1%, 2.6%)
**Increasing risk**	**Q1 (low)**	2.6% (0.9%, 4.8%)	1.8% (0.4%, 3.7%)	-4.5% (-8.4%, -1.5%)	0.1% (-2.7%, 3.1%)
	**Q2**	2.4% (0.8%, 4.7%)	1.5% (0.1%, 3.3%)	-2.9% (-6.6%, -0.3%)	0.6% (-3.1%, 3.3%)
	**Q3**	2.9% (1.4%, 5%)	1.9% (0.7%, 3.9%)	-2.2% (-6%, 0.6%)	1.6% (-1.3%, 3.7%)
	**Q4**	3.1% (1.4%, 4.9%)	2% (0.9%, 3.6%)	-1% (-4.8%, 1.7%)	1.8% (-0.7%, 3.9%)
	**Q5 (high)**	3.3% (1.7%, 4.8%)	2.3% (1.2%, 3.8%)	-0.3% (-3.4%, 2.1%)	1.6% (0.1%, 3.1%)
**Heavy**	**Q1 (low)**	3.1% (0.8%, 5.2%)	1.7% (0.4%, 3.6%)	-4.5% (-8.6%, -2%)	0% (-4%, 4.2%)
	**Q2**	3.1% (0.7%, 6%)	1.7% (0.4%, 3.4%)	-3.1% (-7.8%, -0.4%)	1.7% (-2.5%, 5.3%)
	**Q3**	2.9% (0.9%, 5.2%)	1.4% (0.1%, 3.2%)	-3.3% (-7.1%, -0.5%)	1.2% (-3.4%, 5.1%)
	**Q4**	3.5% (1.6%, 5.6%)	1.9% (0.9%, 3.5%)	-1.6% (-5.7%, 1.4%)	2.5% (-1.6%, 5.4%)
	**Q5 (high)**	3.9% (1.8%, 5.8%)	2.2% (0.9%, 3.7%)	0.2% (-3.6%, 3.2%)	3.3% (0.2%, 6.4%)

Table 12.12: Mean PSA results with 95% Uncertainty Intervals for absolute changes in annual deaths (full effect)

		PSA mean change in number of deaths p.a. (95% CI)			
		**Current tax**	**Ad valorem tax**	**Volumetric tax**	**Min. unit price**
**Population**		-644 (-1293, -10)	-640 (-1051, -193)	-828 (-2120, -89)	-936 (-2133, -230)
**Routine/manual**		-143 (-376, 84)	-154 (-276, 11)	-43 (-431, 262)	-20 (-363, 286)
**Intermediate**		-146 (-329, 27)	-146 (-247, -40)	-141 (-453, 84)	-162 (-496, 60)
**Professional/managerial**		-355 (-599, -75)	-340 (-538, -161)	-644 (-1240, -334)	-755 (-1367, -471)
**Moderate**	**Routine/manual**	-11 (-28, 3)	-12 (-25, 0)	-7 (-33, 9)	-5 (-17, 5)
	**Intermediate**	-10 (-19, -1)	-10 (-17, -2)	-9 (-28, 5)	-6 (-15, 3)
	**Professional/managerial**	-26 (-40, -4)	-26 (-41, -10)	-38 (-75, -11)	-21 (-41, -4)
**Increasing risk**	**Routine/manual**	-79 (-195, 34)	-84 (-153, 0)	-12 (-210, 181)	5 (-164, 169)
	**Intermediate**	-71 (-147, 7)	-72 (-110, -17)	-56 (-201, 47)	-54 (-200, 59)
	**Professional/managerial**	-156 (-271, -34)	-154 (-246, -67)	-247 (-494, -110)	-237 (-441, -107)
**Heavy**	**Routine/manual**	-53 (-159, 53)	-58 (-111, 14)	-24 (-196, 127)	-20 (-189, 138)
	**Intermediate**	-66 (-163, 24)	-64 (-132, -13)	-77 (-228, 55)	-102 (-303, 59)
	**Professional/managerial**	-174 (-297, -29)	-160 (-256, -77)	-359 (-700, -179)	-496 (-871, -303)

Table 12.13: Mean PSA results with 95% Uncertainty Intervals for relative changes in annual deaths (full effect)

		PSA mean % change in number of deaths p.a. (95% CI)			
		**Current tax**	**Ad valorem tax**	**Volumetric tax**	**Min. unit price**
**Population**		-5.3% (-10.2%, -0.1%)	-5.2% (-8.3%, -1.4%)	-6.7% (-15.8%, -0.7%)	-7.5% (-15.2%, -2.1%)
**Routine/manual**		-5.5% (-9.5%, -1.1%)	-5.3% (-7.7%, -2.5%)	-10% (-17.8%, -5.4%)	-11.7% (-18.2%, -7.5%)
**Intermediate**		-5.5% (-11.4%, 0.9%)	-5.4% (-9.5%, -1.4%)	-5.2% (-15.9%, 3.3%)	-5.8% (-16.7%, 2.6%)
**Professional/managerial**		-4.8% (-13.2%, 2.1%)	-5% (-10%, 0.3%)	-1.2% (-13.5%, 10.4%)	-0.4% (-11.1%, 10.1%)
**Moderate**	**Routine/manual**	4.3% (0.5%, 14.2%)	4.5% (1.2%, 14.7%)	6.4% (1.1%, 17.8%)	3.5% (0.4%, 10%)
	**Intermediate**	1.2% (0.1%, 3%)	1.2% (0.3%, 2.7%)	1% (-0.5%, 3.5%)	0.7% (-0.4%, 2.1%)
	**Professional/managerial**	0.8% (-0.3%, 2.6%)	0.9% (0%, 2.5%)	0.6% (-0.9%, 3%)	0.4% (-0.4%, 1.6%)
**Increasing risk**	**Routine/manual**	-6.7% (-11.5%, -1.5%)	-6.6% (-9.3%, -3%)	-10.6% (-19.3%, -4.7%)	-10.1% (-16.6%, -4.4%)
	**Intermediate**	-6.2% (-12.3%, 0.5%)	-6.3% (-10.9%, -1.2%)	-4.8% (-19%, 4.2%)	-4.6% (-16.8%, 5.6%)
	**Professional/managerial**	-5.3% (-12.6%, 1.9%)	-5.6% (-10.7%, 0%)	-0.6% (-12.5%, 11.5%)	0.5% (-10.7%, 12.1%)
**Heavy**	**Routine/manual**	-3.6% (-6.1%, -0.6%)	-3.3% (-5.1%, -1.7%)	-7.5% (-13.7%, -3.9%)	-10.3% (-16.7%, -7%)
	**Intermediate**	-2.7% (-5.7%, 1%)	-2.6% (-4.2%, -0.6%)	-3.1% (-8.2%, 2.3%)	-4.1% (-10.3%, 2.3%)
	**Professional/managerial**	-1.8% (-4.7%, 1.6%)	-1.9% (-3.6%, 0.4%)	-0.8% (-6.9%, 4%)	-0.7% (-5.6%, 3.6%)

REFERENCE LIST

1. Goddard E. National Statistics Methodological Series No. 37: Estimating alcohol consumption from survey data: updated method of converting volumes to units. Newport; 2007.

2. Stockwell T, Donath S, Cooper-Stanbury M, Chikritzhs T, Catalano P, Mateo C. Under-reporting of alcohol consumption in household surveys: a comparison of quantity-frequency, graduated-frequency and recent recall. Addiction. 2004;99: 1024–33. doi:10.1111/j.1360-0443.2004.00815.x

3. Goddard E. General Household Survey: Smoking and drinking among adults, 2005. Newport; 2006.

4. HM Revenue & Customs. Alcohol Factsheet March 2010. HMRC; 2010.

5. Meier PS, Meng Y, Holmes J, Baumberg B, Purshouse R, Hill-McManus D, et al. Adjusting for unrecorded consumption in survey and per capita sales data: quantification of impact on gender- and age-specific alcohol-attributable fractions for oral and pharyngeal cancers in Great Britain. Alcohol Alcohol. 2013;48: 241–9. doi:10.1093/alcalc/agt001

6. Townshend J, Duka T. Patterns of alcohol drinking in a population of young social drinkers: a comparison of questionnaire and diary measures. Alcohol Alcohol. 2002;37: 187–192.

7. Public Health England. Local Alcohol Profiles for England 2015 user guide. London; 2015.

8. Hill-McManus D, Angus C, Meng Y, Holmes J, Brennan A, Sylvia Meier P. Estimation of usual occasion-based individual drinking patterns using diary survey data. Drug Alcohol Depend. 2014;134: 136–143. doi:10.1016/j.drugalcdep.2013.09.022

9. Heckman J. Sample selection bias as a specification error. Econometrica. 1979;47: 153–161.

10. Office for National Statistics. Family spending 2010 edition: A report on the Living Costs and Food Survey 2009. Newport; 2012.

11. Robinson M, Beeston C, Mackison D. Monitoring and Evaluating Scotland's Alcohol Strategy: an update of alcohol sales and price band analysis. Edinburgh; 2012.

12. Gooding P. Consumer Prices Index and Retail Prices Index: the 2013 basket of goods and services. Newport; 2013.

13. Office for National Statistics. Consumer Price Inflation Reference Tables. 2014.

14. Meng Y, Brennan A, Holmes J, Hill-McManus D, Angus C, Purshouse R, et al. Modelled income group-specific impacts of alcohol minimum unit pricing in England 2014/15: Policy appraisals using new developments to the Sheffield Alcohol Policy Model (v2.5). 2013.

15. Meng Y, Brennan A, Purshouse R, Hill-McManus D, Angus C, Holmes J, et al. Estimation of own and cross price elasticities of alcohol demand in the UK-A pseudo-panel approach using the Living Costs and Food Survey 2001-2009. J Health Econ. 2014;34: 96–103. doi:10.1016/j.jhealeco.2013.12.006

16. HM Revenue & Customs. Alcohol Duty Rates. HMRC; 2014.

17. Leicester A. Alcohol pricing and taxation policies. London; 2011. doi:10.1920/bn.ifs.2011.00124

18. Black H, Gill J, Chick J. The price of a drink: levels of consumption and price paid per unit of alcohol by Edinburgh's ill drinkers with a comparison to wider alcohol sales in Scotland. Addiction. 2011;106: 729–36. doi:10.1111/j.1360-0443.2010.03225.x

19. Brennan A, Meng Y, Holmes J, Hill-McManus D, Meier PS. Potential benefits of minimum unit pricing for alcohol versus a ban on below cost selling in England 2014: modelling study. BMJ. 2014;349: g5452. doi:10.1136/bmj.g5452

20. HM Revenue & Customs. Excise notice 162: cider production. HMRC; 2014.

21. Ally AK, Meng Y, Chakraborty R, Dobson PW, Seaton JS, Holmes J, et al. Alcohol tax pass-through across the product and price range: do retailers treat cheap alcohol differently? Addiction. 2014; doi:10.1111/add.12590

22. Meng Y, Holmes J, Hill-McManus D, Brennan A, Meier PS. Trend analysis and modelling of gender-specific age, period and birth cohort effects on alcohol abstention and consumption level for drinkers in Great Britain using the General Lifestyle Survey 1984-2009. Addiction. 2014;109: 206–215. doi:10.1111/add.12330

23. Rehm J, Mathers C. Global burden of disease and injury and economic cost attributable to alcohol use and alcohol-use disorders. Lancet. 2009;373: 2223–33.

24. Rehm J, Baliunas D, Borges G. The relation between different dimensions of alcohol consumption and burden of disease: an overview. Addiction. 2010;105: 817–843. doi:10.1111/j.1360-0443.2010.02899.x

25. Lönnroth K, Williams BG, Stadlin S, Jaramillo E, Dye C. Alcohol use as a risk factor for tuberculosis - a systematic review. BMC Public Health. 2008;8: 289. doi:10.1186/1471-2458-8-289

26. Tramacere I, Negri E, Bagnardi V, Garavello W, Rota M, Scotti L, et al. A meta-analysis of alcohol drinking and oral and pharyngeal cancers. Part 1: overall results and dose-risk relation. Oral Oncol. Elsevier Ltd; 2010;46: 497–503. doi:10.1016/j.oraloncology.2010.03.024

27. Rota M, Bellocco R, Scotti L, Tramacere I, Jenab M, Corrao G, et al. Random-effects meta-regression models for studying nonlinear dose-response relationship, with an application to alcohol and esophageal squamous cell carcinoma. Stat Med. 2010;29: 2679–87. doi:10.1002/sim.4041

28. Fedirko V, Tramacere I, Bagnardi V, Rota M, Scotti L, Islami F, et al. Alcohol drinking and colorectal cancer risk: an overall and dose-response meta-analysis of published studies. Ann Oncol. 2011;22: 1958–72. doi:10.1093/annonc/mdq653

29. Corrao G, Bagnardi V, Zambon A, La Vecchia C. A meta-analysis of alcohol consumption and the risk of 15 diseases. Prev Med (Baltim). 2004;38: 613–9. doi:10.1016/j.ypmed.2003.11.027

30. Islami F, Tramacere I, Rota M, Bagnardi V, Fedirko V, Scotti L, et al. Alcohol drinking and laryngeal cancer: overall and dose-risk relation--a systematic review and meta-analysis. Oral Oncol. Elsevier Ltd; 2010;46: 802–10. doi:10.1016/j.oraloncology.2010.07.015

31. Key J, Hodgson S, Omar RZ, Jensen TK, Thompson SG, Boobis AR, et al. Meta-analysis of studies of alcohol and breast cancer with consideration of the methodological issues. Cancer causes Control CCC. 2006;17: 759–70. doi:10.1007/s10552-006-0011-0

32. Samokhvalov A V, Irving H, Mohapatra S, Rehm J. Alcohol consumption, unprovoked seizures, and epilepsy: a systematic review and meta-analysis. Epilepsia. 2010;51: 1177–84. doi:10.1111/j.1528-1167.2009.02426.x

33. Taylor B, Irving HM, Baliunas D, Roerecke M, Patra J, Mohapatra S, et al. Alcohol and hypertension: gender differences in dose-response relationships determined through systematic review and meta-analysis. Addiction. 2009;104: 1981–90. doi:10.1111/j.1360-0443.2009.02694.x

34. Kodama S, Saito K, Tanaka S, Horikawa C, Saito A, Heianza Y, et al. Alcohol consumption and risk of atrial fibrillation: a meta-analysis. J Am Coll Cardiol. Elsevier Inc.; 2011;57: 427–36. doi:10.1016/j.jacc.2010.08.641

35. Patra J, Taylor B, Irving H, Roerecke M, Baliunas D, Mohapatra S, et al. Alcohol consumption and the risk of morbidity and mortality for different stroke types--a systematic review and meta-analysis. BMC Public Health. 2010;10: 258. doi:10.1186/1471-2458-10-258

36. Samokhvalov A V, Irving H, Rehm J. Alcohol consumption as a risk factor for pneumonia: a systematic review and meta-analysis. Epidemiol Infect. Cambridge University Press; 2010;138: 1789–1795.

37. Rehm J, Taylor B, Mohapatra S, Irving H, Baliunas D, Patra J, et al. Alcohol as a risk factor for liver cirrhosis: a systematic review and meta-analysis. Drug Alcohol Rev. 2010;29: 437–45. doi:10.1111/j.1465-3362.2009.00153.x

38. Irving HM, Samokhvalov A V, Rehm J. Alcohol as a risk factor for pancreatitis. A systematic review and meta-analysis. JOP. 2009;10: 387–92.

39. Baliunas DO, Taylor BJ, Irving H, Roerecke M, Patra J, Mohapatra S, et al. Alcohol as a risk factor for type 2 diabetes: A systematic review and meta-analysis. Diabetes Care. 2009;32: 2123–32. doi:10.2337/dc09-0227

40. Roerecke M, Rehm J. The cardioprotective association of average alcohol consumption and ischaemic heart disease: a systematic review and meta-analysis. Addiction. 2012;107: 1246–60. doi:10.1111/j.1360-0443.2012.03780.x

41. Taylor BJ, Shield KD, Rehm JT. Combining best evidence: a novel method to calculate the alcohol-attributable fraction and its variance for injury mortality. BMC Public Health. 2011;11: 265. doi:10.1186/1471-2458-11-265

42. Purshouse R, Brennan A, Latimer N, Meng Y, Rafia R, Jackson R, et al. Modelling to assess the effectiveness and cost-effectiveness of public health related strategies and interventions to reduce alcohol attributable harm in England using the Sheffield Alcohol Policy Model version 2.0. Report to the NICE Public Health Programme Development Group. Sheffield; 2009.

43. Angus C, Meng Y, Ally AK, Holmes J, Brennan A. Model-based appraisal of minimum unit pricing for alcohol in the Republic of Ireland. Sheffield; 2014.

44. Angus C, Meng Y, Ally AK, Holmes J, Brennan A. Model-based appraisal of minimum unit pricing for alcohol in Northern Ireland. Sheffield; 2014.

45. Gunning-Schepers L. The health benefits of prevention: a simulation approach. Health Policy (New York). 1989;12: 1–255.

46. Fillmore KM, Kerr WC, Stockwell T, Chikritzhs T, Bostrom A. Moderate alcohol use and reduced mortality risk: Systematic error in prospective studies. Addict Res Theory. Informa UK Ltd UK; 2006;14: 101–132. doi:10.1080/16066350500497983

47. Roerecke M, Rehm J. Irregular heavy drinking occasions and risk of ischemic heart disease: a systematic review and meta-analysis. Am J Epidemiol. 2010;171: 633–44. doi:10.1093/aje/kwp451

48. Roerecke M, Rehm J. Alcohol consumption, drinking patterns, and ischemic heart

disease: a narrative review of meta-analyses and a systematic review and meta-analysis of the impact of heavy drinking occasions on risk for moderate drinkers. BMC Med. 2014;12: 182. doi:10.1186/s12916-014-0182-6

49. Bagnardi V, Zatonski W, Scotti L, La Vecchia C, Corrao G. Does drinking pattern modify the effect of alcohol on the risk of coronary heart disease? Evidence from a meta-analysis. J Epidemiol Community Health. 2008;62: 615–9. doi:10.1136/jech.2007.065607

50. Shield K, Parry CDH, Rehm J. Focus on: Chronic diseases and conditions related to alcohol use. Alcohol Res Curr Rev. 2014;35: 155–173.

51. Hill-McManus D, Angus C, Meng Y, Holmes J, Brennan A, Meier P. Injury alcohol-attributable fractions: methodological issues and developments. Sheffield; 2014;

52. Watson PE, Watson ID, Batt RD. Prediction of Blood Alcohol Concentrations in Human Subjects1. J oj Stud Alcohol. 1981;42.

53. Posey D, Mozayani A. The estimation of blood alcohol concentration. Forensic Sci Med Pathol. Springer; 2007;3: 33–39.

54. Royal College of Physicians. Royal College of Physicians' written evidence to Science and Technology Select Committee Inquiry on alcohol guidelines. London: Royal College of Physicians; 2011.

55. Holmes J, Meier PS, Booth A, Guo Y, Brennan A. The temporal relationship between per capita alcohol consumption and harm: a systematic review of time lag specifications in aggregate time series analyses. Drug Alcohol Depend. 2012;123: 7–14. doi:10.1016/j.drugalcdep.2011.12.005

56. Jones L, Bellis M. Updating England-specific alcohol-attributable fractions. Liverpool; 2013.

57. Office for National Statistics. Mid-year population estimates. Newport: Office for National Statistics; 2012.

58. Office for National Statistics. Deaths registered in England and Wales Series DR. Newport: Office for National Statistics; 2012.

59. Holmes J, Meng Y, Meier PS, Brennan A, Angus C, Campbell-Burton A, et al. Effects of minimum unit pricing for alcohol on different income and socioeconomic groups: a modelling study. Lancet. 2014;383: 1655–64. doi:10.1016/S0140-6736(13)62417-4

60. Siegler V, Al-Hamad A, B J, C W, Sheron N. Social inequalities in alcohol-related adult mortality by National Statistics Socio-economic Classifications, England and Wales 2001-03. Heal Stat Q. 2011;50.

61. Bronnum-Hansen H. How good is the Prevent model for estimating the health benefits of prevention? J Epidemiol Community Heal. 1999;53: 300–305. doi:10.1136/jech.53.5.300

62. Sousa J. Estimation of price elasticities of demand for alcohol in the United Kingdom. London; 2014. Report No.: 16.

63. Office for National Statistics. Alcohol-related deaths in the United Kingdom, registered in 2012: Statistical bulletin. Newport; 2014.
