# Supplementary material for: Estimated Effects of Different Alcohol Taxation and Price Policies on Health Inequalities: A Mathematical Modelling Study
Source: PLoS Med. 2016 Feb 23;13(2):e1001963. doi: 10.1371/journal.pmed.1001963 (PMC4764336; doi:10.1371/journal.pmed.1001963)
Supplement: S1 Data — (DOCX) [file pmed.1001963.s002.docx]

**Table S1_Data. Data sources for model inputs**

| Model Input | Name &  Year(s) | Detail | Source | Access link |
| --- | --- | --- | --- | --- |
| Baseline consumption | Health Survey for England (HSE), 2012 | Individual-level nationally-representative alcohol consumption and demographic data | UK Data Archive | https://discover.ukdataservice.ac.uk/catalogue/?sn=7480&type=Data%20catalogue |
| Binge consumption parameters | Not applicable | Regression parameters to estimate patterns of drinking from individual alcohol consumption and demographic data | Previously published analyses by the authors [[26](#_ENREF_26)] | Tables 3, 4, 5 & 6  http://www.sciencedirect.com/science/article/pii/S0376871613004018# |
| Prices paid | Living Cost and Food Survey (LCF) - previously the Expenditure and Food Survey, 2001-2009 | Individual-level nationally-representative alcohol purchasing data (individual transaction level) and demographic data | Individual transaction-level data: Department for Environment Food and Rural Affairs (DEFRA) | Access was negotiated via the team responsible for commissioning LCF at DEFRA:  <https://www.gov.uk/government/organisations/department-for-environment-food-rural-affairs> |
| Off-trade price distribution | Nielsen market research data, 2013 | Aggregate national off-trade sales data by price band (price-per-unit of alcohol) | NHS Health Scotland/Nielsen | [Off-trade price band distribution data_2009-2013_December 2014_1.xls](http://www.healthscotland.com/uploads/documents/24485-Off-trade%20price%20band%20distribution%20data_2009-2013_December%202014_1.xls) from  <http://www.healthscotland.com/documents/24485.aspx> |
| On-trade price distribution | CGA market research data, 2009 | Aggregate national on-trade sales data by price band (price-per-unit of alcohol) | CGA Strategy | Contact company directly to purchase data:  <http://www.cgastrategy.co.uk/> |
| Tax pass-through by price band | Not applicable | Differential tax pass-through rates of alcohol taxes, by product type and price band (i.e. estimates of the effect of a given tax change on retail prices) | Previously published analyses by the authors [[27](#_ENREF_27)] | Supplementary Table S1 from  <http://onlinelibrary.wiley.com/doi/10.1111/add.12590/abstract> |
| ONS historic inflation data | National statistics, 2001-2014 | Annual year-on-year inflation rate to harmonise costs | Office for National Statistics | <http://www.ons.gov.uk/ons/datasets-and-tables/downloads/xls-download.xls?dataset=mm23> |
| Price elasticities | Not applicable | Own- and cross-price elasticities of demand for on- and off-trade beer, cider, wine, spirits and Ready-to-Drinks (alcopops) | Previously published analyses by the authors [[28](#_ENREF_28)] | Table 3 in  <http://www.sciencedirect.com/science/article/pii/S0167629613001835> |
| Mid-year population estimates | National statistics, 2012 | Baseline age-gender population distribution | Office for National Statistics | <http://www.ons.gov.uk/ons/publications/re-reference-tables.html?edition=tcm%3A77-319259> |
| All-cause mortality statistics/  rates | National statistics, 2010 | All-cause mortality rates for all modelled age-gender subgroups | Office for National Statistics | <http://www.ons.gov.uk/ons/rel/vsob1/mortality-statistics--deaths-registered-in-england-and-wales--series-dr-/2012/dr-table5-2012.xls> |
| Lag time for chronic harms | Not applicable | Quantification of time lags between changes in alcohol consumption and changes in morbidity/mortality rates for all modelled chronic health conditions | Previously published analyses by the authors [[29](#_ENREF_29)] | Table 2 in  <http://www.sciencedirect.com/science/article/pii/S0376871611005278> |
| Alcohol-related mortality rates | Analysis of national health data, 2010 | Condition-specific mortality rates for all modelled age-gender subgroups | Centre for Public Health, Liverpool John Moores University [[30](#_ENREF_30)] | Tables 2 & 3  <http://www.cph.org.uk/wp-content/uploads/2014/03/24892-ALCOHOL-FRACTIONS-REPORT-A4-singles-24.3.14.pdf> |
| Socio-economic gradient in harm | Analysis of national health data, 2001-2003 | Quantification of the socioeconomic gradient in alcohol-related mortality | Office for National Statistics [[31](#_ENREF_31)] | Tables 8 & 9 in  <http://www.ncbi.nlm.nih.gov/pubmed/21647087>  (also see S1 Tables 9.2 to 9.7) |
